# Effect of ring rize on photoisomerization properties of stiff stilbene macrocycles

**DOI:** 10.3762/bjoc.15.233

**Published:** 2019-10-11

**Authors:** Sandra Olsson, Óscar Benito Pérez, Magnus Blom, Adolf Gogoll

**Affiliations:** 1 Department of Chemistry-BMC, Uppsala University, S-751 23 Uppsala, Sweden; 2 Faculty of Chemistry, Universitat de Barcelona, C/ Martí i Franquès 1, 08028 Barcelona, Spain

**Keywords:** DFT, molecular mechanics, photostability, photo-switch, ring-strain, stiff stilbene

## Abstract

A series of stiff stilbene macrocycles have been studied to investigate the possible impact of the macrocycle ring size on their photodynamic properties. The results show that reducing the ring size counteracts the photoisomerization ability of the macrocycles. However, even the smallest macrocycle studied (stiff stilbene subunits linked by a six carbon chain) showed some degree of isomerization when irradiated. DFT calculations of the energy differences between the *E*- and *Z*-isomers show the same trend as the experimental results. Interestingly the DFT study highlights that the energy difference between the *E*- and *Z*-isomers of even the largest macrocycle (linked by a twelve carbon chain) is significantly higher than that of the stiff stilbene unit itself. In general, it is indicated that addition of even a flexible chain to the stiff stilbene unit may significantly affect its photochemical properties and increase the photostability of the resulting macrocycle.

## Introduction

**Scheme 1:**
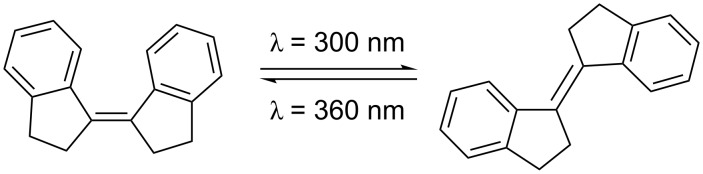
The stiff stilbene photoisomerization from *Z* to *E* and vice versa by irradiation at 300 nm and 360 nm, respectively.

The stiff stilbene (SS) molecule has drawn a lot of interest due to its photodynamic properties [1]. Stiff stilbenes typically undergo light triggered isomerization from *Z* to *E* at 300 nm and from *E* to *Z* at 360 nm (Scheme 1) [2]. The photochemical mechanism of this reaction is thoroughly described by Quick et al. [2]. The stiff stilbenes ability to photoisomerize has made it a useful building block of photodynamic triggers, switches and machines [3–11]. The interplay between the forces involved in the switching action and the pull from groups attached to the stiff stilbene has been investigated, e.g., as molecular force probes [12–18]. While these do incorporate other isomerizable units in addition to stiff stilbene, we were interested in the effect that the length of an *n*-alkane chain connecting the two halves of stiff stilbene might have. Similar studies, with stilbene and pyrene as the modulating units, have recently been published [19–20]. Our group has reported a SS-based bis-metalloporphyrin molecular tweezer that binds ditopically to guest molecules [21–22]. This kind of complex would behave like a macrocycle upon photoisomerization, arising the question whether it might be possible to predict such photoisomerizability and to relate it to the length of a ditopically bound guest molecule connecting the two metalloporphyrin units.

**Figure 1:**
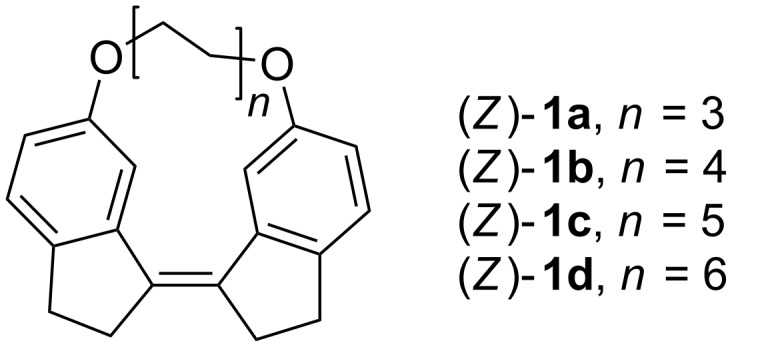
The investigated SS-macrocycles (*Z*)-**1a**–**d**.

To investigate the photoisomerization ability of the stiff stilbene as a macrocycle segment a series of model compounds were chosen (Figure 1). To keep the system as simple as possible the SS was attached to an aliphatic carbon chain via ether groups. Four different lengths of carbon chains were used, with distances between the terminal carbons of 6.4 Å (C6), 8.9 Å (C8), 11.4 Å (C10) and 13.9 Å (C12). The SS-macrocycles have been studied both experimentally and by computational techniques.

## Results and Discussion

### Synthesis

**Scheme 2:**
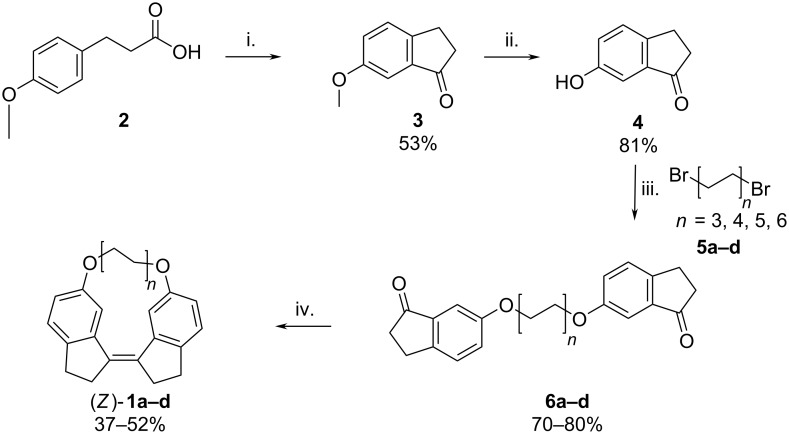
Synthetic route to SS-macrocycles. i. (1) Triflic acid (3 equiv), DCM (dry), Ar atmosphere, MW (110 °C, 1 h), (2) H_2_O (0 °C). ii. (1) AlCl_3_ (3 equiv), toluene (dry), Δ 1.5 h, (2) H_2_O. iii. (1) K_2_CO_3_ (4 equiv), TBAB (0.2 equiv), DMF (dry), Ar atmosphere, MW (150 °C, 15 min). iv. (1) TiCl_4_ (3 equiv), Zn powder (6 equiv), THF, Δ, 12 h.

The synthesis of the macrocycles was based on well-established reactions (Scheme 2). The indanone is formed by intramolecular Friedel–Crafts acylation of **2** under microwave radiation as reported by Oliverio et al. [23]. The second step is the demethylation of indanone methyl ether **3** by aluminium trichloride in toluene at reflux [24]. Two indanone units are then attached to an *n*-alkanediyl linker using a Williamson ether synthesis to yield the diethers **6a**–**d**. Finally, the stiff stilbene unit is formed by an intramolecular McMurry reaction resulting in **1a**–**d** [25–26]. The *Z*-isomer is formed in huge excess in these reactions and any trace amounts of *E*-isomer are removed during purification.

Compared to syntheses of other stiff stilbene macrocycles that typically start from indanone derivatives [15–16], our approach yields the target compounds in fewer steps from a simpler starting material, i.e., 3-(4'-methoxyphenyl)propionic acid (Scheme 2).

### Photoisomerization

**Scheme 3:**
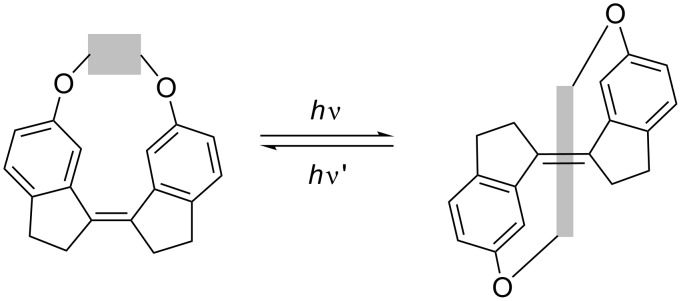
The photoisomerization of the stiff stilbene macrocycles, showing the stretching of the linker (grey box).

Photoisomerizing the (*Z*)-**1a**–**d** to the (*E*)-**1a**–**d** isomers requires to stretch the linker. The isomerization was achieved by irradiation of a degassed solution of (*Z*)-**1a**–**d** in chloroform or deuterated chloroform using either a 280 or 300 nm filter (Scheme 3). The conversion was followed by UV–vis or ^1^H NMR spectroscopy. Compounds were irradiated until an increase in isomerization yield could no longer be observed (see Supporting Information File 1 for details).

**Scheme 4:**
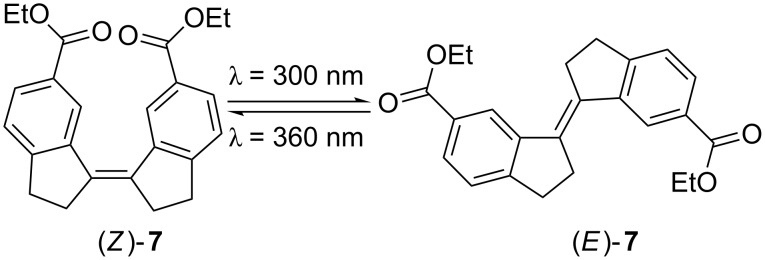
Noncyclic stiff stilbene diester **7** used as reference in the photoisomerization study.

To set the results of this photoisomerization into perspective a noncyclic stiff stilbene was used as a reference (Scheme 4). The photodynamic properties of this compound have been reported previously [27].

**Figure 2:**
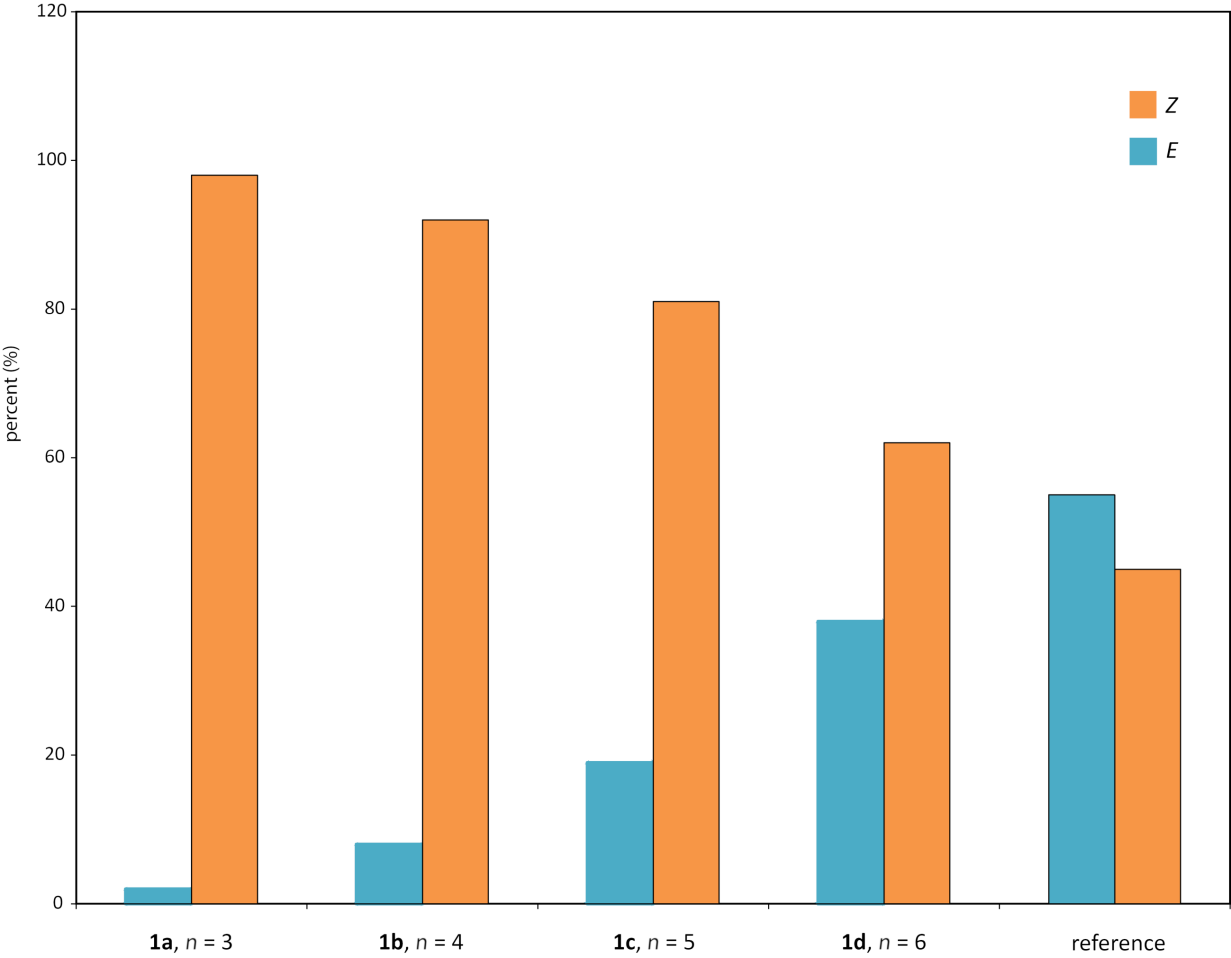
The photoisomerization of the SS-macrocycles shows a clear correlation between the *Z*/*E* ratio in the photostable mixture and the linker length. The non-cyclic SS-diester **7** is included as a reference.

The *E-* and *Z-*isomers give distinctively separated chemical shifts for the CH_2_ protons next to the double bond. This makes the determination of the *Z*/*E* ratio straightforward. The composition of the photostable mixtures as compared to the noncyclic reference is presented in Figure 2. As the linker chain gets shorter the *E*-isomer becomes less favored. What is particularly interesting is that even with the longest chain of twelve carbons a significantly lower amount of *E*-isomer as compared to the reference is obtained. Clearly even a loose linking chain has a considerable effect on the system.

### Computations

#### Relative energies of *E-* and *Z*-isomers

**Figure 3:**
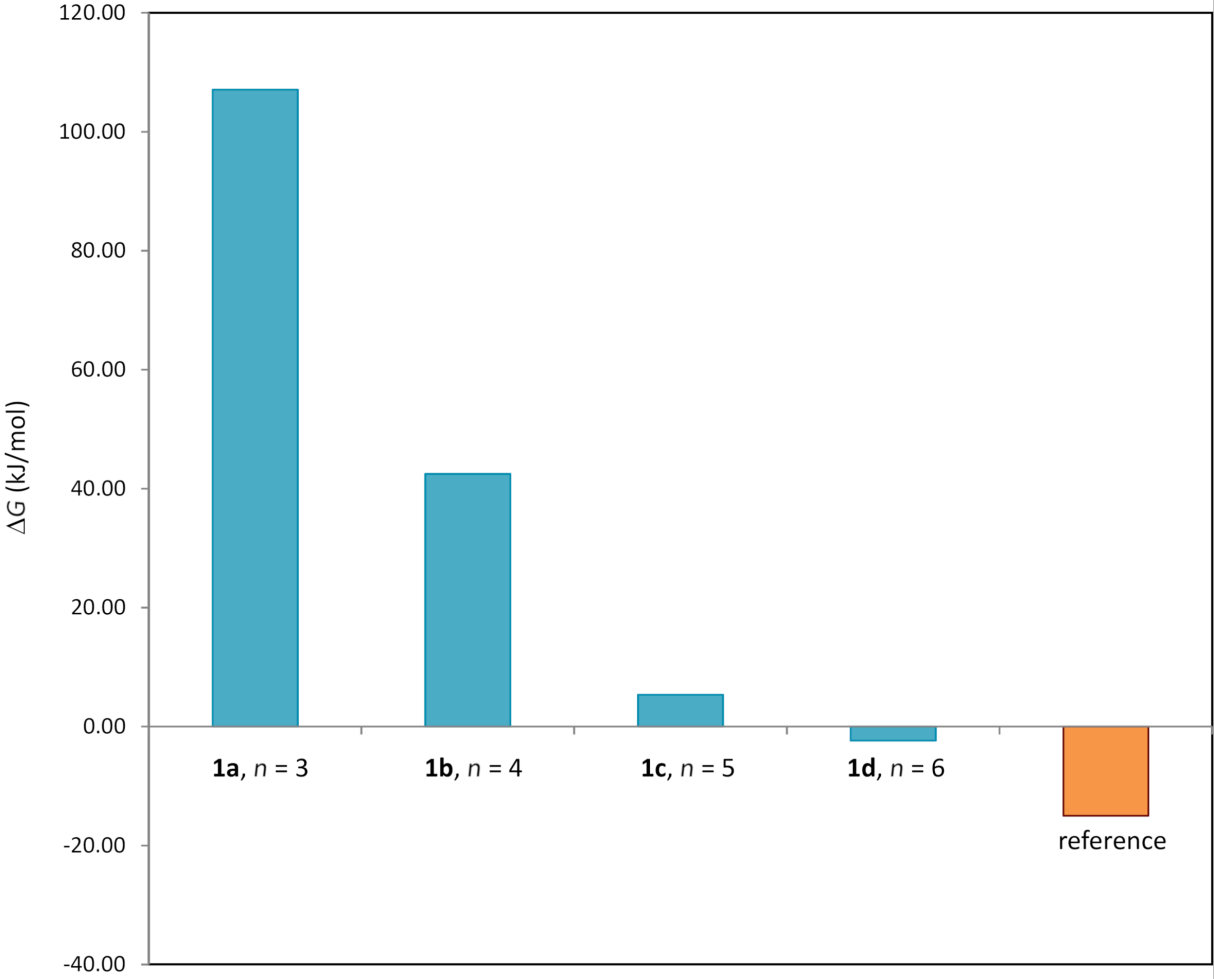
Gibbs free energy differences (Δ*G*) between *Z*- and *E*-isomers of **1a**–**d** and of the reference compound **7** calculated using B3LYP. The results show a pronounced effect of linker length on the energy difference between *Z*- and *E*-isomers.

The Gibbs free energies of (*Z*)-**1a**–**d** and (*E*)-**1a**–**d** were calculated at the DFT level using the B3LYP functional with the 6-31G(d,p) basis set and SCRF-SMD solvent model (chloroform) [28–37]. The photoisomerization of stiff stilbenes involves a complex potential energy surface with several excited species in equilibria, eventually reaching the *cis* or *trans* ground state [2]. Macroscopic parameters such as extinction coefficients and quantum yields also affect the composition of the photostationary state. Ground state energies might therefore not be directly related to the isomerization reaction without investigation of the exited state potential energy surface. However, the difference in Gibbs free energy (Δ*G*, Figure 3) between the *E*- and *Z*-isomers shows a trend reminiscent of the experimental photoisomerization results (Figure 2), i.e., shorter chain lengths result in larger Δ*G* as well as in larger *Z:E* ratios.

#### Ring strain

**Figure 4:**
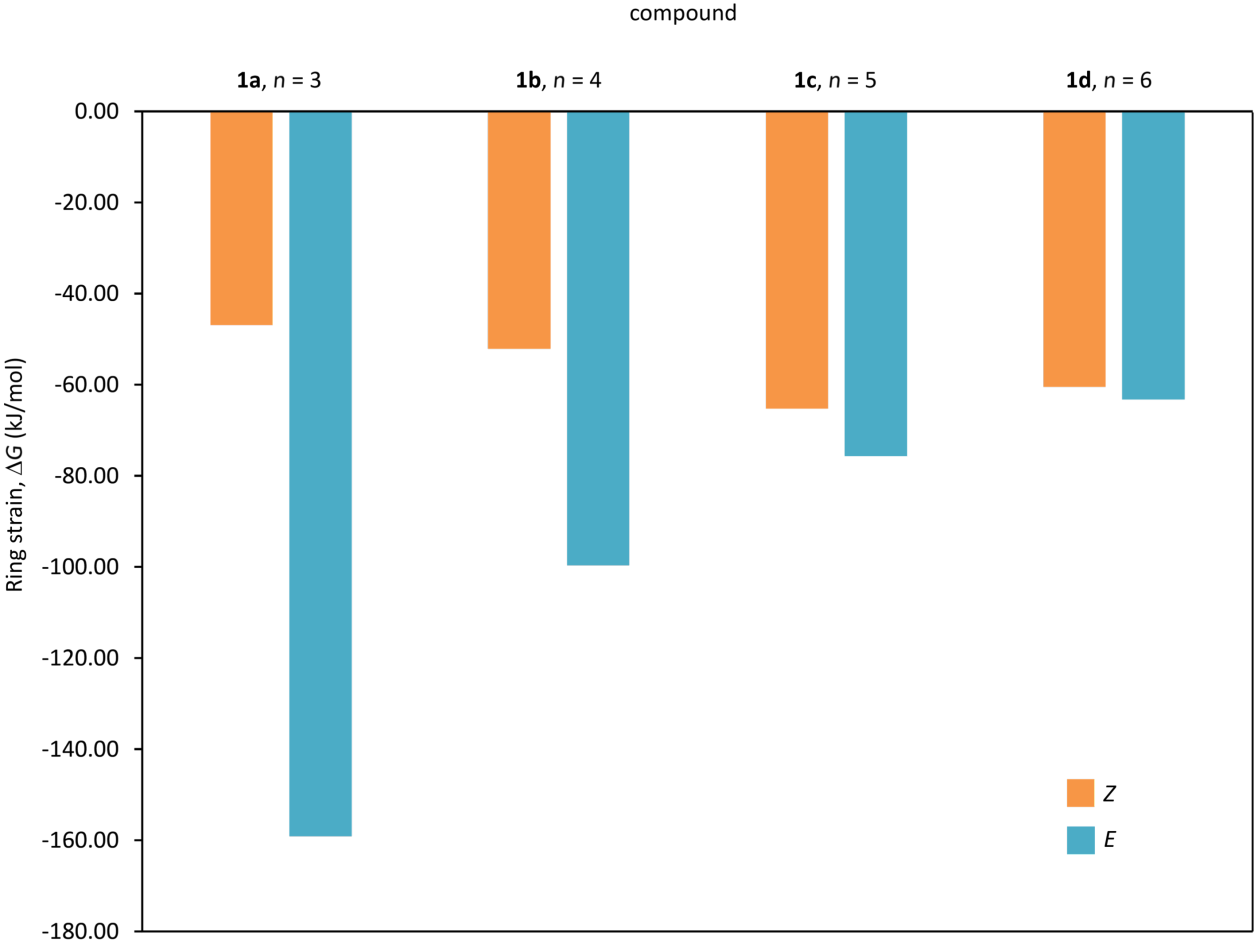
Ring strain for *E* and *Z*-isomers of **1a**–**d** expressed as the Gibbs free energy difference to an acyclic analogue, using an isodesmic reaction (Figure S47, Supporting Information File 1).

The ring strain energies of compounds (*Z*)-**1a**–**d** and (*E*)-**1a**–**d** were calculated for an isodesmic reaction [38] transforming the cyclic diethers into noncyclic diethers (Supporting Information File 1) and the results are visualized in Figure 4.

**Figure 5:**
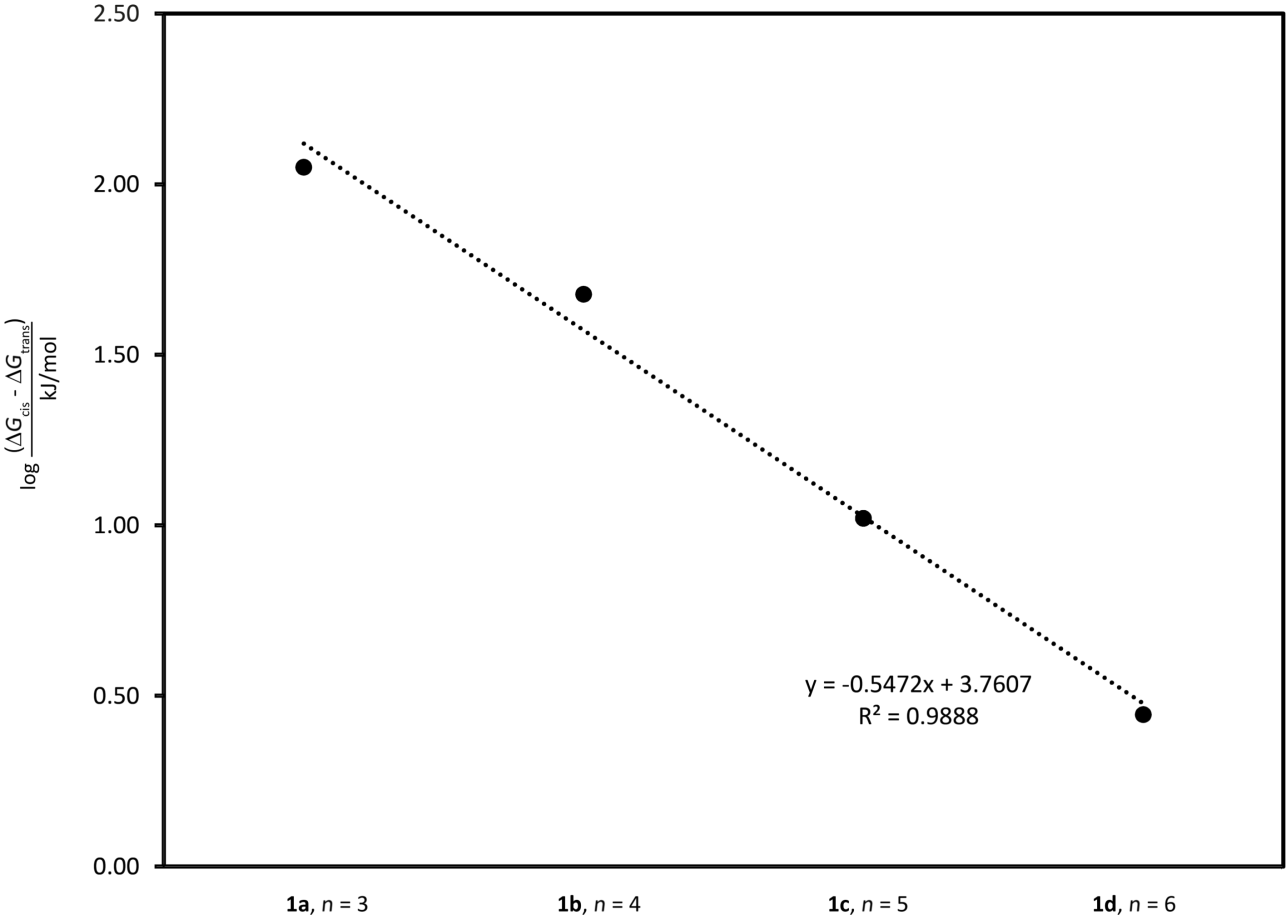
The differences in ring strain between the *E-* and *Z-*isomers show an exponential correlation to the linker length.

For (*E*)-**1a**–**d** the ring strain decreases with increased linker length. For the less strained (*Z*)-**1a**–**d** the ring strain increases slightly with increased linker length and for the longer linkers (**1c**,**d**) the ring strains of the *E-* and *Z*-isomers are similar. The differences in ring strain between the *E-* and *Z*-isomers show an exponential correlation to the linker length (Figure 5).

#### Conformational analysis

**Figure 6:**
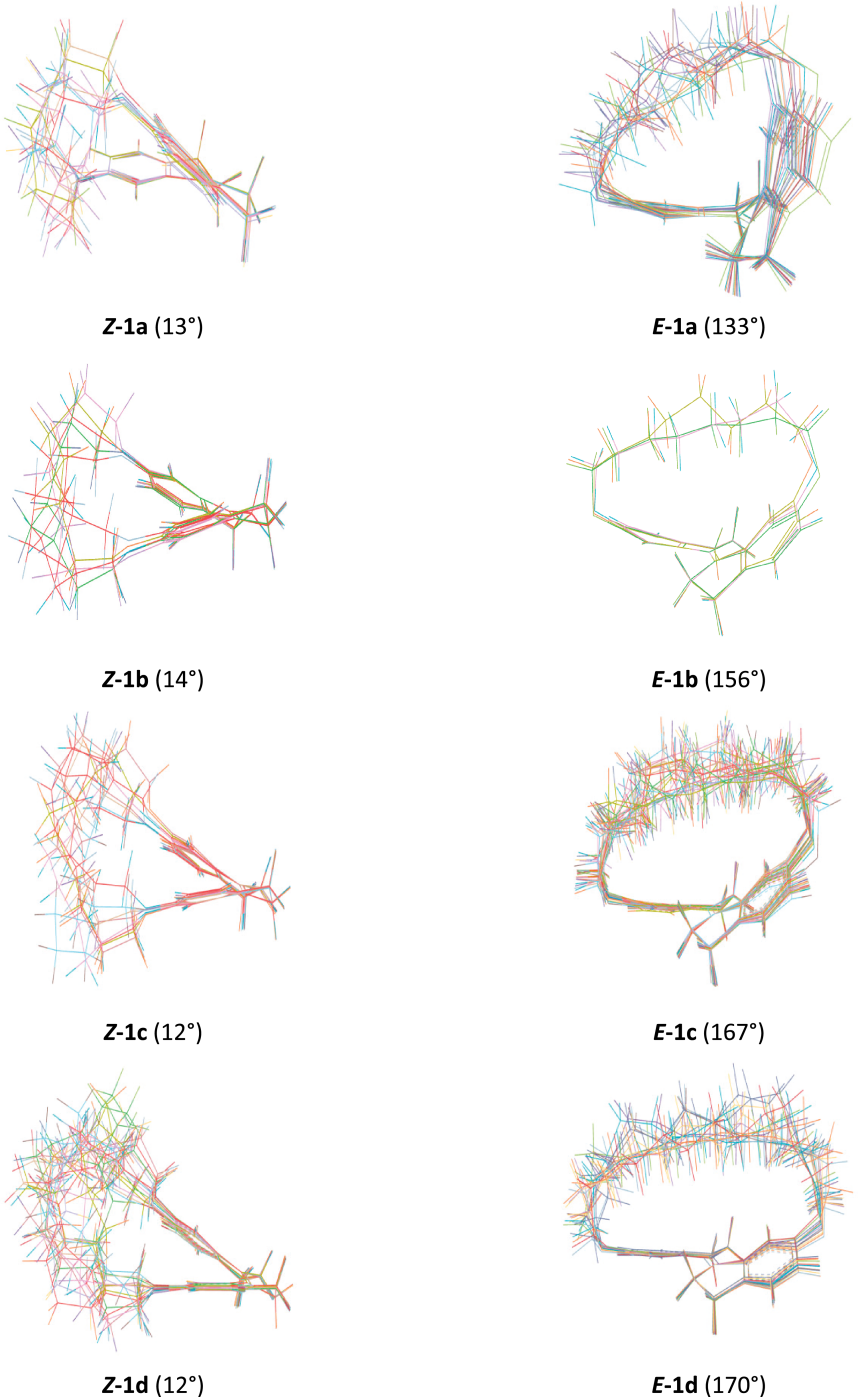
Conformer ensembles for the macrocyclic stiff stilbene diethers **1a**–**d**. Dihedral angles between the two aromatic rings are given in parentheses.

To obtain further information regarding the reason for the observed photoisomerization properties of the macrocyclic stiff stilbene diethers, a conformational analysis was undertaken (Figure 6).

According to X-ray crystallography, in compound (*E*)*-***7** (Scheme 4) the aromatic rings of the two indane units are in the same plane (dihedral angle 180°), whereas in (*Z*)-**7** this angle is 9.1° [21]. In the macrocyclic diethers **1a**–**d**, all *Z*-isomers have a dihedral angle of 12–14°, roughly similar to the one in the crystal structure of (*Z*)-**7**. The deviation of this angle from 0° is due to steric interaction between two aromatic protons in position 4 (Figure 9). In the *E*-isomers, an increasing distortion of the stiff stilbene segment with decreasing ring size is indicated by the substantial deviation of the dihedral angle from 180°. Furthermore, the alkyl chains adopt more similar conformations in the *E*-isomers with stretched alkyl chains. In the *Z*-isomers, the alkyl chains adopt a larger variety of conformations. This will add an entropy penalty for the *E*-isomers.

#### Interatomic distances from NOE buildup rates

**Figure 7:**
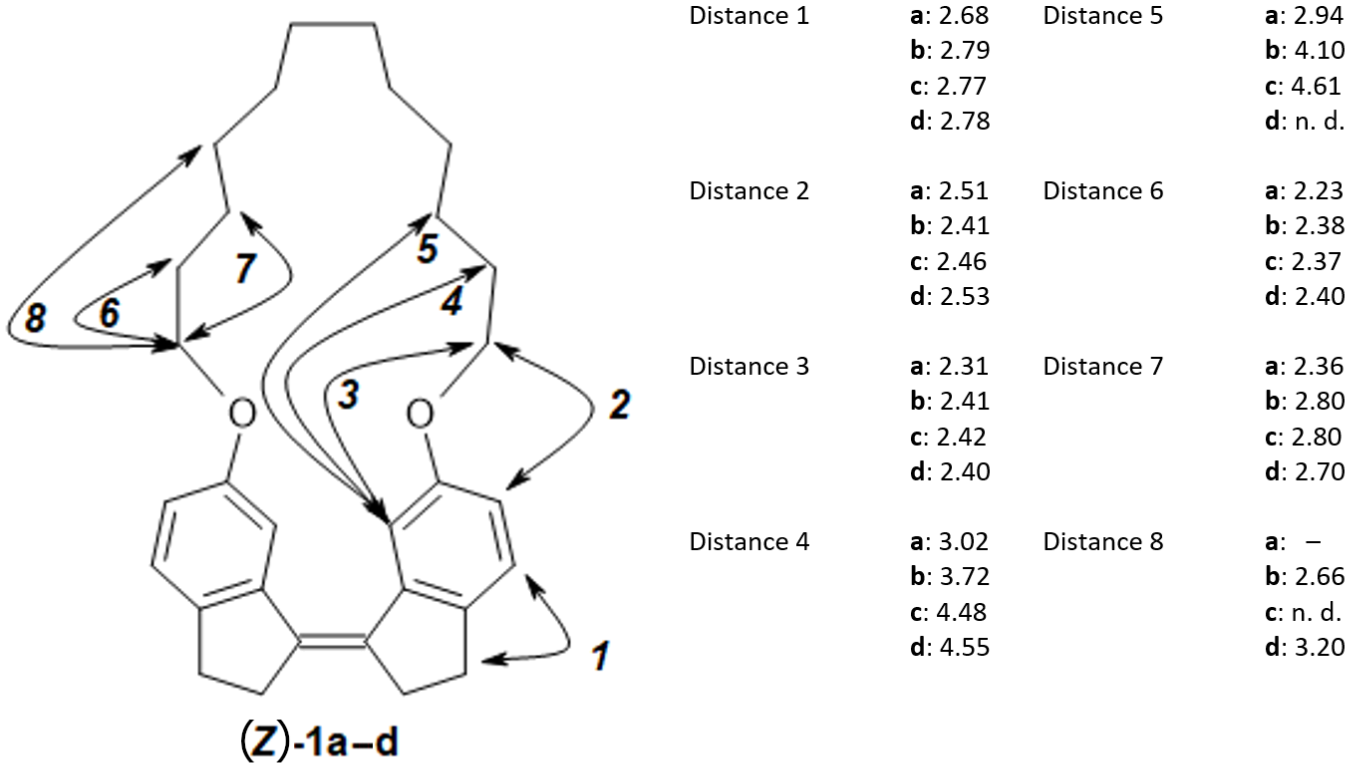
Distances derived from NOE buildup experiments. Distances between pairs of protons or groups of protons attached to the indicated carbons are designated as distance 1 through 8. n.d.: NOE cross peak not detectable. – : distance does not exist.

Interatomic distances, derived from NOE buildup rates, are summarized in Figure 7. Signal overlap prevented an analysis accounting for the presence of an ensemble of conformers such as NAMFIS [39–40]. For example, each CH_2_ signal is generated by four CH_2_ protons which are chemically equivalent in the averaged chemical structure (≈ the 2D molecular structure) but not in individual conformers. They cannot be distinguished on the NMR timescale. Therefore, the calculated distances *r*_ave_, being averages with contributions from all conformers, are biased for shorter distances, i.e., *r*_ave_ = ⟨1/*r*^6^⟩ instead of *r*_ave_ = 1/⟨*r*^6^⟩ [41]. However, they still should allow a comparison between the different compounds (*Z*)-**1a–d**. Thus, increased conformational flexibility is indicated by increasing distances from (*Z*)-**1a** to **1d** for methylene protons further along the chain, such as distance 4 and distance 5 (Figure 7). An exception is the slight decrease of distance 7 when comparing (*Z*)-**1c** to (*Z*)-**1d**. This might be due to larger mobility of the alkyl chain.

## Conclusion

A series of novel stiff stilbene macrocycles has been synthesised and used to investigate the effect of the ring size on the photoisomerization of the stiff stilbene unit. Both experimental photoisomerization and DFT calculations show that the strain of the linking chain affects the isomerization even for the longest chains. As stiff stilbene is gaining popularity as a unit in molecular machines and photodynamic systems a clear understanding of the effect of cyclisation on the photoisomerization is of general interest.

## Experimental

Starting materials, solvents and reagents were commercially available and used without further purification except dichloromethane (DCM), ethyl acetate, pentane, tetrahydrofuran (THF) and toluene that were distilled before use. *N*,*N*-Dimethylformamide (DMF) was used as supplied (biotech. grade, ≥99.9%). Unless stated differently, all reactions were carried out under atmospheric pressure and with argon atmosphere.

Microwave (MW) heating was carried out in a Biotage+ Initiator microwave using 10–20 mL Biotech MW vials, applying MW irradiation at 2.45 GHz, with a power setting up to 40 W and an average pressure of 4–5 bar when DCM was the solvent and 90 W/1 bar when the solvent was DMF. Analytical TLC was performed using Merck precoated silica gel 60 F254 plates, visualized with UV light and Hannessian's stain (5% ammonium molybdate, 1% cerium sulfate and 10% sulfuric acid in water). Flash chromatography (CC) was performed over Matrex silica gel (60 Å, 35–70 µm) on a regular column or on a Grace Reveleris X2 Flash chromatography system.

^1^H and ^13^C NMR spectra were recorded on Varian Mercury Plus (^1^H at 300.03 MHz), Agilent 400-MR DD2 (^1^H at 399.98 MHz, ^13^C at 100.58 MHz), Varian Unity Inova (^1^H at 499.94 MHz) and Bruker Avance Neo (^1^H at 500.15 MHz, ^13^C at 125.78 MHz) spectrometers at 25 °C. Chemical shifts (δ) are reported in ppm referenced indirectly to tetramethylsilane via the residual solvent signal (CDCl_3_, ^1^H at 7.26 and ^13^C at 77.0 ppm). Coupling constants are given in Hz. Signal assignments were derived from ^1^H-gCOSY [42–43], gTOCSY [44], gHSQC [45], gHMBC [46], and gNOESY [47] spectra.

Experimental conditions for NOE buildup experiments: gradient enhanced NOESY spectra were obtained for nondegassed solutions (16–46 mM) in CDCl_3_ at 25 °C, 400 MHz, mixing times = 0.1, 0.2, 0.3, 0.5, 0.7 s. The distance between aromatic ortho protons (H-6 and H-7 in Figure 9) was used as reference distance *r*_ref_ at 2.51 Å. Volume integrals for NOESY diagonal and cross peaks were measured for mixing times during the linear NOE buildup phase. For each signal pair A/B with a NOESY cross peak an average cross peak volume was calculated from measured volume integrals as:

[1] $$\text{average volume = }\sqrt{\frac{{\text{(cross peak}}_{\text{AB}}\times {\text{cross peak}}_{\text{BA}})}{\text{(diagonal peak A}\times \text{diagonal peak B})}}$$

The slope σ from the plot of average volume vs mixing time was determined and from it the distance *r*_AB_ calculated assuming *r*_AB =_ *r*_ref_(σ_ref_/σ_AB_)^1/6^.

Mass spectra were obtained on an Advion Expression-L CMS with APCI+ interface. High-resolution mass spectra were obtained on a Thermo Scientific Q-Exactive instrument in APCI positive mode. UV–vis spectra were recorded on a Shimadzu UV-1650PC spectrophotometer using 10 mm quartz cuvettes. Photoisomerizations were performed using an Oriel 1000 W Xe ARC light source equipped with a band pass filter 10BPF10-300 or 10BPF10-280 (Newport).

### Computational details

The DFT calculations on the stiff stilbene macrocycles were performed with the B3LYP functional as implemented in the Gaussian 16 program package [28–32]. The SCRF solvent model with the SMD variation was used with chloroform as solvent [33–36]. Geometries were optimized using the 6-31G(d,p) basis set [37]. Frequency calculations were performed at the same level to confirm that a minimum had been reached and to extract free energy corrections, which were evaluated at 298.15 K. A stability analysis was performed to ensure that a stable wave-function was attained for all species.

Conformational analyses of the stiff stilbene macrocycles were calculated in MacroModel 9.9 with the OPLS3e force field, CHCl_3_ as solvent and dielectric constant 9.1 [48–49]. Redundant conformer elimination in MacroModel was used to reduce the number of conformations to 10–20 structures [50].

### Synthesis

#### Synthesis of 6-methoxyindan-1-one (**3**)

Compound **2** (2.523 g, 14.0 mmol) was dissolved in dry DCM (10 mL) in a flame-dried MW vial and cooled in ice-bath. TfOH (3.7 mL, 41.9 mmol) was added dropwise. The vial was sealed, the air was replaced by argon gas, and the reaction mixture was heated in the MW to 110 °C, 5 bar, for 1 h. The reaction mixture was poured on ice. The water phase was extracted three times with DCM (3 × 100 ml). The combined organic phases were dried over MgSO_4_ and the solvent was removed by rotary evaporation. The crude product was purified by CC (pentane/EtOAc 1:0 to 1:4). The solvent was evaporated, giving a light yellow solid, 1.204 g, 53% yield. ^1^H NMR (CDCl_3_, 500 MHz) δ 7.37 (m, 1H, Ar-H), 7.20 (m, 1H, Ar-H), 7.18 (m, 1H, Ar-H), 3.84 (s, 3H, OCH_3_), 3.07 (m, 2H, CH_2_CH_2_CO), 2.72 (m, 2H, CH_2_CO); ^13^C NMR (CDCl_3_, 100.6 MHz) δ 207.0 (CO), 159.4 (C-OCH_3_), 148.0 (C, Ar), 138.2 (C, Ar), 127.3 (CH, Ar), 124.0 (CH, Ar), 104.9 (CH, Ar), 55.6 (OCH_3_), 37.0 (CH_2_CO), 25.1 (CH_2_CH_2_CO); APCI–MS *m*/*z*: [M + H]^+^ calcd for C_10_H_10_O_2_, 163; found, 163. Data in agreement with the literature [51].

#### Synthesis of 6-hydroxyindan-1-one (**4**)

Compound **3** (1.367 g, 8.4 mmol) and AlCl_3_ (3.483 g, 26.1 mmol) were dissolved in dry toluene (50 mL) and refluxed for 1.5 h. The reaction mixture was cooled to rt. H_2_O (70 mL) was added and the organic phase collected. The water phase was extracted three times with EtOAc (3 × 50 mL). The combined organic phases were washed with brine two times (2 × 75 mL) and dried over MgSO_4_. The solvent was removed by rotary evaporation. The orange crude product was purified by CC (pentane/EtOAc 1:0 to 1:4). The solvent was evaporated, giving a light orange solid, 1.103 g, 81% yield. ^1^H NMR (CDCl_3_, 500 MHz) δ 7.36 (d, *J* = 8.3 Hz, 1H, Ar-H), 7.22 (d, *J* = 2.4 Hz, 1H, Ar-H), 7.16 (dd, *J* = 2.4, 8.3 Hz, 1H, Ar-H), 5.67 (s, 1H, OH), 3.08 (m, 2H, CH_2_CH_2_CO), 2.73 (m, 2H, CH_2_CO); ^13^C NMR (CDCl_3_, 100.6 MHz) δ 207.4 (CO), 155.4 (C-OH), 147.8 (C, Ar), 138.3 (C, Ar), 127.6 (CH, Ar), 123.4 (CH, Ar), 108.7 (CH, Ar), 37.0 (CH_2_CO), 25.1 (CH_2_CH_2_CO); APCI–MS *m*/*z*: [M + H]^+^ calcd for C_9_H_8_O_2_, 149; found, 149. Data in agreement with the literature [52].

#### General procedure A: Williamson ether synthesis (assisted by MW)

Compound **4** (2 equiv), dibromoalkane **5** (1 equiv), TBAB (0.2 equiv) and K_2_CO_3_ (4 equiv) were dissolved in dry DMF (15 mL) in a flame-dried MW vial. The vial was sealed, put under argon and heated in the MW to 150 °C for 15 min (the reaction was followed by NMR). The reaction mixture was cooled to rt and poured on DCM (40 mL), filtered and washed with water four times (4 × 50 mL) and brine three times (3 × 50 mL). The organic phase was dried over MgSO_4_ and the solvent removed by rotary evaporation. The product was dried under high vacuum overnight.

**Figure 8:**
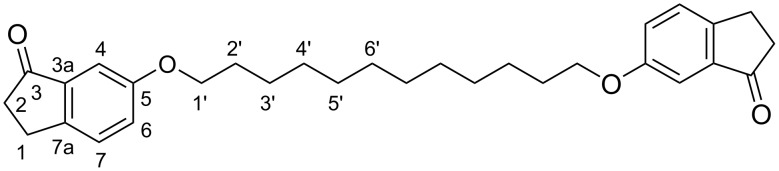
Numbering of carbons in compounds **6a**–**d**, showing **6d** as an example.

#### Synthesis of 6-[2-(3-oxoindan-5-yl)oxyhexyloxy]indan-1-one (**6a**)

The synthesis followed general procedure A with compound **4** (0.201 g, 1.4 mmol) and 1,6-dibromohexane (**5a**, 0.11 mL, 0.7 mmol) as starting materials, giving a brown solid which was sufficiently pure for subsequent steps, 0.176 g, 69% yield. ^1^H NMR (CDCl_3_, 500 MHz) δ 7.36 (m, 2H, H-7), 7.20–7.16 (m, 4H, H-4 H-6), 4.00 (t, *J* = 6.6 Hz, 4H, CH_2_-1’), 3.07 (m, 4H, CH_2_-1), 2.72 (m, 4H, CH_2_-2), 1.84 (m, 4H, CH_2_-2’), 1.54 (m, 4H, CH_2_-3’); ^13^C NMR (CDCl_3_, 100.6 MHz) δ 207.1 (C, C-3), 158.8 (C, C-5), 147.8 (C, C-3a), 138.2 (C, C-7a), 127.3 (CH, C-7), 124.4 (CH, C-6), 105.6 (CH, C-4), 68.2 (CH_2_, C-1’), 37.0 (CH_2_, C-2), 29.0 (CH_2_, C-2’), 25.8 (CH_2_, C-3’), 25.1 (CH_2_, C-1); APCI–MS *m*/*z*: [M + H]^+^ calcd for C_24_H_26_O_4_, 379; found, 379; UV–vis (CH_2_Cl_2_) λ_max_: 320, 249 nm.

#### Synthesis of 6-[2-(3-oxoindan-5-yl)oxyoctyloxy]indan-1-one (**6b**)

The synthesis followed general procedure A with compound **4** (0.115 g, 0.8 mmol) and 1,8-dibromooctane (**5b**, 0.07 mL, 0.4 mmol) as starting materials, giving an orange solid which was sufficiently pure for subsequent steps, 0.121 g, 78% yield. ^1^H NMR (CDCl_3_, 500 MHz) δ 7.36 (m, 2H, H-7), 7.20–7.16 (m, 4H, H-4 H-6), 3.98 (t, *J* = 6.6 Hz, 4H, CH_2_-1’), 3.06 (m, 4H, CH_2_-1), 2.71 (m, 4H, CH_2_-2), 1.80 (dt, *J* = 6.6, 14.8 Hz, 4H, CH_2_-2’), 1.47 (m, 4H, CH_2_-3’), 1.40 (m, 4H, CH_2_-4’); ^13^C NMR (CDCl_3_, 100.6 MHz) δ 207.1 (C, C-3), 158.8 (C, C-5), 147.8 (C, C-3a), 138.2 (C, C-7a), 127.3 (CH, C-7), 124.4 (CH, C-6), 105.6 (CH, C-4), 68.3 (CH_2_, C-1’), 37.0 (CH_2_, C-2), 29.2 (CH_2_, C-4’), 29.1 (CH_2_, C-2’), 25.9 (CH_2_, C-3’), 25.1 (CH_2_, C-1); APCI–MS *m*/*z*: [M + H]^+^ calcd for C_26_H_30_O_4_, 407; found, 407; HRMS (CI) *m*/*z*: [M + H]^+^ calcd for C_26_H_30_O_4_, 407.2217; found, 407.2217; UV–vis (CH_2_Cl_2_) λ_max_: 320, 249 nm.

#### Synthesis of 6-[2-(3-oxoindan-5-yl)oxydecyloxy]indan-1-one (**6c**)

The synthesis followed general procedure A with compound **4** (0.397 g, 2.7 mmol) and 1,10-dibromodecane **5c** (0.405 g, 1.3 mmol) as starting materials, giving a light brown solid which was sufficiently pure for subsequent steps, 0.471 g, 80% yield. ^1^H NMR (CDCl_3_, 500 MHz) δ 7.34 (m, 2H, H-7), 7.20–7.16 (m, 4H, H-4 H-6), 3.98 (t, *J* = 6.8 Hz, 4H, CH_2_-1’), 3.07 (m, 4H, CH_2_-1), 2.71 (m, 4H, CH_2_-2), 1.79 (dt, *J* = 6.8, 15.0 Hz, 4H, CH_2_-2’), 1.46 (m, 4H, CH_2_-3’), 1.40–1.30 (m, 8H, CH_2_-4’ CH_2_-5’); ^13^C NMR (CDCl_3_, 100.6 MHz) δ 207.1 (C, C-3), 158.9 (C, *C*-5), 147.8 (C, C-3a), 138.2 (C, C-7a), 127.3 (CH, C-7), 124.4 (CH, C-6), 105.6 (CH, C-4), 68.4 (CH_2_, C-1’), 37.0 (CH_2_, C-2), 29.4 (CH_2_, C-5’), 29.2 (CH_2_, C-4’), 29.1 (CH_2_, C-2’), 26.0 (CH_2_, C-3’), 25.1 (CH_2_, C-1); APCI–MS *m*/*z*: [M + H]^+^ calcd for C_28_H_34_O_4_, 435; found, 435. HRMS (CI) *m*/*z*: [M + H]^+^ calcd for C_28_H_34_O_4_, 435.2530; found: 435.2527; UV–vis (CH_2_Cl_2_) λ_max_: 320, 248 nm.

#### Synthesis of 6-[2-(3-oxoindan-5-yl)oxydodecyloxy]indan-1-one (**6d**)

The synthesis followed General procedure A with compound **4** (0.102 g, 0.7 mmol) and 1,12-dibromododecane **5d** (0.112 g, 3.5 × 10^−2^ mmol) as starting materials, giving a light brown solid which was sufficiently pure for subsequent steps, 0.112 g, 71% yield. ^1^H NMR (CDCl_3_, 500 MHz) δ 7.36 (m, 2H, H-7), 7.20–7.17 (m, 4H, H-4 H-6), 3.98 (t, *J* = 6.8 Hz, 4H, CH_2_-1’), 3.07 (m, 4H, CH_2_-1), 2.71 (m, 4H, CH_2_-2), 1.79 (dt, *J* = 6.8, 14.8 Hz, 4H, CH_2_-2’), 1.45 (m, 4H, CH_2_-3’), 1.39–1.27 (m, 12H, CH_2_-4’ CH_2_-5’ CH_2_-6’); ^13^C NMR (CDCl_3_, 100.6 MHz) δ 207.1 (C, C-3), 158.9 (C, C-5), 147.7 (C, C-3a), 138.2 (C, C-7a), 127.3 (CH, C-7), 124.4 (CH, C-6), 105.6 (CH, C-4), 68.4 (CH_2_, C-1’), 37.0 (CH_2_, C-2), 29.5 (CH_2_, 4C, C-5’ C-6’), 29.3 (CH_2_, C-4’), 29.1 (CH_2_, C-2’), 26.0 (CH_2_, C-3’), 25.1 (CH_2_, C-1); APCI–MS: *m*/*z*: [M + H]^+^ calcd for C_30_H_38_O_4_, 463; found, 463; HRMS (CI) *m*/*z*: [M + H]^+^ calcd for C_30_H_38_O_4_, 463.2843; found, 463.2836; UV–vis (CH_2_Cl_2_) λ_max_: 320, 248 nm.

#### General procedure B: McMurry coupling

Zinc powder previously grinded (12 equiv) was suspended in dry THF (30 mL). The suspension was cooled to 0 °C in an ice bath and TiCl_4_ (6 equiv) added over 10 minutes. The resulting slurry was refluxed for 1.5 h. A solution of compound **6** in dry THF (50–100 mL) was added over a 5–7 h period to the refluxing reaction mixture by syringe pump. The refluxing was continued for 40 min after the addition was complete. The reaction mixture was cooled to rt and poured on a saturated aqueous solution of NH_4_Cl. The water phase was extracted three times with DCM (3 × 100 mL). The combined organic phases were washed two times with brine (2 × 100 mL) then dried over MgSO_4_ and the solvent was removed by rotary evaporation. Unless stated differently, the obtained yellow oil was purified by CC (pentane/DCM 1:0 to 1:1). The obtained product was dried under high vacuum overnight.

**Figure 9:**
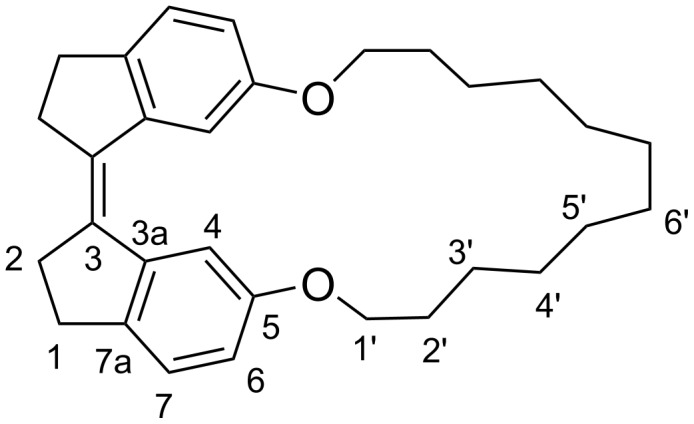
Numbering of carbons in compounds (*Z*)-**1a**–**d**, showing (*Z*)-**1d** as an example.

#### Synthesis of macrocyclic stiff stilbene diether (*Z*)-**1a**

The synthesis followed general procedure B with compound **6a** (0.279 g, 0.7 mmol) as starting material and gave the pure product as a light yellow solid, 0.093 g, 37% yield. ^1^H NMR (CDCl_3_, 500 MHz) δ 7.75 (d, *J* = 2.3 Hz, 2H, H-4), 7.19 (d, *J* = 8.0 Hz, 2H, H-7), 6.80 (dd, *J* = 2.3, 8.0 Hz, 2H, H-6), 4.07 (t, *J* = 6.5 Hz, 4H, CH_2_-1’), 2.94 (m, 4H, CH_2_-1), 2.82 (m, 4H, CH_2_-2), 1.80 (m, 4H, CH_2_-2’), 1.59 (m, 4H, CH_2_-3’); ^13^C NMR (CDCl_3_, 100.6 MHz) δ 157.6 (C, C-5), 141.6 (C, C-7a), 141.1 (C, C-3a), 135.2 (C, C-3), 125.5 (CH, C-7), 116.2 (CH, C-6), 111.9 (CH, C-4), 69.7 (CH_2_, C-1’), 35.0 (CH_2_, C-2), 30.0 (CH_2_, C-1), 28.8 (CH_2_, C-2’), 24.4 (CH_2_, C-3’); APCI–MS *m*/*z*: [M + H]^+^ calcd for C_24_H_26_O_2_, 347; found, 347; HRMS (CI) *m*/*z*: [M + H]^+^ calcd for C_24_H_26_O_2_, 347.2006; found, 347.1996; UV–vis (CH_2_Cl_2_) λ_max_: 350, 298, 253 nm.

#### Synthesis of macrocyclic stiff stilbene diether (*Z*)-**1b**

The synthesis followed general procedure B with compound **6b** (0.105 g, 0.3 mmol) as starting material and gave the pure product as a light yellow solid, 0.038 g, 39% yield. ^1^H NMR (CDCl_3_, 500 MHz) δ 7.69 (d, *J* = 2.5 Hz, 2H, H-4), 7.18 (d, *J* = 8.2 Hz, 2H, H-7), 6.74 (dd, *J* = 2.5, 8.2 Hz, 2H, H-6), 3.97 (t, *J* = 6.1 Hz, 4H, CH_2_-1’), 2.93 (m, 4H, CH_2_-1), 2.82 (m, 4H, CH_2_-2), 1.82 (dt, *J* = 6.1, 12.8 Hz, 4H, CH_2_-2’), 1.56 (m, 4H, CH_2_-3’), 1.45 (m, 4H, CH_2_-4’); ^13^C NMR (CDCl_3_, 100.6 MHz) δ 157.6 (C, C-5), 141.6 (C, C-7a), 140.5 (C, C-3a), 135.4 (C, C-3), 125.4 (CH, C-7), 113.9 (CH, C-6), 110.0 (CH, C-4), 68.1 (CH_2_, C-1’), 35.4 (CH_2_, C-2), 29.8 (CH_2_, C-1), 28.1 (CH_2_, C-2’), 27.6 (CH_2_, C-4’), 25.3 (CH_2_, C-3’); APCI–MS *m*/*z*: [M + H]^+^ calcd for C_26_H_30_O_2_, 375; found, 375; HRMS (CI) *m*/*z*: [M + H]^+^ calcd for C_26_H_30_O_2_, 375.2319; found, 375.2311; UV–vis (CH_2_Cl_2_) λ_max_: 361, 349, 300, 253 nm.

#### Synthesis of macrocyclic stiff stilbene diether (*Z*)-**1c**

The synthesis followed general procedure B with compound **6c** (0.350 g, 0.8 mmol) as starting material and gave the pure product as a light yellow solid, 0.171g, 53% yield. ^1^H NMR (CDCl_3_, 500 MHz) δ 7.66 (d, *J* = 2.4 Hz, 2H, H-4), 7.19 (d, *J* = 8.3 Hz, 2H, H-7), 6.75 (dd, *J* = 2.4, 8.3 Hz, 2H, H-6), 3.92 (t, *J* = 5.9 Hz, 4H, C*H_2_*-1’), 2.93 (m, 4H, CH_2_-1), 2.82 (m, 4H, CH_2_-2), 1.79 (dt, *J* = 5.9, 12.6 Hz, 4H, CH_2_-2’), 1.55 (dt, *J* = 5.9, 12.6 Hz, 4H, CH_2_-3’), 1.45–1.37 (m, 8H, C*H_2_*-4’ C*H_2_*-5’); ^13^C NMR (CDCl_3_, 100.6 MHz) δ 157.7 (C, C-5), 141.7 (C, C-7a), 140.4 (C, C-3a), 135.5 (C, C-3), 125.4 (CH, C-7), 113.6 (CH, C-6), 109.5 (CH, C-4), 67.1 (CH_2_, C-1’), 35.6 (CH_2_, C-2), 29.8 (CH_2_, C-1), 28.4 (CH_2_, C-2’), 26.9 (CH_2_, C-4’), 26.4 (CH_2_, C-5’), 24.8 (CH_2_, C-3’); APCI–MS *m*/*z*: [M + H]^+^ calcd for C_28_H_34_O_2_, 403; found, 403; HRMS (CI) *m*/*z*: [M + H]^+^ calcd for C_28_H_34_O_2_, 403.2632; found: 403.2624; UV–vis (CH_2_Cl_2_) λ_max_: 361, 349, 301, 252 nm.

#### Synthesis of macrocyclic stiff stilbene diether (*Z*)-**1d**

The synthesis followed general procedure B with compound **6d** (0.312 g, 0.7 mmol) as starting material and gave the pure product as a light yellow solid, 0.152 g, 52% yield. ^1^H NMR (CDCl_3_, 500 MHz) δ 7.64 (d, *J* = 2.4 Hz, 2H, H-4), 7.19 (d, *J* = 8.3 Hz, 2H, H-7), 6.76 (dd, *J* = 2.4, 8.3 Hz, 2H, H-6), 3.91 (t, *J* = 6.3 Hz, 4H, CH_2_-1’), 2.93 (m, 4H, CH_2_-1), 2.82 (m, 4H, CH_2_-2), 1.76 (dt, *J* = 6.3, 15.0 Hz, 4H, CH_2_-2’), 1.49 (m, 4H, CH_2_-3’), 1.44–1.26 (m, 12H, CH_2_-4’ CH_2_-5’ CH_2_-6’); ^13^C NMR (CDCl_3_, 100.6 MHz) δ 157.8 (C, C-5), 141.6 (C, C-7a), 140.5 (C, C-3a), 135.4 (C, C-3), 125.4 (CH, C-7), 114.1 (CH, C-6), 109.3 (CH, C-4), 68.4 (CH_2_, C-1’), 35.5 (CH_2_, C-2), 29.8 (CH_2_, C-1), 29.6 (CH_2_, C-2’), 27.4 (CH_2_, C-4’), 27.1 (CH_2_, C-5’), 26.2 (CH_2_, C-6’), 25.1 (CH_2_, C-3’); APCI–MS *m*/*z*: [M + H]^+^ calcd for C_30_H_38_O_2_, 431; found, 431; HRMS (CI) *m*/*z*: [M + H]^+^ calcd for C_30_H_38_O_2_, 431.2945; found, 431.2928; UV–vis (CH_2_Cl_2_) λ_max_: 359, 349, 298, 252 nm.

#### Photoisomerizations (followed by NMR spectroscopy)

CDCl_3_ solutions of products (*Z*)-**1d** and stiff stilbene were irradiated after degassing by argon bubbling for 15 min. As reaction vessels, 5 mm NMR tubes, type 5Hp, 178 mm were used. The course of isomerization was assessed by ^1^H NMR spectroscopy.

#### Photoisomerizations (followed by UV–vis spectroscopy)

CHCl_3_ solutions of products (*Z*)-**1d** and stiff stilbene were irradiated after degassing by argon bubbling for 15 min. As reaction vessels, 10 mm quartz UV–vis cuvettes were used. The course of isomerization was assessed by UV–vis spectroscopy.

## Supporting Information

File 1 Experimental and theoretical data.
