# Supplementary material for: Effect of ring rize on photoisomerization properties of stiff stilbene macrocycles
Source: Beilstein J Org Chem. 2019 Oct 11;15:2408–18. doi: 10.3762/bjoc.15.233 (PMC6808211; doi:10.3762/bjoc.15.233)
Supplement: File 1 — Experimental and theoretical data. [file Beilstein_J_Org_Chem-15-2408-s001.pdf]

BEILSTEIN JOURNAL OF ORGANIC CHEMISTRY

Supporting Information

for

Effect of ring rize on photoisomerization properties of stiff stilbene macrocycles

Sandra Olsson, Óscar Benito Pérez, Magnus Blom and Adolf Gogoll

Beilstein J. Org. Chem. **2019,** *15,* 2408–2418. doi:10.3762/bjoc.15.233

Experimental and theoretical data

License and Terms: This is a supporting information file under the terms of the Creative Commons Attribution License (http://creativecommons.org/licenses/by/4.0). Please note that the reuse, redistribution and reproduction in particular requires that the authors and source are credited.
The license is subject to the *Beilstein Journal of Organic Chemistry* terms and conditions: (https://www.beilstein-journals.org/bjoc)

Table of Contents

1. NMR spectra S1
2. UV–vis spectra S15
3. Conformational analysis S21
4. NOE buildup S28
5. DFT calculations S32
   5.1 Energies S32
   5.2 Ring strain S32
   5.3 Optimized geometries S33

1. NMR spectra

^{1}H and ^{13}C NMR spectra of synthesized compounds and ^{1}H spectra of photoisomerization studies.

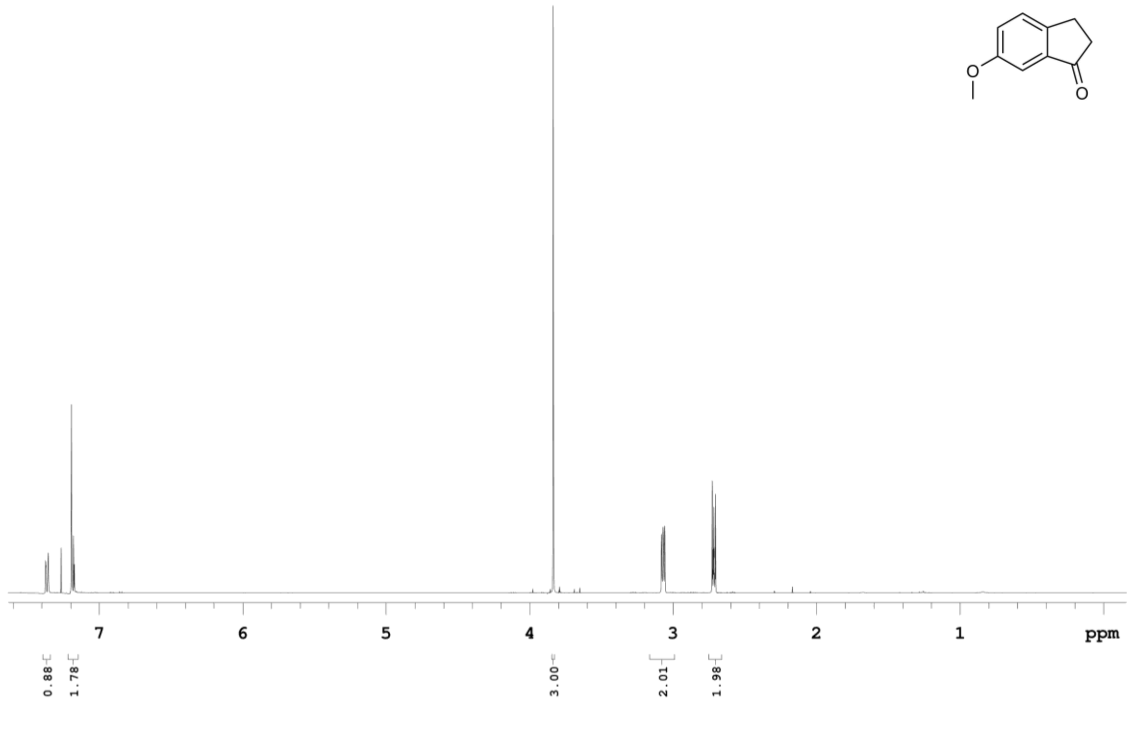

*Figure S1. ^{1}H NMR spectrum of compound **3**.*

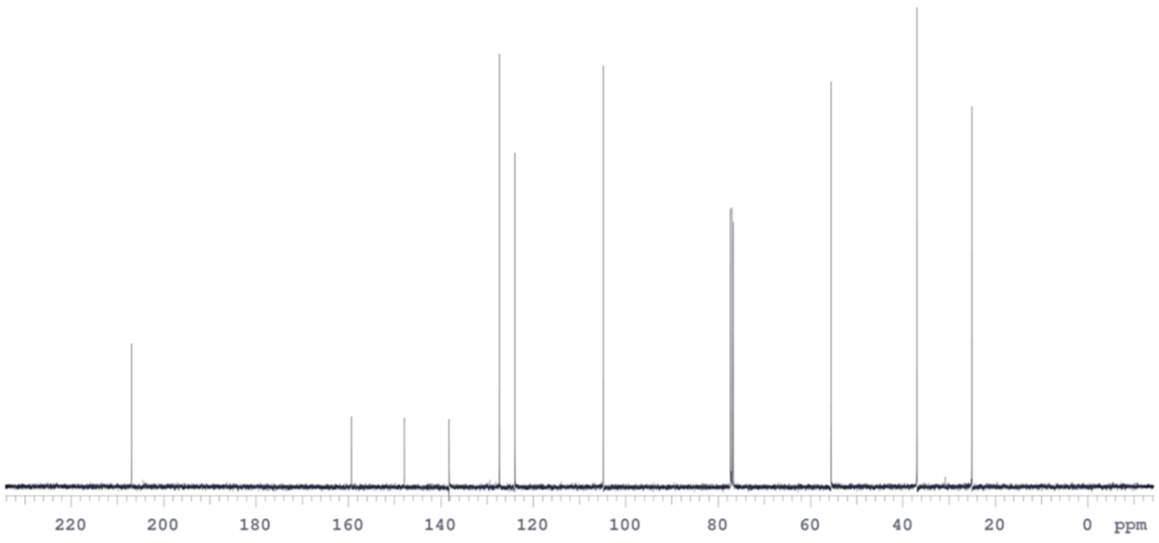

*Figure S2. ^{13}C NMR spectrum of compound **3**.*

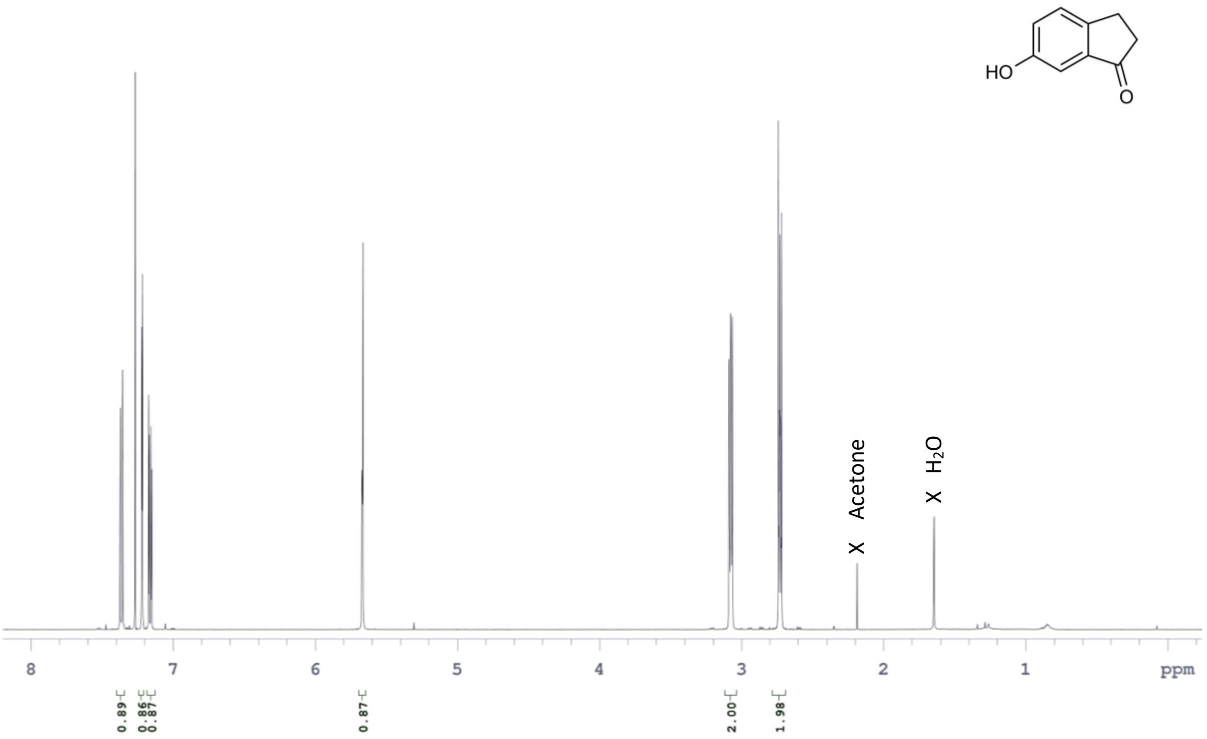

*Figure S3. ^{1}H NMR spectrum of compound **4**.*

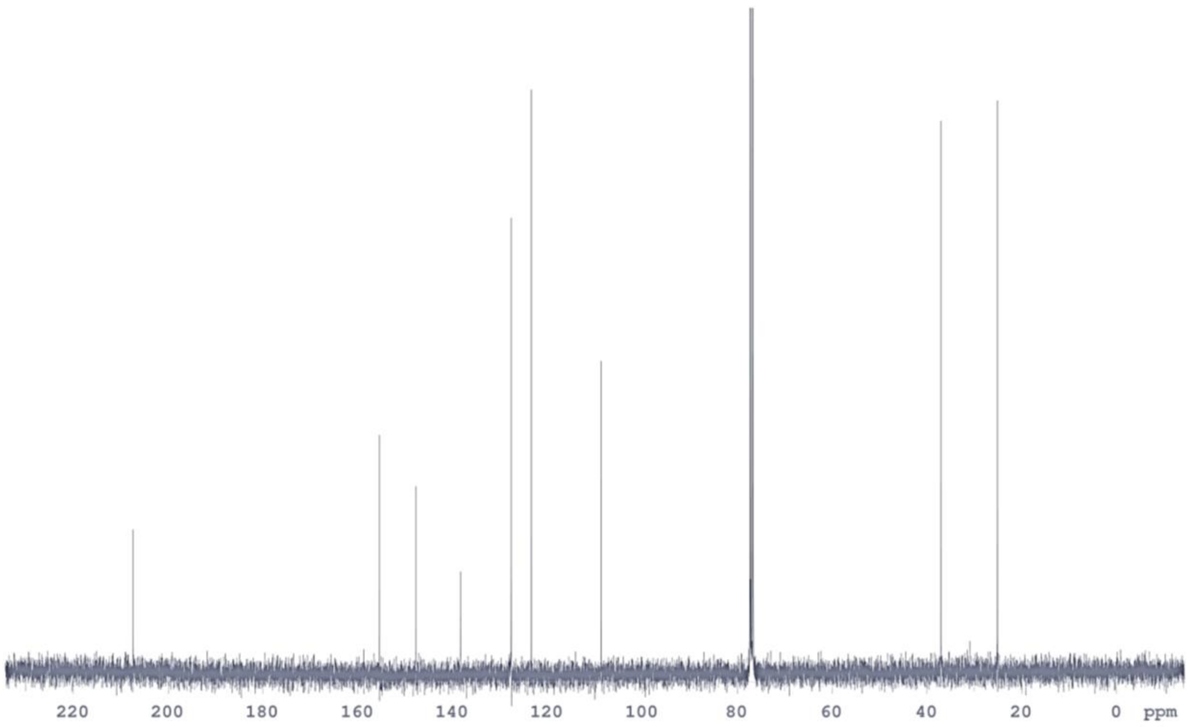

Figure S4. ^{13}*C NMR spectrum of compound* ***4***.

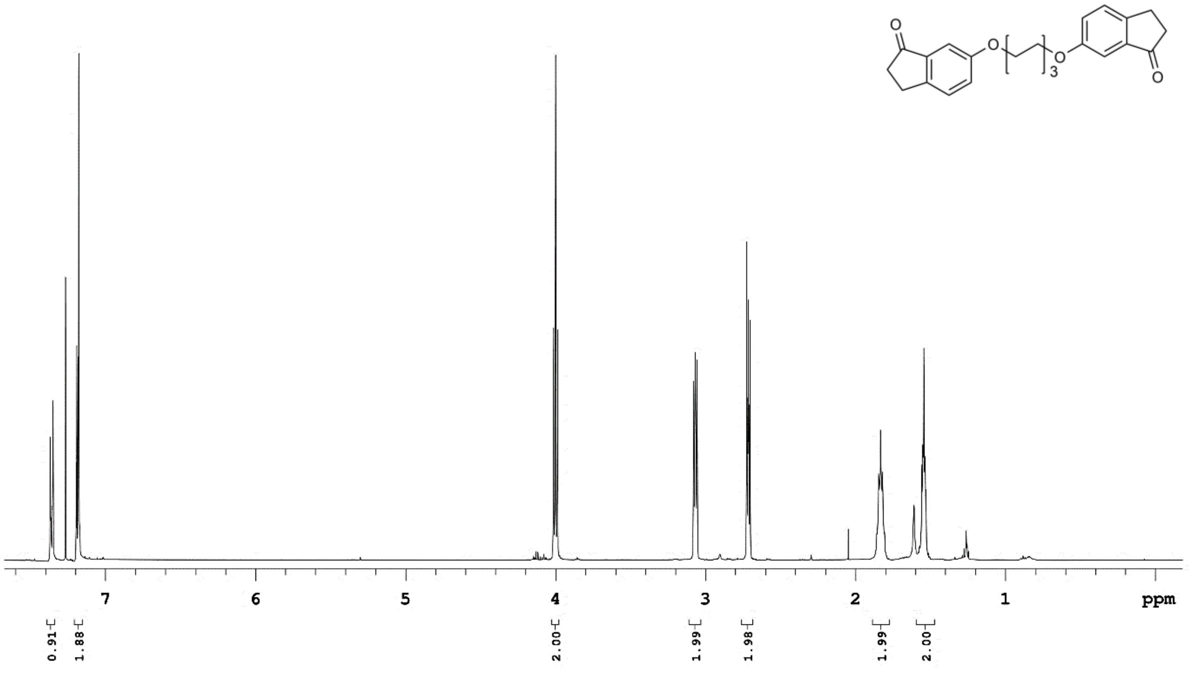

Figure S5. ^{1}H *NMR spectrum of compound* ***6a***.

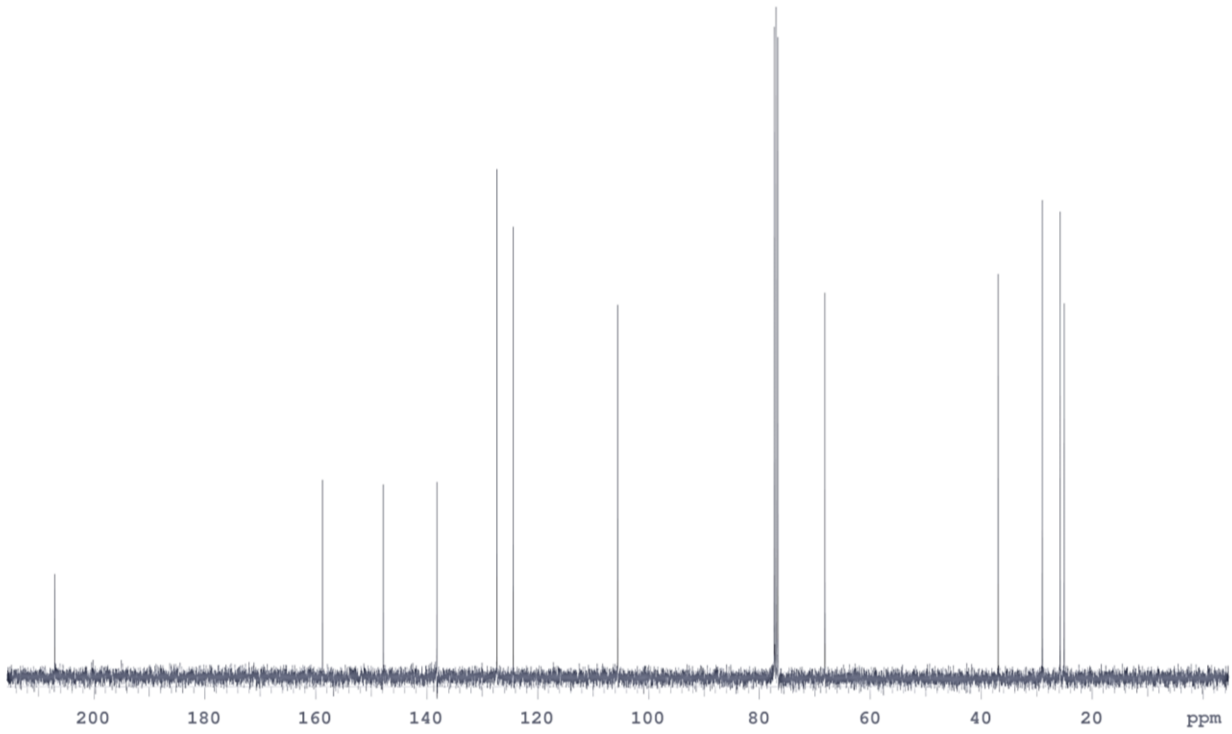

*Figure S6. ^{13}C NMR spectrum of compound **6a**.*

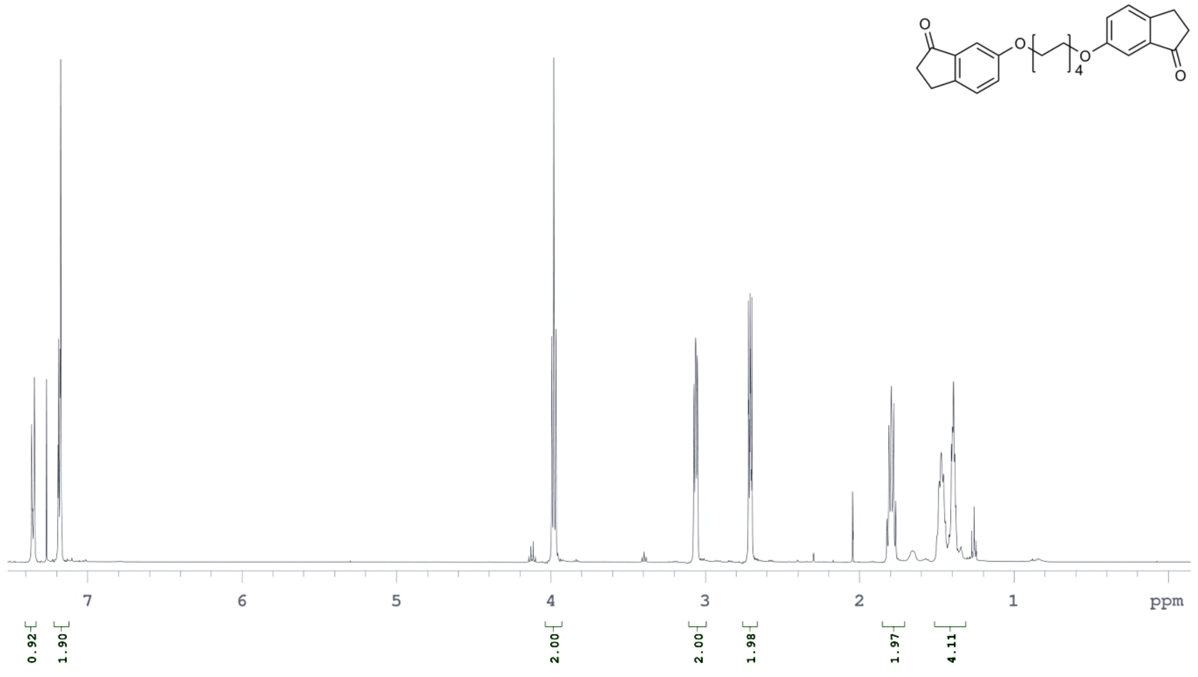

*Figure S7. ^{1}H NMR spectrum of compound **6b**.*

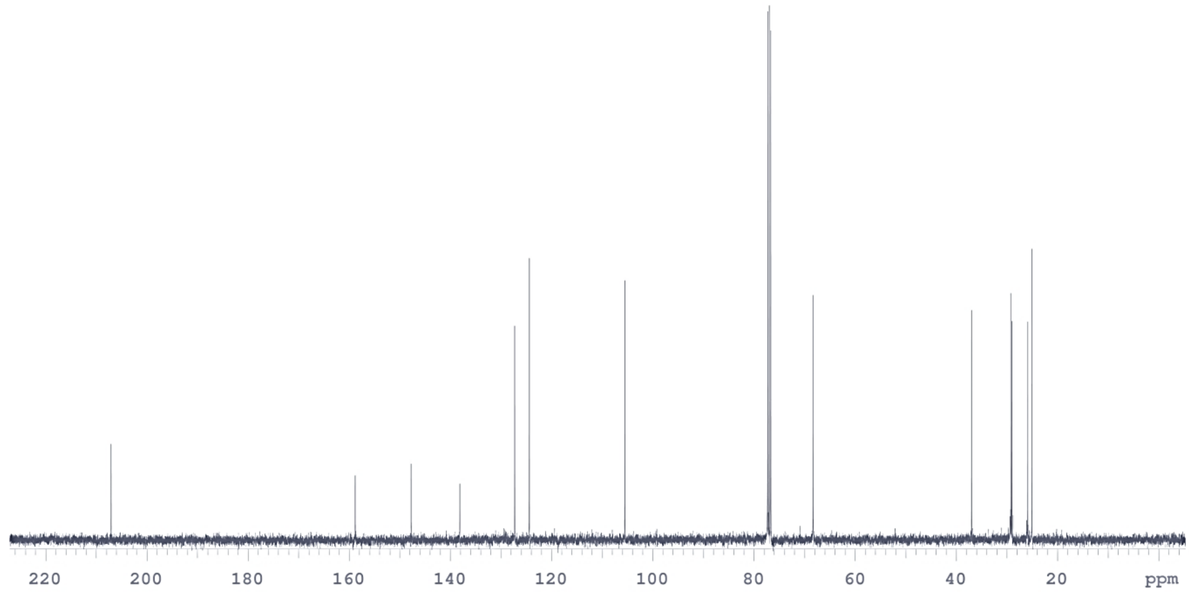

Figure S8. ^{13}C NMR spectrum of compound ***6b****.*

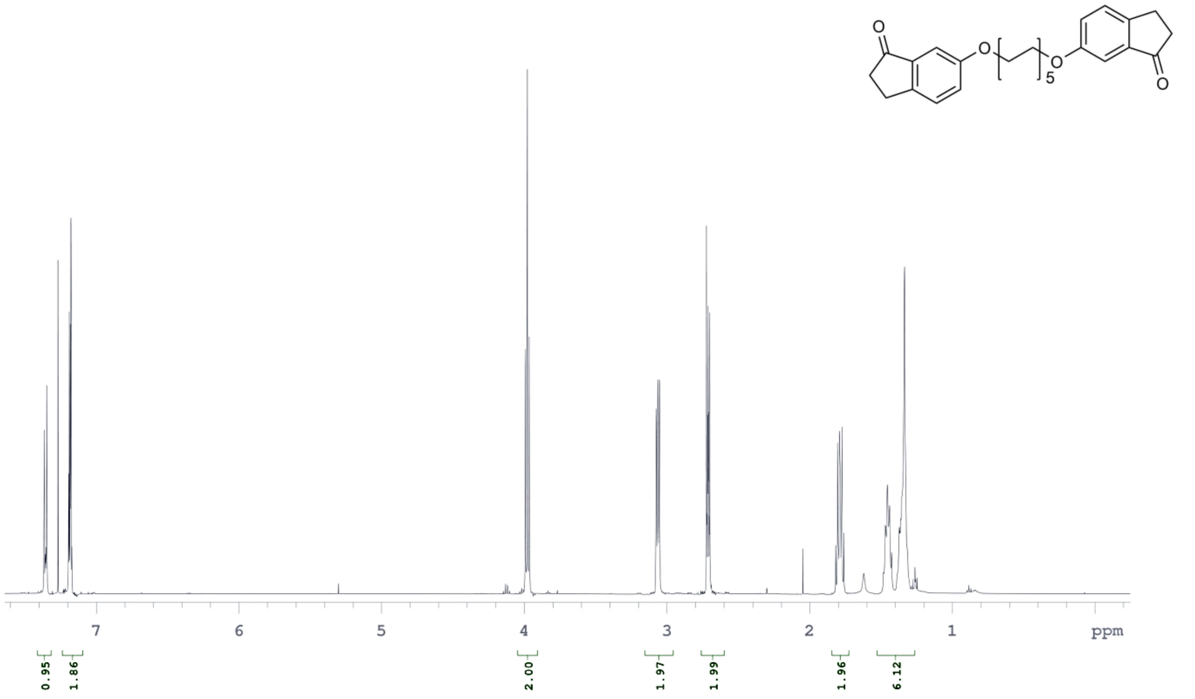

Figure S9. ^{1}H NMR spectrum of compound ***6c****.*

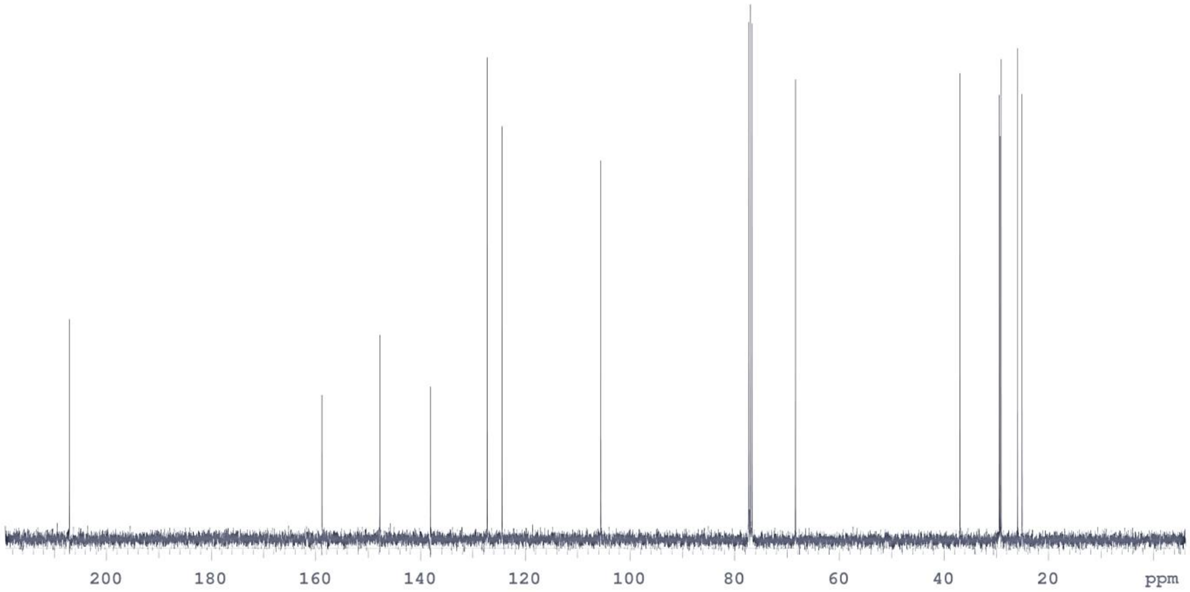

*Figure S10. ^{13}C NMR spectrum of compound **6c**.*

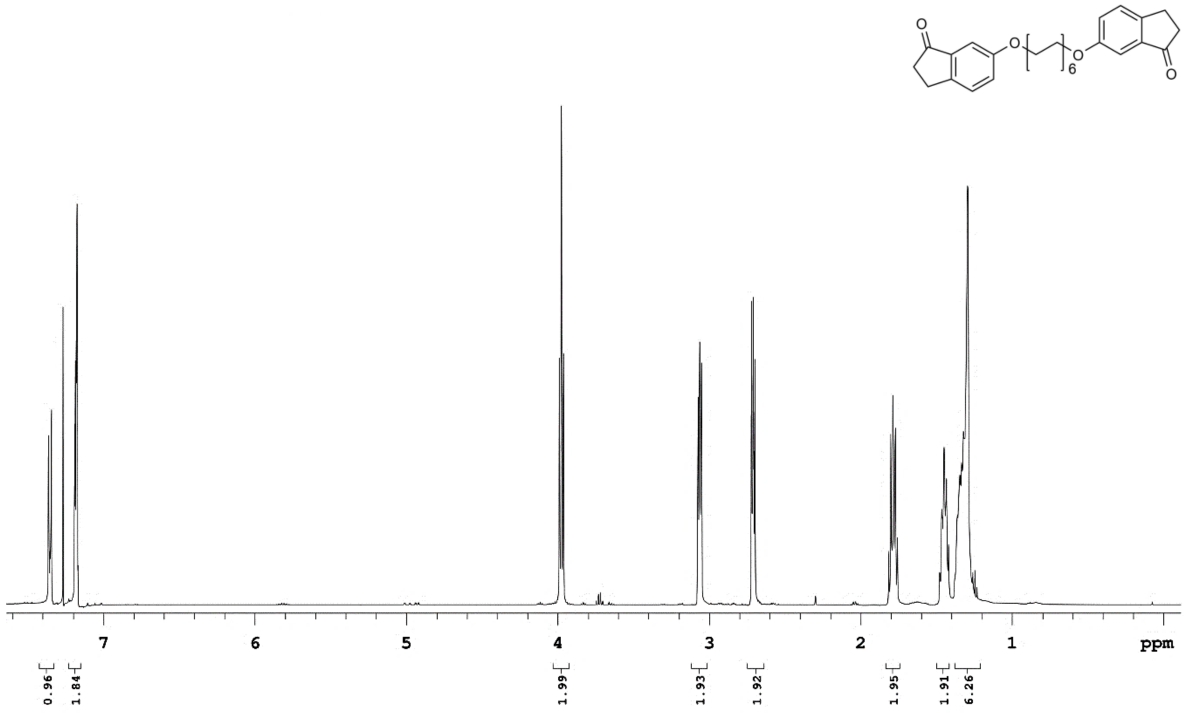

*Figure S11. ^{1}H NMR spectrum of compound **6d**.*

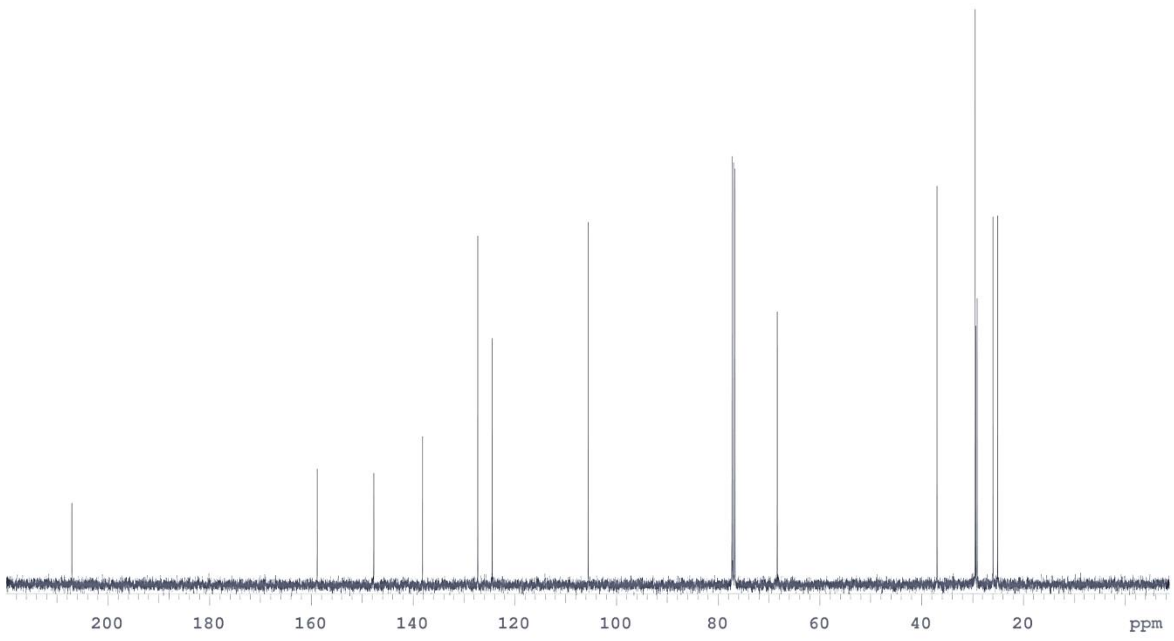

*Figure S12. ^{13}C NMR spectrum of compound **6d**.*

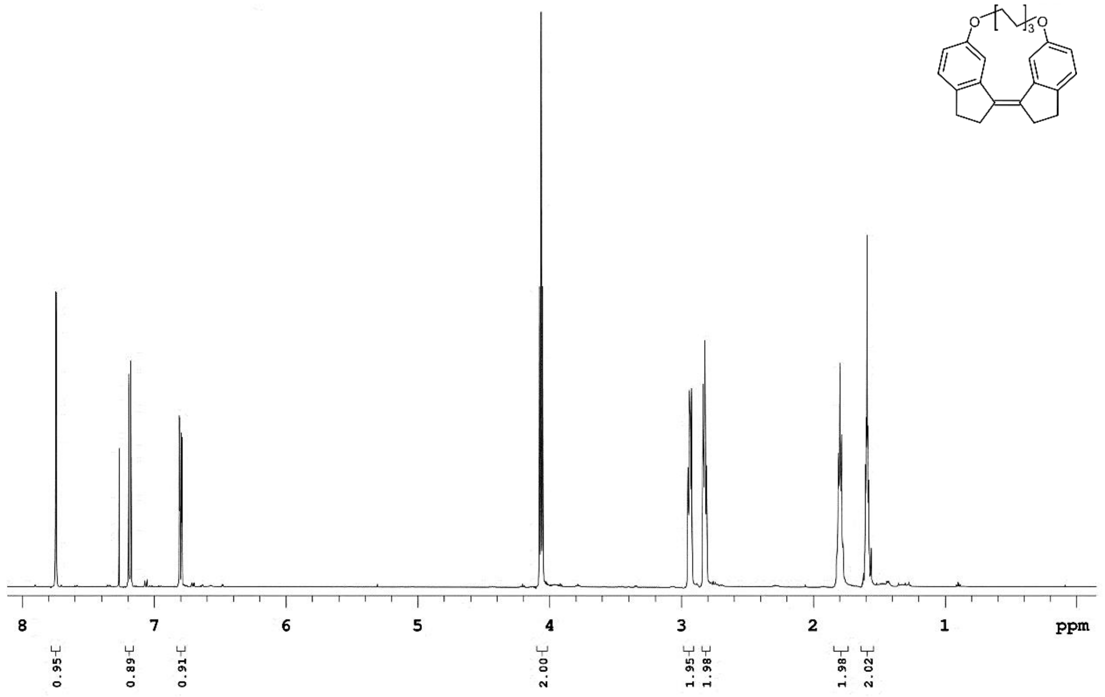

*Figure S13. ^{1}H NMR spectrum of compound (Z)-**1a**.*

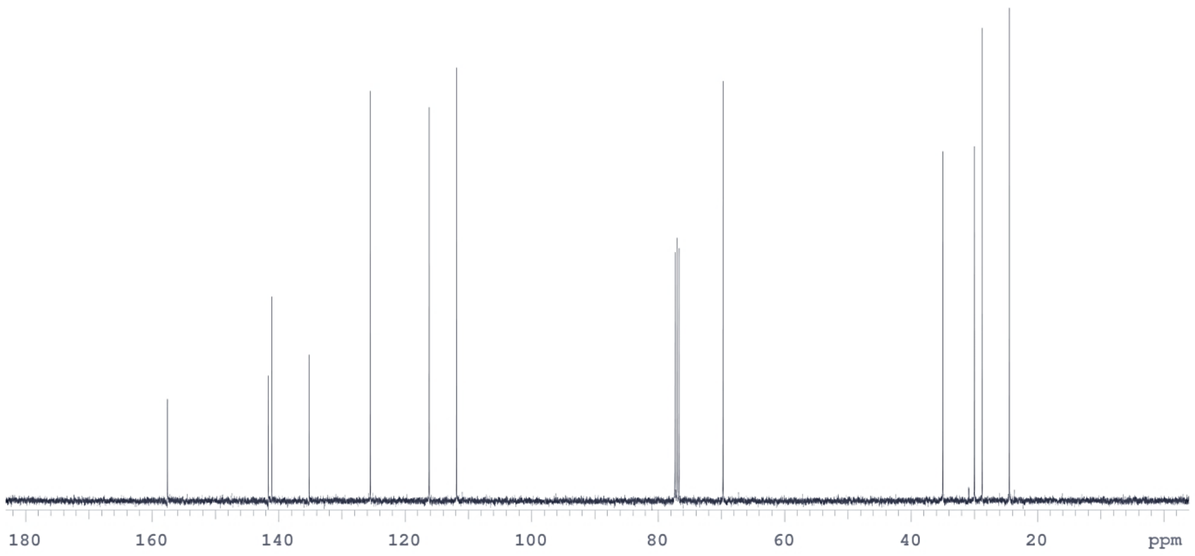

*Figure S14. ^{13}C NMR spectrum of compound (Z)-**1a**.*

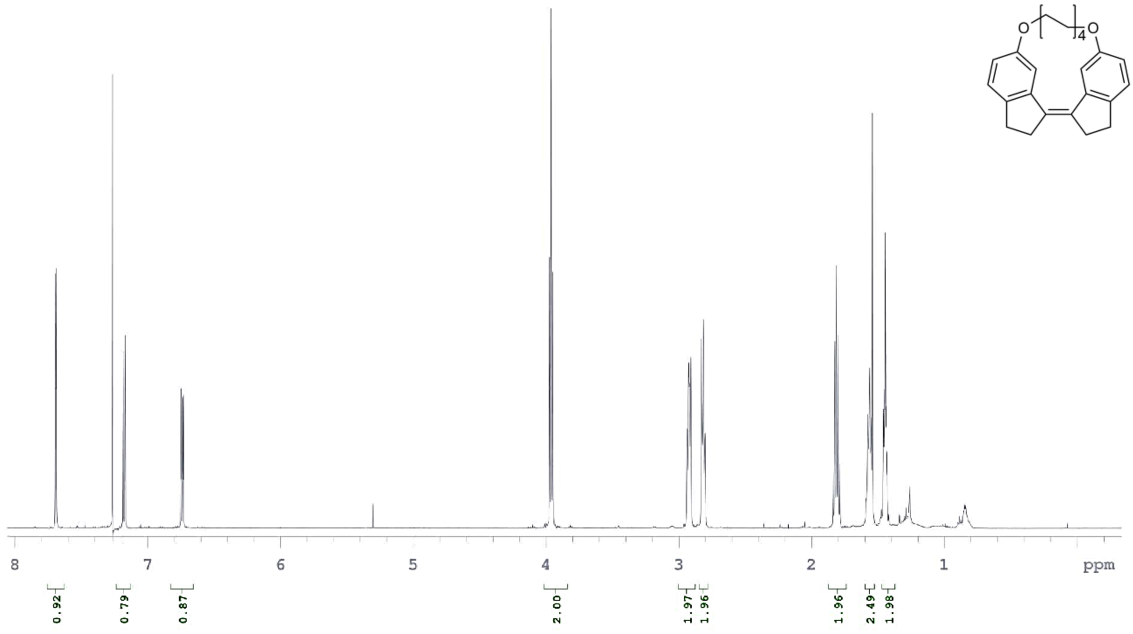

*Figure S15. ^{1}H NMR spectrum of compound (Z)-**1b**.*

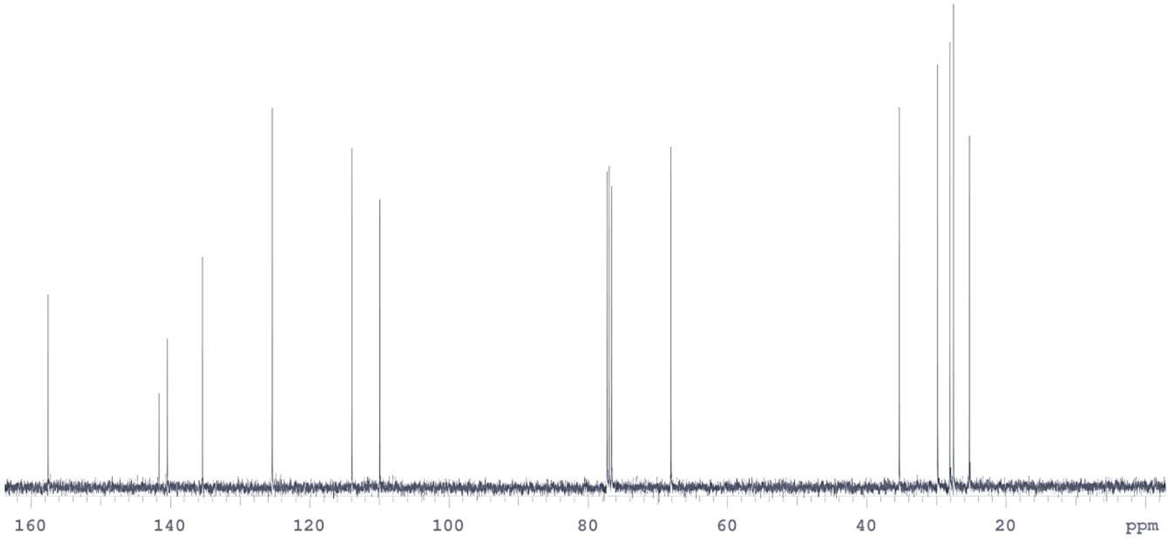

Figure S16. ^{13}C *NMR spectrum of compound (Z)-**1b**.*

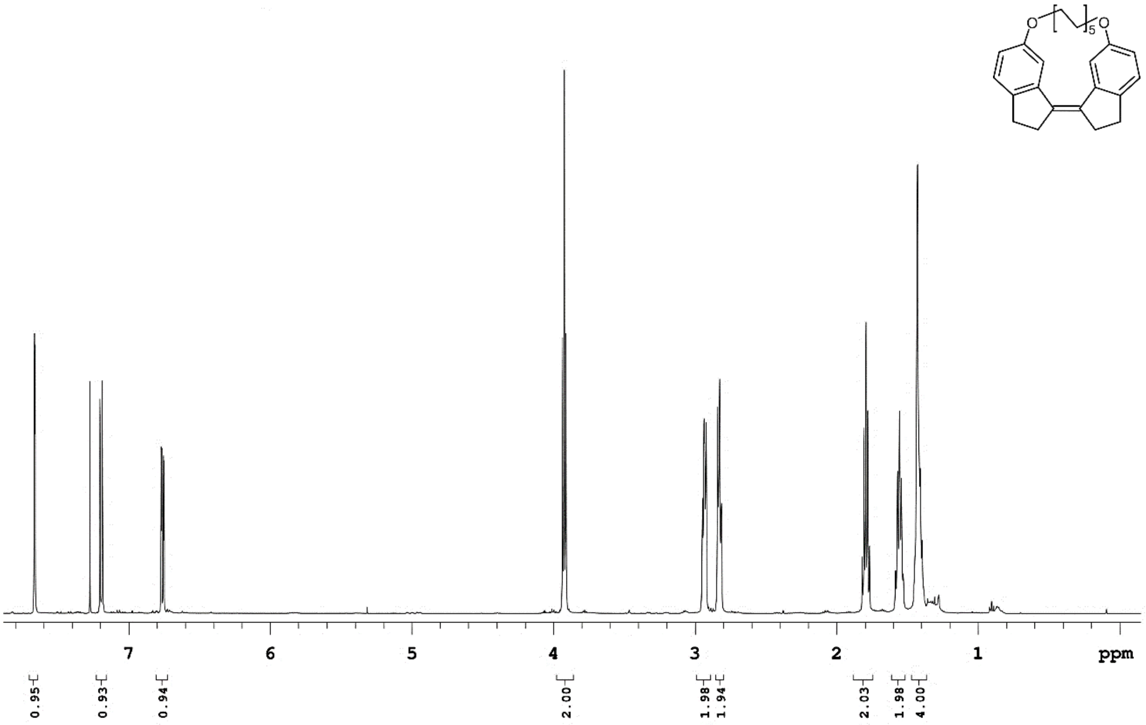

Figure S17. ^{1}H *NMR spectrum of compound (Z)-**1c**.*

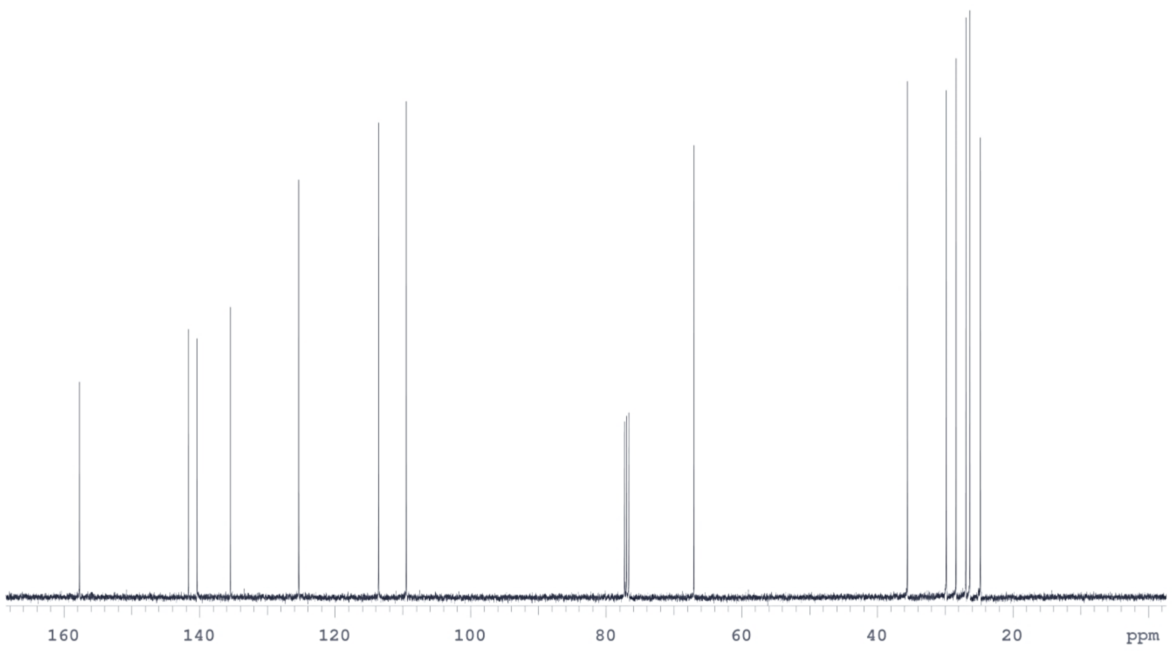

*Figure S18. ^{13}C NMR spectrum of compound (Z)-**1c**.*

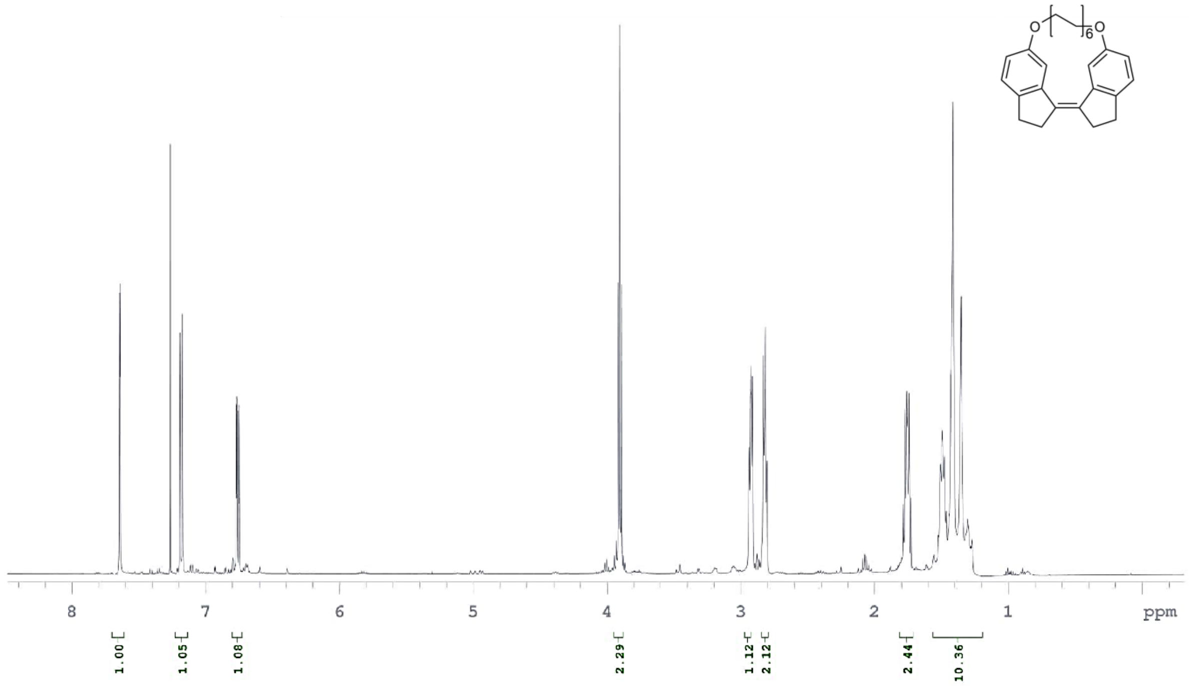

*Figure S19. ^{1}H NMR spectrum of compound (Z)-**1d**.*

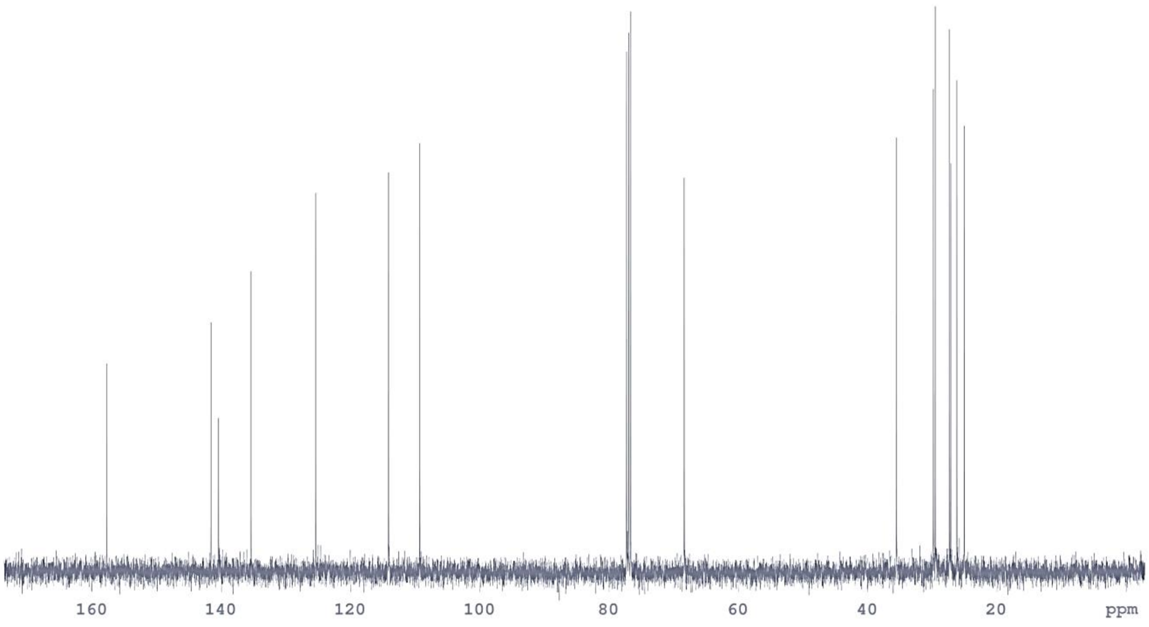

*Figure S20. ^{13}C NMR spectrum of compound (Z)-**1d**.*

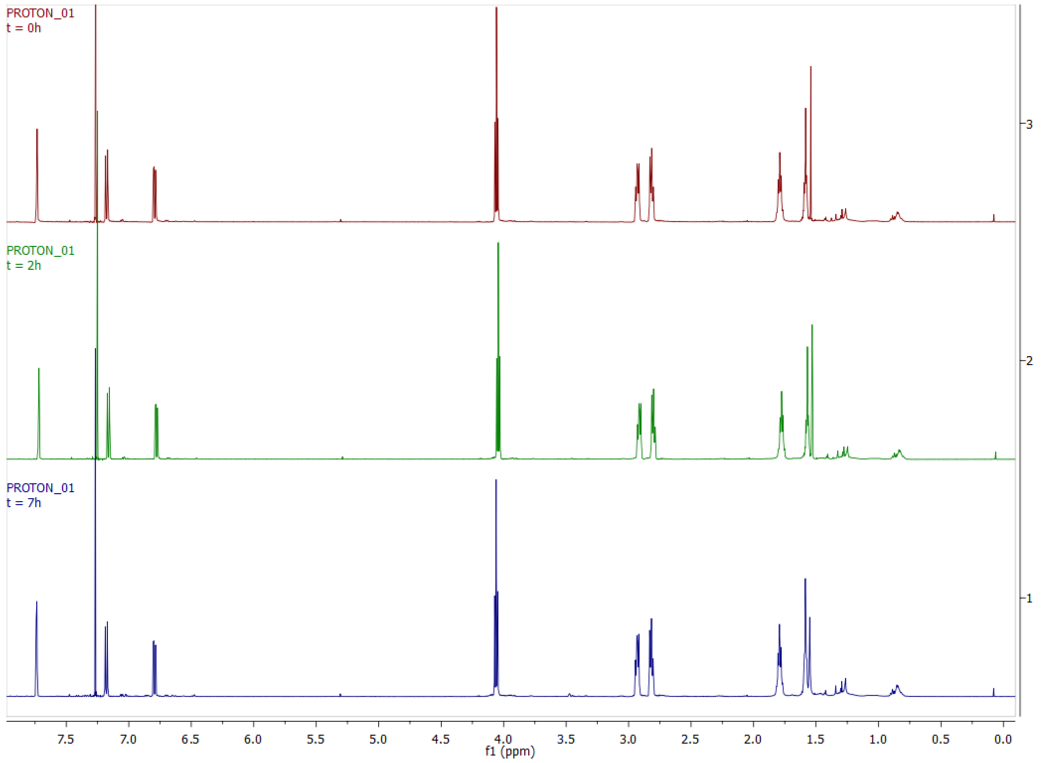

*Figure S21. Photo-isomerization process of compound (Z)-**1a** monitored by ^{1}H NMR spectroscopy. Initial sample (top), after 2 h (middle) and after 7 h (bottom).*

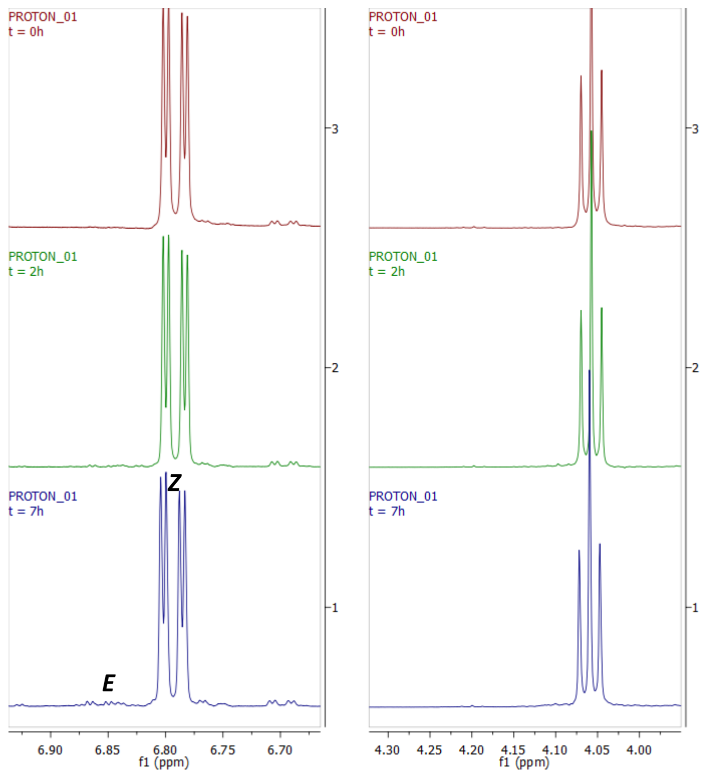

Figure S22. Expansions of ^{1}H *NMR spectra of Figure S21 showing an aromatic proton shift (left) and the signals corresponding to the hydrogens bonded to the first carbon of the linker between the two aromatic rings of stiff-stilbene (right).*

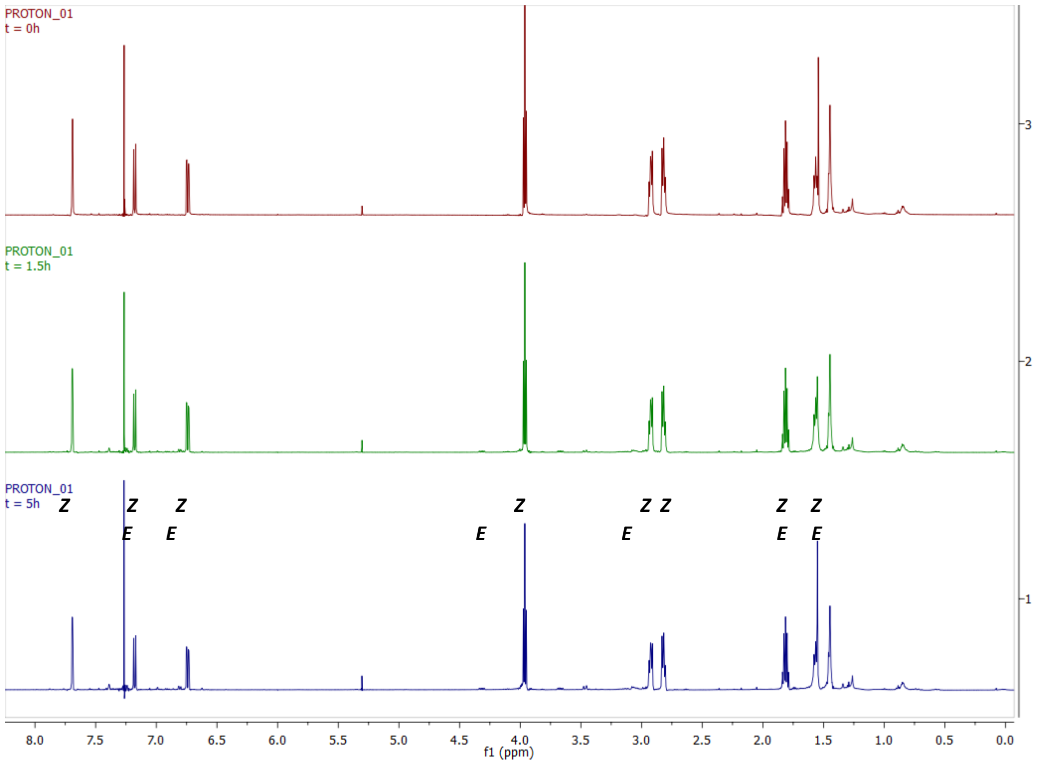

*Figure S23. Photo-isomerization process of compound (Z)-**1b** followed by* ^{1}H *NMR spectroscopy. Initial sample (top), after 1.5h (middle) and after 5h (bottom).*

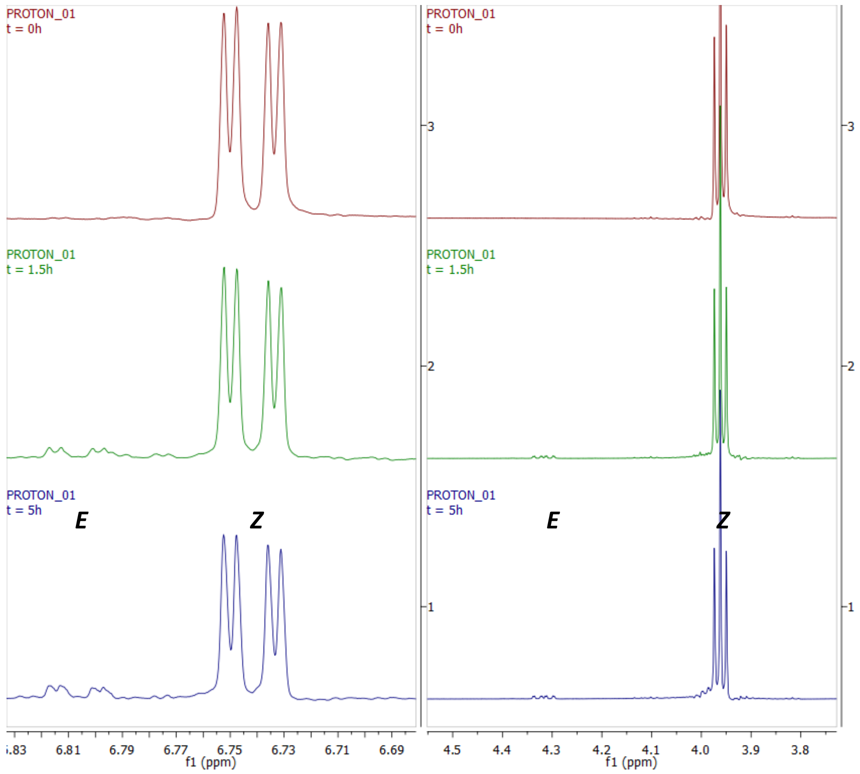

Figure S24. Expansions of ^1H NMR spectra of Figure S23 showing an aromatic proton chemical shift (left) and the signals corresponding to the hydrogens bonded to the first carbon of the linker between the two aromatic rings of stiff-stilbene (right).

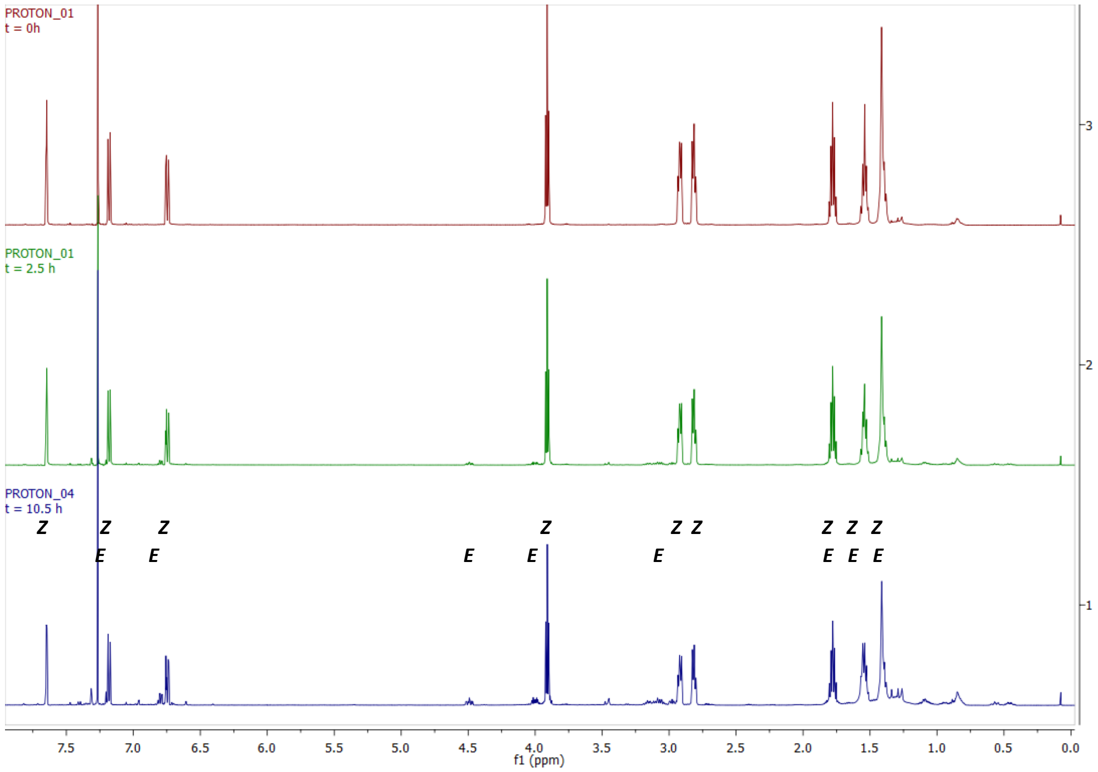

*Figure S25. Photo-isomerization process of compound (Z)-**1c** followed by ^1H NMR spectroscopy. Initial sample (top), mixture composition after 2.5h (middle) and after 10.5h (bottom).*

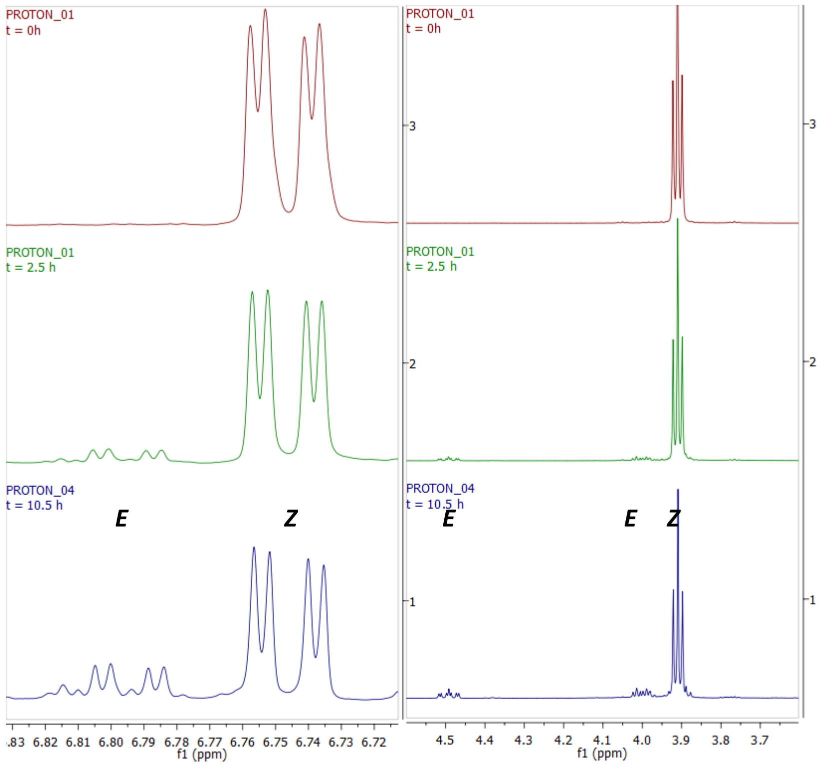

Figure S26. Expansions of ^{1}H NMR spectra of Figure S25 showing an aromatic proton shift (left) and the signals corresponding to the hydrogens bonded to the first carbon of the linker between the two aromatic rings of stiff-stilbene (right).

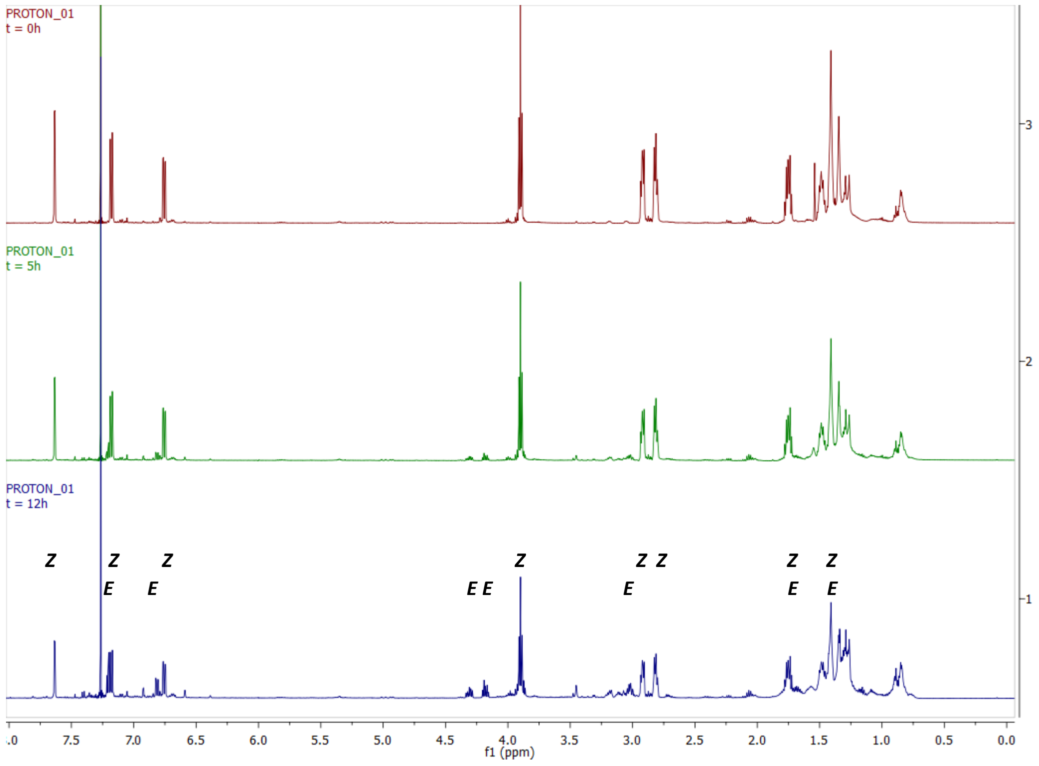

*Figure S27. Photo-isomerization process of compound (Z)-**1d** followed by ^{1}H NMR. Initial sample (top), mixture composition after 5 h (middle) and after 12 h (bottom).*

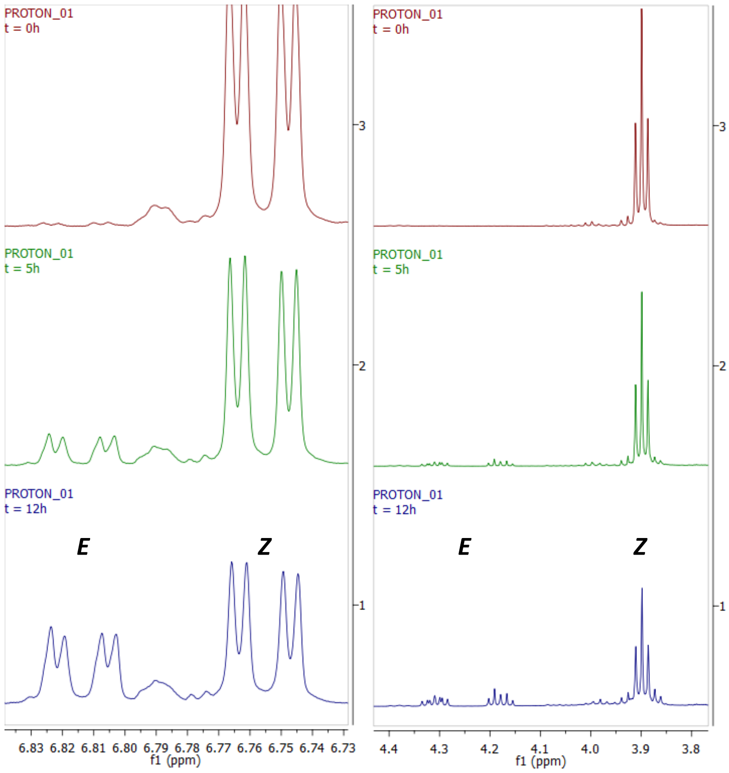

Figure S28. Expansions of ^{1}H NMR spectra of Figure S27 showing an aromatic proton shift (left) and the signals corresponding to the hydrogens bonded to the first carbon of the linker between the two aromatic rings of stiff-stilbnene.(right).

2. UV–vis spectra

UV–vis spectra of synthesized compounds.

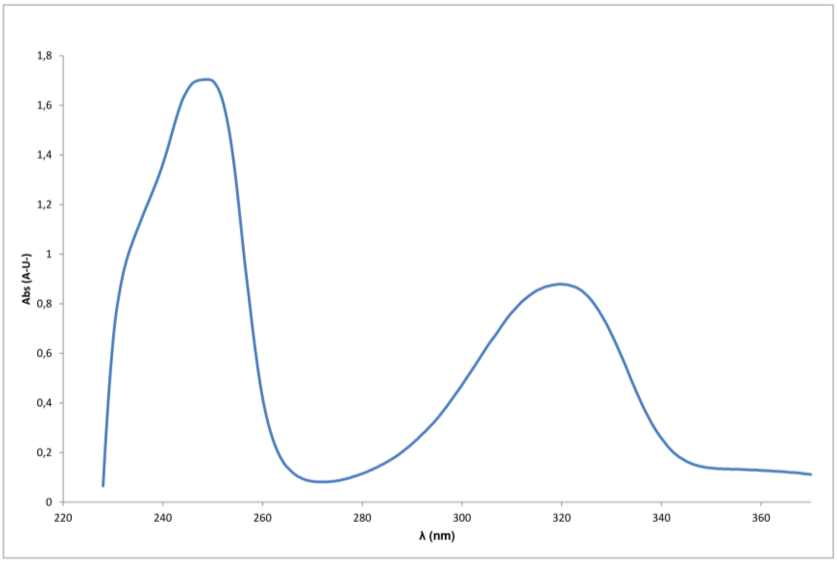

*Figure S29. UV–vis spectrum of compound **6a**.*

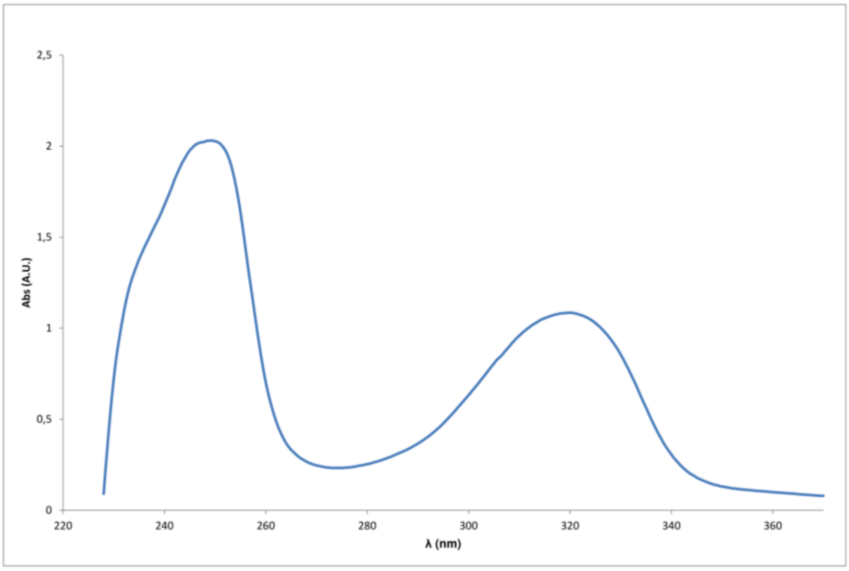

Figure S30. UV–vis spectrum of compound ***6b****.*

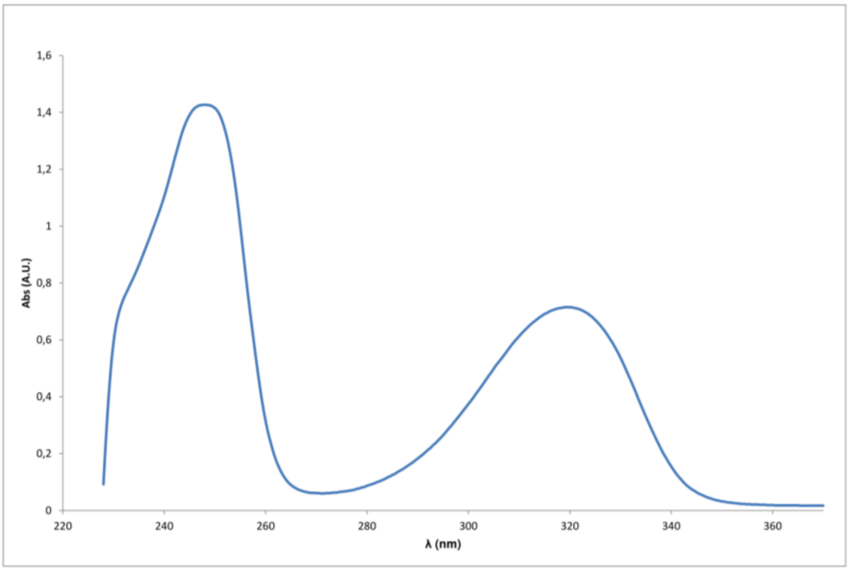

Figure S31. UV–vis spectrum of compound ***6c****.*

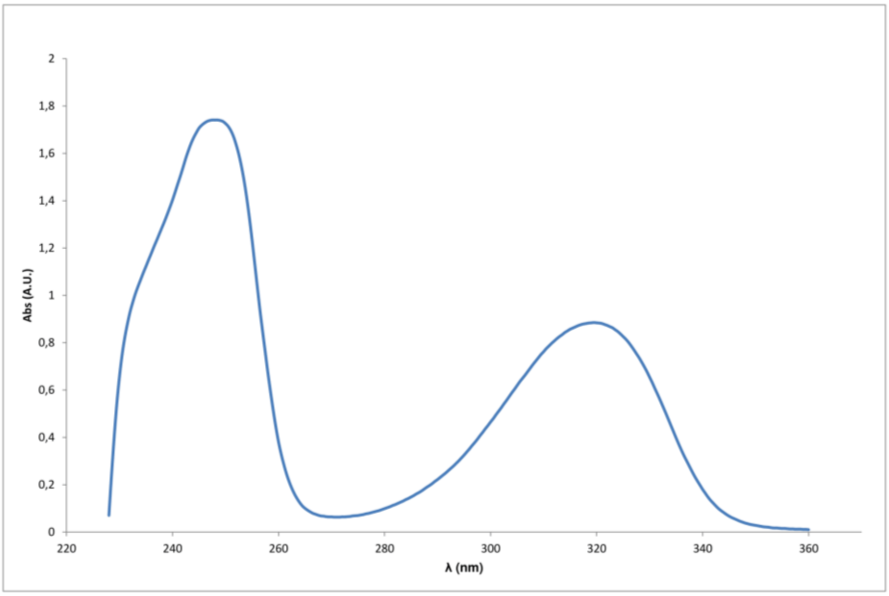

*Figure S32. UV–vis spectrum of compound **6d**.*

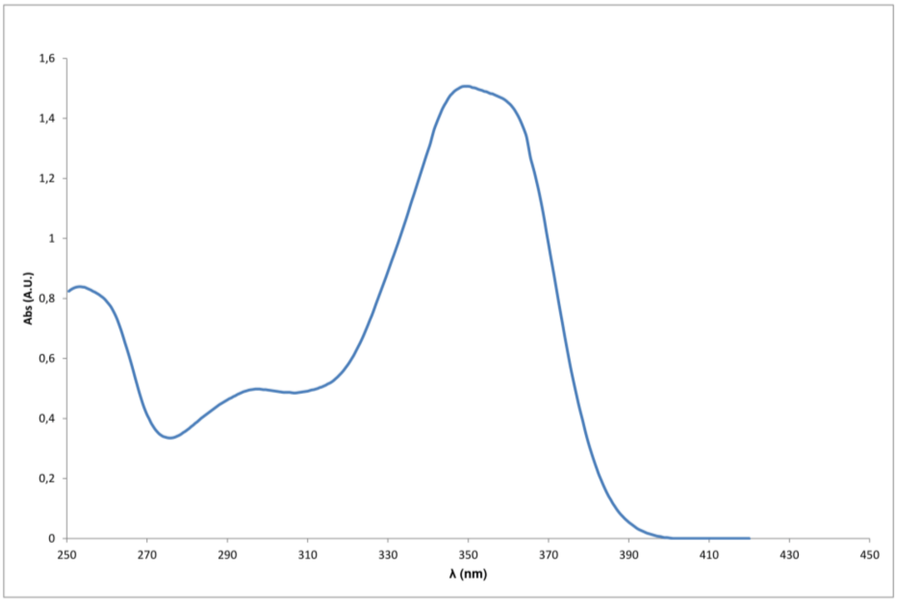

*Figure S33. UV–vis spectrum of compound (Z)-**1a**.*

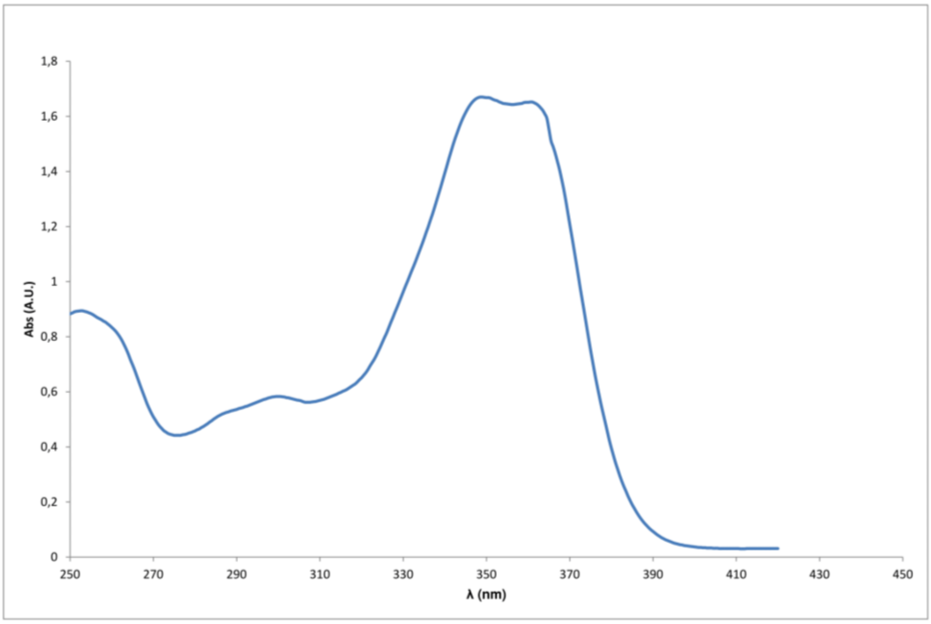

*Figure S34. UV–vis spectrum of compound (Z)-**1b**.*

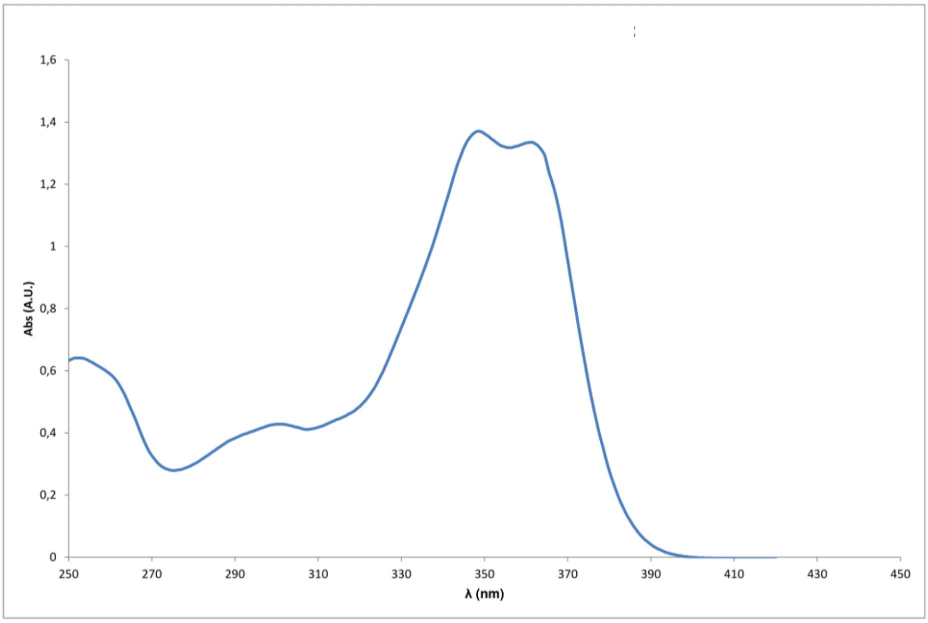

*Figure S35. UV–vis spectrum of compound (Z)-**1c**.*

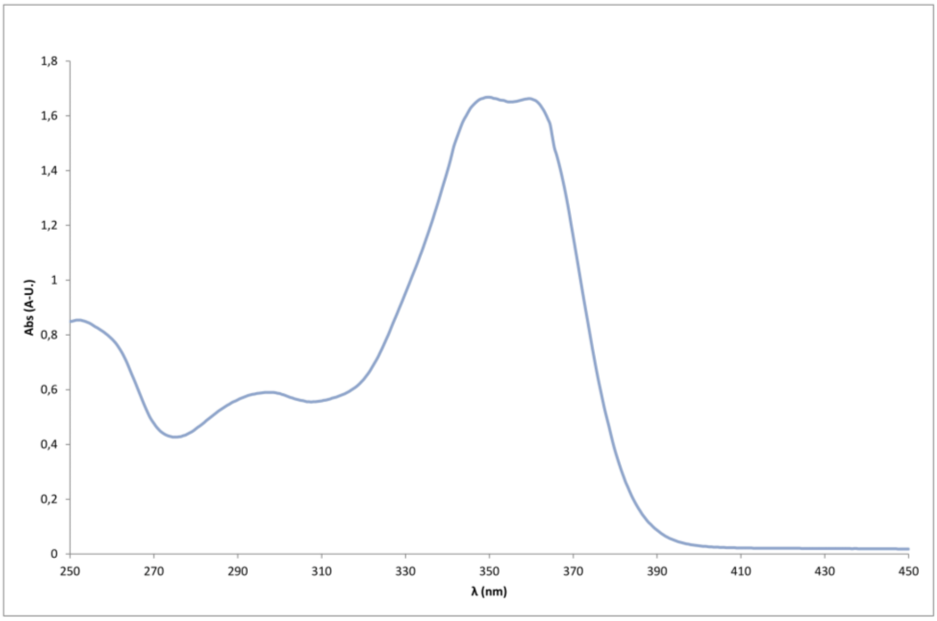

*Figure S36. UV–vis spectrum of compound (Z)-**1d**.*

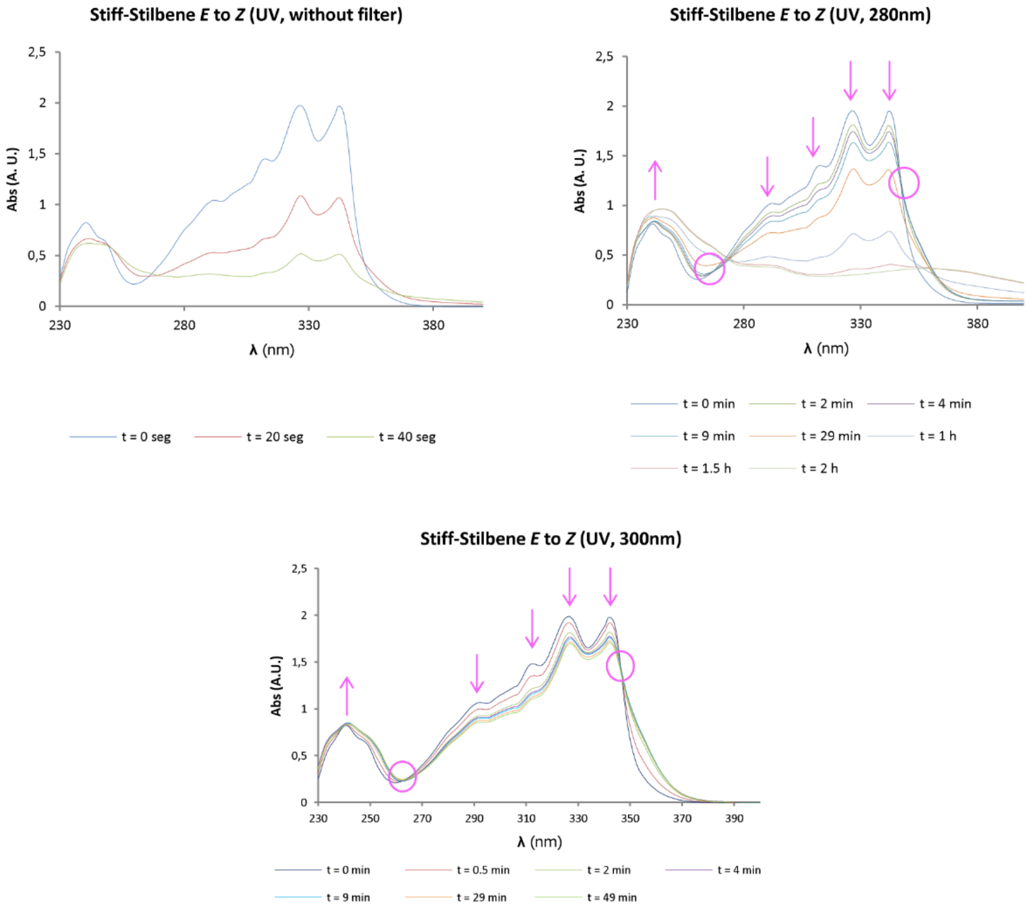

Figure S37. Photo-isomerizations from unfunctionalized *(E)*-stiff-stilbene to *(Z)*-stiff-stilbene followed by UV-vis spectroscopy. The samples were irradiated with UV light without filter (top, left), UV light of 280 nm (top, right) and UV light of 300 nm (bottom).

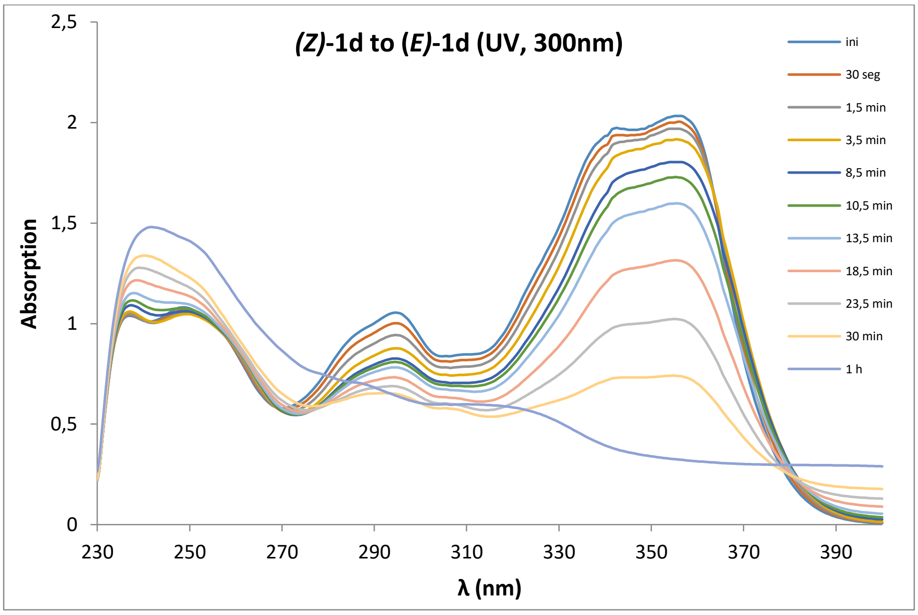

Figure S38. Photo-isomerization from *(E)*-**1d** to *(Z)*-**1d** followed by UV-vis spectroscopy. The sample was irradiated with UV light of 300 nm.

3. Conformational analysis

Data from MM (OPLS3) conformational analysis.

***Z*-1a** conformational analysis

Entry ID	Potential Energy-OPLS3e	RMS Derivative-OPLS3e	Relative Potential Energy-OPLS3e	Boltzmann Population	enatiomers	Entry ID	Boltzmann Population	Boltzmann Population w/o enantiomers
1	16.416	0.039	0	17.510664		1+2	17.510664	34.945729
2	16.427	0.037	0.011	17.435065	*		17.435065	
3	18.161	0.046	1.744	8.663045		3+4	8.663045	17.304307
4	18.167	0.036	1.751	8.641262	*		8.641262	
5	19.086	0.034	2.67	5.963962		5+6	5.963962	11.900138
6	19.098	0.032	2.681	5.936176	*		5.936176	
7	19.258	0.038	2.841	5.565757		7+8	5.565757	11.116891
8	19.264	0.045	2.848	5.551134	*		5.551134	
9	20.343	0.042	3.927	3.591659		9+10	3.591659	7.171812
10	20.351	0.041	3.935	3.580153	*		3.580153	
11	20.472	0.036	4.055	3.41045	*	11+12	3.41045	6.804958
12	20.483	0.049	4.067	3.394508			3.394508	
13	20.962	0.04	4.546	2.798056		13+14	2.798056	6.192564
14	20.987	0.045	4.571	2.769804	*		2.769804	
15	21.149	0.034	4.733	2.594582	*	15+16	2.594582	5.188306
16	21.15	0.033	4.734	2.593724			2.593724	
								100.000001

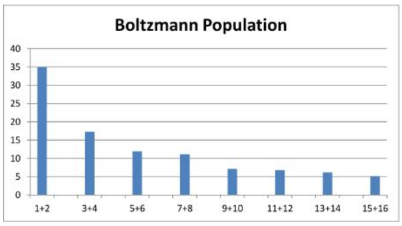

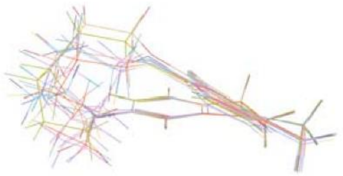

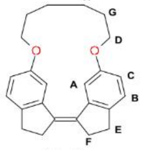

Z-1a, C_6

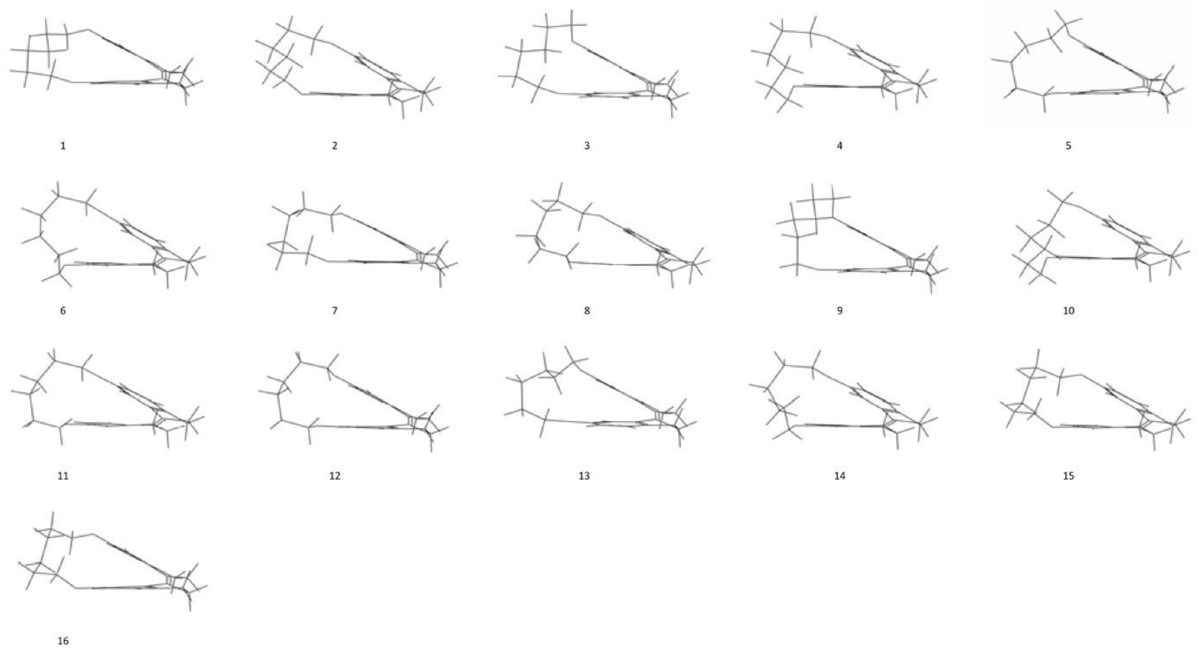

*Figure S39. Conformational analysis of (Z)-**1a**.*

***E* -1a** conformational analysis

Entry ID	Potential Energy-OPLS3e	RMS Derivative-OPLS3e	Relative Potential Energy-OPLS3e	Boltzmann Population
1	169.749	0.047	0	65.368285
2	172.751	0.042	3.002	19.470553
3	174.337	0.033	4.589	10.266941
4	178.188	0.027	8.44	2.171404
5	178.905	0.031	9.157	1.625883
6	180.584	0.047	10.836	0.825936
7	186.724	0.047	16.975	0.069386
8	187.738	0.029	17.989	0.046089
9	188.912	0.033	19.164	0.028699
10	188.921	0.039	19.173	0.028593
11	189.682	0.031	19.934	0.021035
12	189.718	0.028	19.97	0.020733
13	189.8	0.035	20.052	0.020055
14	189.902	0.04	20.154	0.019248
15	190.187	0.03	20.438	0.01716
				100

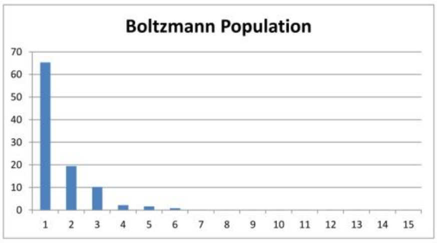

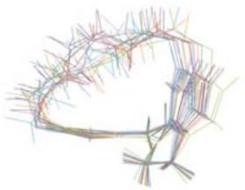

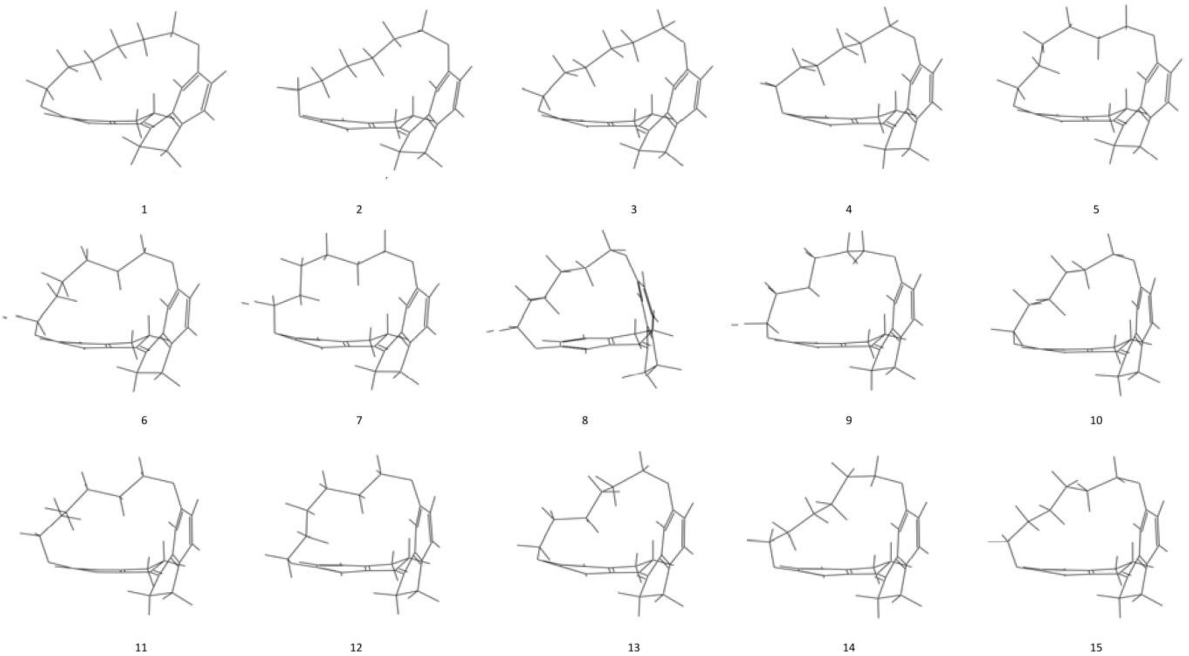

*Figure S40. Conformational analysis of (E)-**1a**.*

***Z*-1b** conformational analysis

Entry ID	Potential Energy-OPLS3e	RMS Derivative-OPLS3e	Relative Potential Energy-OPLS3e	Boltzmann Population	enatiomer	Entries	Boltzmann Population w/o enantiomers
1	0.8	0.043	0	28.280308		1	28.280308
2	3.265	0.034	2.465	10.463016		2+3	20.922542
3	3.266	0.041	2.466	10.459526	*	8+9+10+11	16.531972
4	3.899	0.042	3.099	8.1002	*	4+5	16.177061
5	3.906	0.043	3.106	8.076861		6	6.550279
6	4.426	0.045	3.626	6.550279		7	6.247828
7	4.543	0.048	3.743	6.247828		12+13	5.290011
8	5.475	0.037	4.675	4.290365			
9	5.479	0.042	4.679	4.282768	*		
10	5.653	0.039	4.853	3.992008	*		
11	5.669	0.045	4.869	3.966831	*		
12	6.65	0.038	5.85	2.670545	*		
13	6.698	0.046	5.898	2.619466			
							100.000001

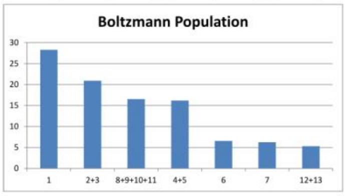

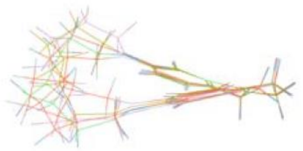

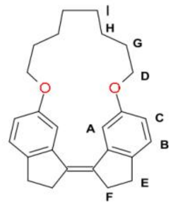

Z-1b, C_8

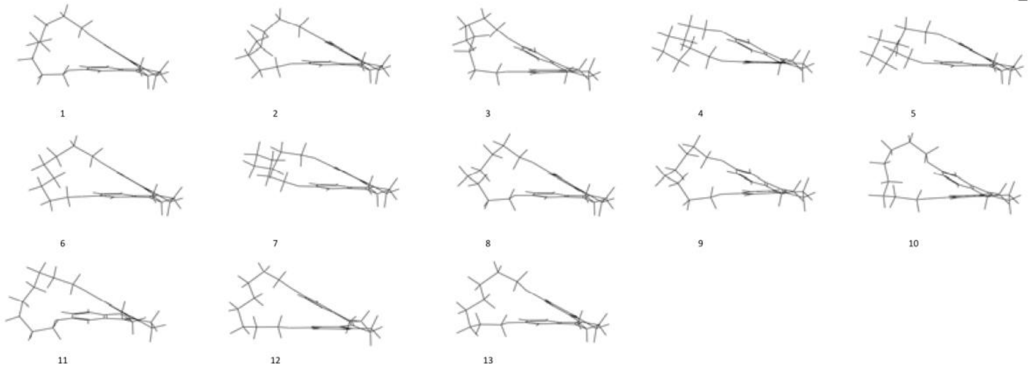

*Figure S41. Conformational analysis of (Z)-**1b**.*

***E*-1b** conformational analysis

Entry ID	Potential Energy-OPLS3e	RMS Derivative-OPLS3e	Relative Potential Energy-OPLS3e	Boltzmann Population
1	71.765	0.035	0	91.534935
2	77.709	0.042	5.944	8.320544
3	87.756	0.034	15.991	0.144521

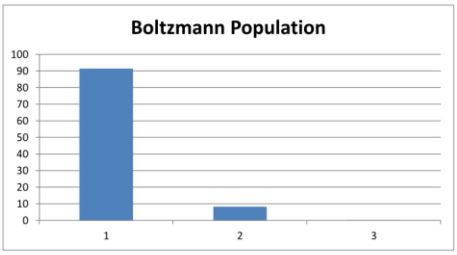

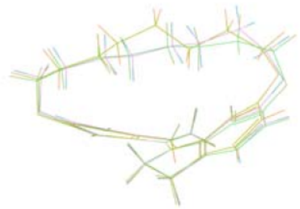

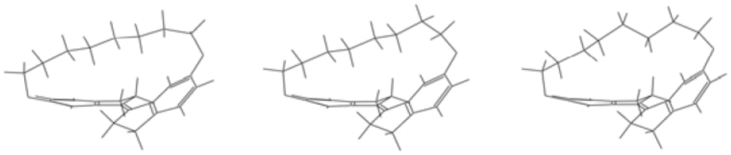

*Figure S42. Conformational analysis of (E)-**1b**.*

***Z*-1c** conformational analysis

Entry ID	Potential Energy-OPLS3e	RMS Derivative-OPLS3e	Relative Potential Energy-OPLS3e	Boltzmann Population	enatiomers	Entry ID	Boltzmann Population w/o enantiomers
1	0.765	0.041	0	36.706599		1+2	69.900258
2	1.015	0.035	0.249	33.193659	*	3	8.113675
3	4.507	0.049	3.742	8.113675	*	5+6	7.824915
4	6.103	0.045	5.338	4.260882	*	4	4.260882
5	6.314	0.027	5.548	3.914211	*	7+8	2.674599
6	6.316	0.035	5.551	3.910704		11+12	2.01951
7	8.965	0.044	8.2	1.343151	*	14+15	1.718937
8	8.987	0.044	8.221	1.331448		9	1.273828
9	9.096	0.05	8.331	1.273828		10	1.243654
10	9.156	0.042	8.391	1.243654		13	0.969741
11	9.658	0.046	8.893	1.015556			
12	9.686	0.042	8.921	1.003954	*		
13	9.772	0.03	9.007	0.969741	*		
14	9.991	0.045	9.226	0.887901	*		
15	10.155	0.042	9.39	0.831036			
							99.999999

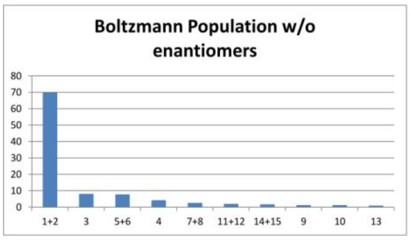

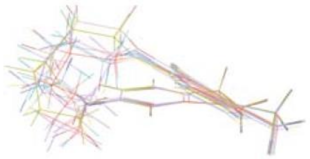

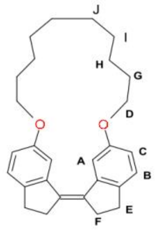

Z-1c, C_{10}

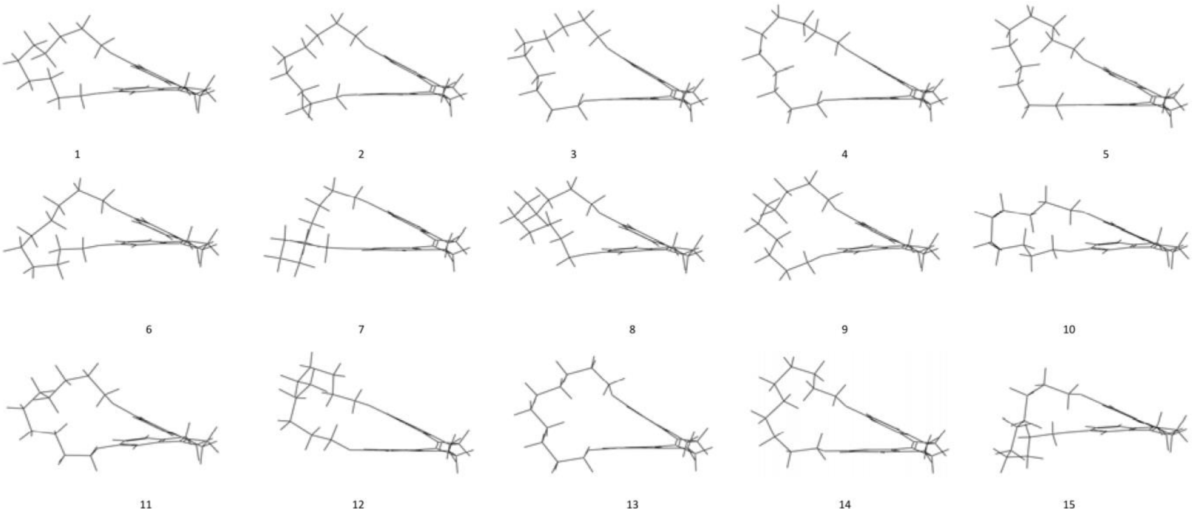

*Figure S43. Conformational analysis of (Z)-**1c**.*

***E* -1c** conformational analysis

Entry ID	Potential Energy-OPLS3e	RMS Derivative-OPLS3e	Relative Potential Energy-OPLS3e	Boltzmann Population
1	17.733	0.04	0	90.043783
2	24.439	0.044	6.706	6.020084
3	27.67	0.024	9.937	1.634638
4	29.576	0.036	11.843	0.757804
5	31.661	0.038	13.928	0.32676
6	32.8	0.043	15.068	0.206338
7	33.436	0.041	15.703	0.15965
8	33.445	0.03	15.712	0.159099
9	34.897	0.047	17.164	0.088555
10	35.014	0.037	17.281	0.084489
11	35.322	0.035	17.59	0.074595
12	35.41	0.028	17.677	0.071999
13	35.929	0.048	18.196	0.058405
14	36.304	0.035	18.571	0.050205
15	36.496	0.034	18.763	0.046469
16	36.867	0.033	19.134	0.040009
17	37.444	0.045	19.711	0.031699
18	37.518	0.046	19.785	0.030765
19	37.91	0.044	20.177	0.026261
20	37.92	0.031	20.187	0.026163
21	38.349	0.041	20.616	0.022005
22	38.427	0.045	20.694	0.021318
23	38.725	0.038	20.992	0.018905

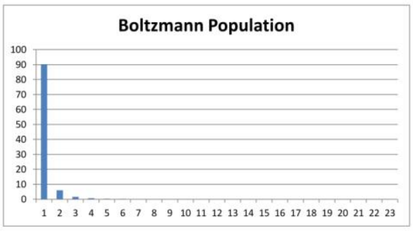

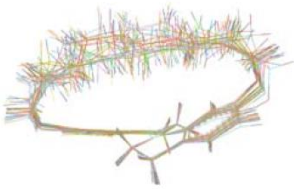

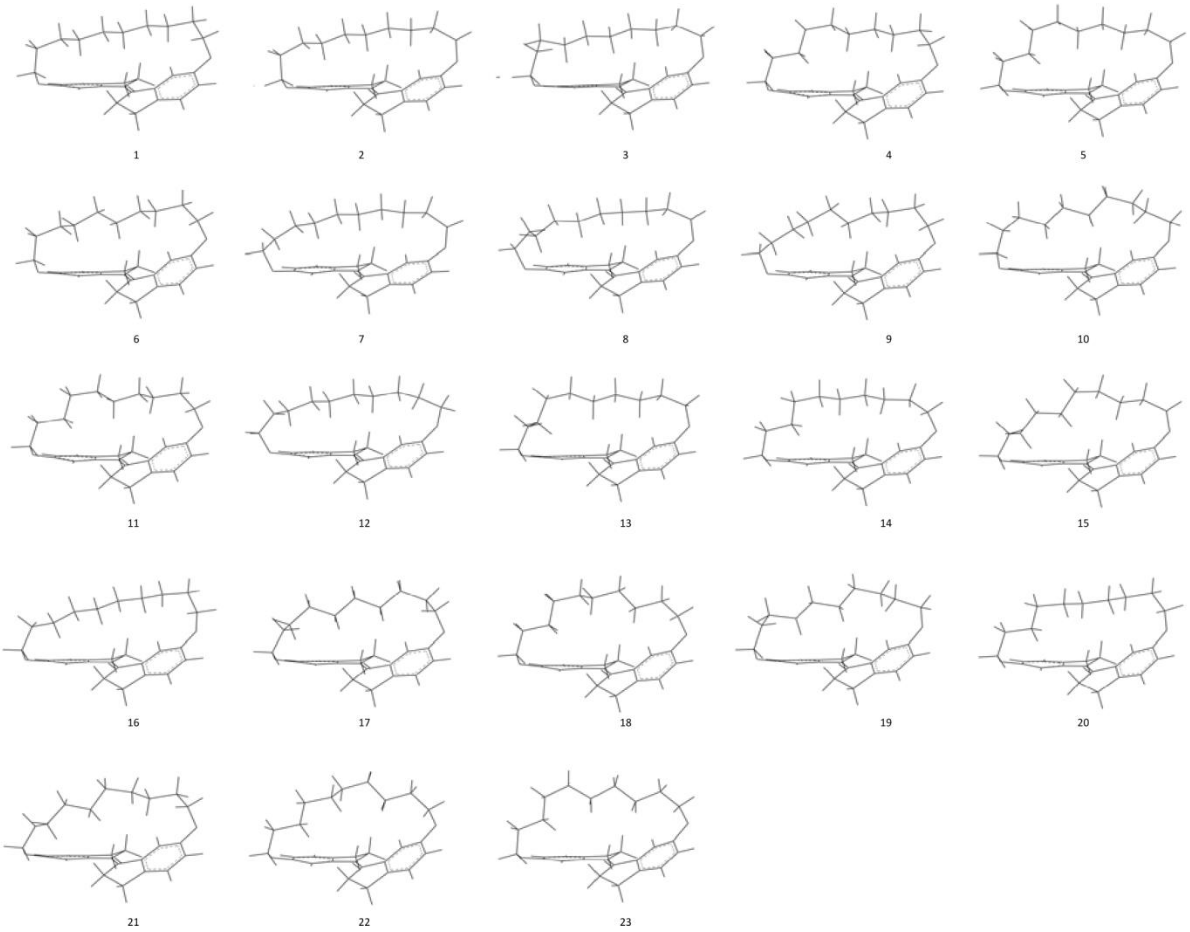

*Figure S44. Conformational analysis of (E)-**1c**.*

***Z*-1d** conformational analysis

Entry ID	Potential Energy-OPLS3e	RMS Derivative-OPLS3e	Relative Potential Energy-OPLS3e	Boltzmann Population	enatiomers	Entry ID	Boltzmann Population w/o enantiomers
1	7.765	0.041	0	45.496109		1	45.4961
2	11.678	0.042	3.914	9.382472		2	9.3825
3	12.079	0.049	4.314	7.982229		3	7.9822
4	12.18	0.046	4.415	7.663767		4	7.6638
5	12.644	0.026	4.879	6.356358		5	6.3564
6	14.392	0.047	6.627	3.139533		10+11	3.7215
7	14.466	0.032	6.701	3.047318		6	3.1395
8	14.993	0.048	7.229	2.463321		7	3.0473
9	15.641	0.042	7.876	1.897164		14+15	2.7615
10	15.675	0.034	7.91	1.870885		8	2.4633
11	15.702	0.04	7.937	1.850606	*	17+18	2.0846
12	16.235	0.041	8.471	1.492442		9	1.8972
13	16.38	0.036	8.616	1.4077		12	1.4924
14	16.397	0.041	8.632	1.398119		13	1.4077
15	16.46	0.035	8.695	1.363372	*	16	1.1040
16	16.983	0.039	9.218	1.103958			
17	17.12	0.029	9.355	1.044591			
18	17.131	0.038	9.366	1.040056	*		

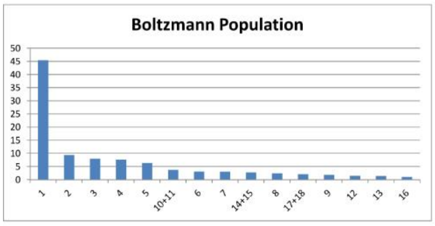

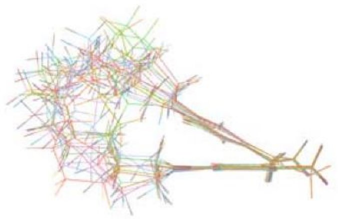

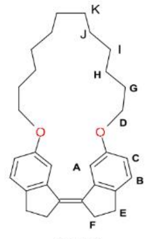

Z-1d, C_{12}

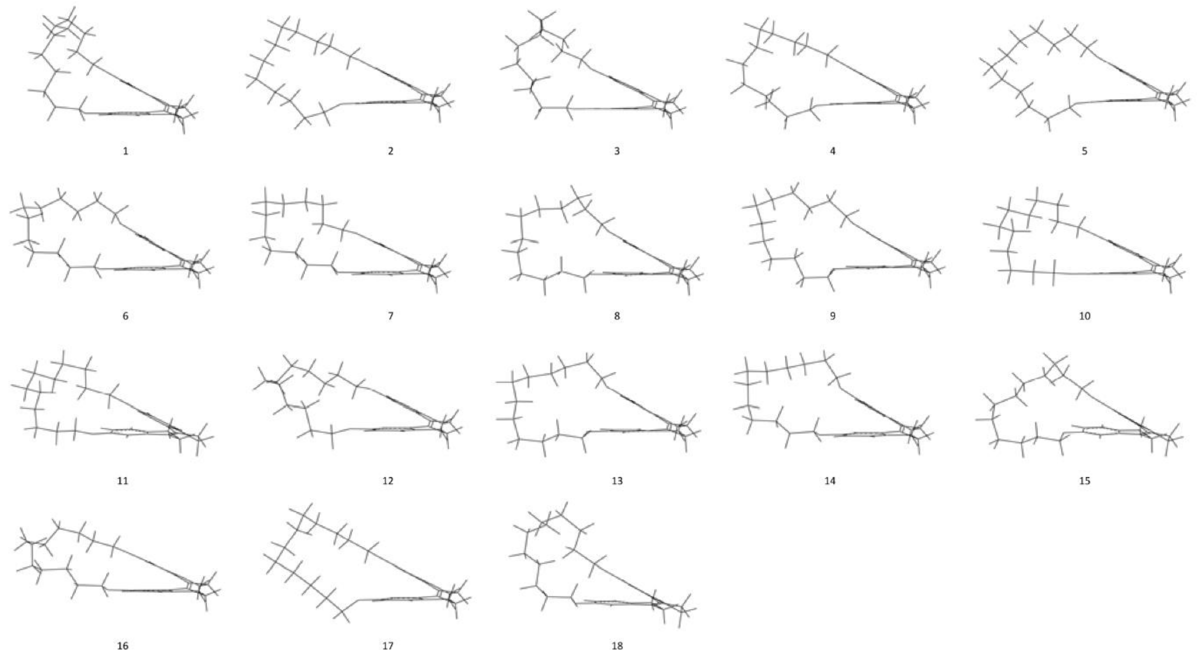

*Figure S45. Conformational analysis of (Z)-**1d**.*

E **-1d** conformational analysis

Entry ID	Potential Energy-OPLS3e	RMS Derivative-OPLS3e	Relative Potential Energy-OPLS3e	Boltzmann Population	enantiomer	Entry ID	Boltzmann Population
1	7.85	0.032	0	16.70512		1	16.70512
2	8.544	0.039	0.694	12.624384		2	12.624384
3	9.024	0.043	1.174	10.404704		3	10.404704
4	9.353	0.048	1.503	9.109486		4	9.109486
5	9.913	0.041	2.063	7.267577	*	5	7.267577
6	10.227	0.046	2.376	6.40449		6	6.40449
7	11.041	0.045	3.191	4.610556		7	4.610556
8	11.471	0.024	3.621	3.876944		8	3.876944
9	11.486	0.031	3.636	3.85389		9	3.85389
10	11.814	0.027	3.964	3.375726	*	10	3.375726
11	11.839	0.046	3.989	3.341298	*	11	3.341298
12	12.254	0.043	4.404	2.82645		12	2.82645
13	12.551	0.04	4.701	2.507599		13	2.507599
14	12.636	0.049	4.786	2.423038		14	2.423038
15	13.194	0.024	5.344	1.934374		15	1.934374
16	13.199	0.048	5.349	1.930384		16	1.930384
17	13.391	0.043	5.541	1.786966		17	1.786966
18	13.394	0.024	5.544	1.784469		18	1.784469
19	13.482	0.034	5.632	1.722437		19	1.722437
20	13.808	0.041	5.958	1.510107		20	1.510107

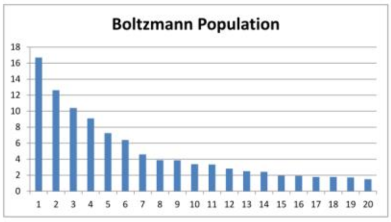

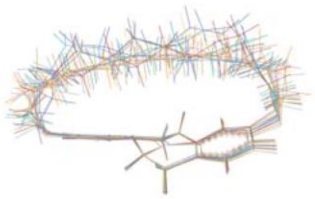

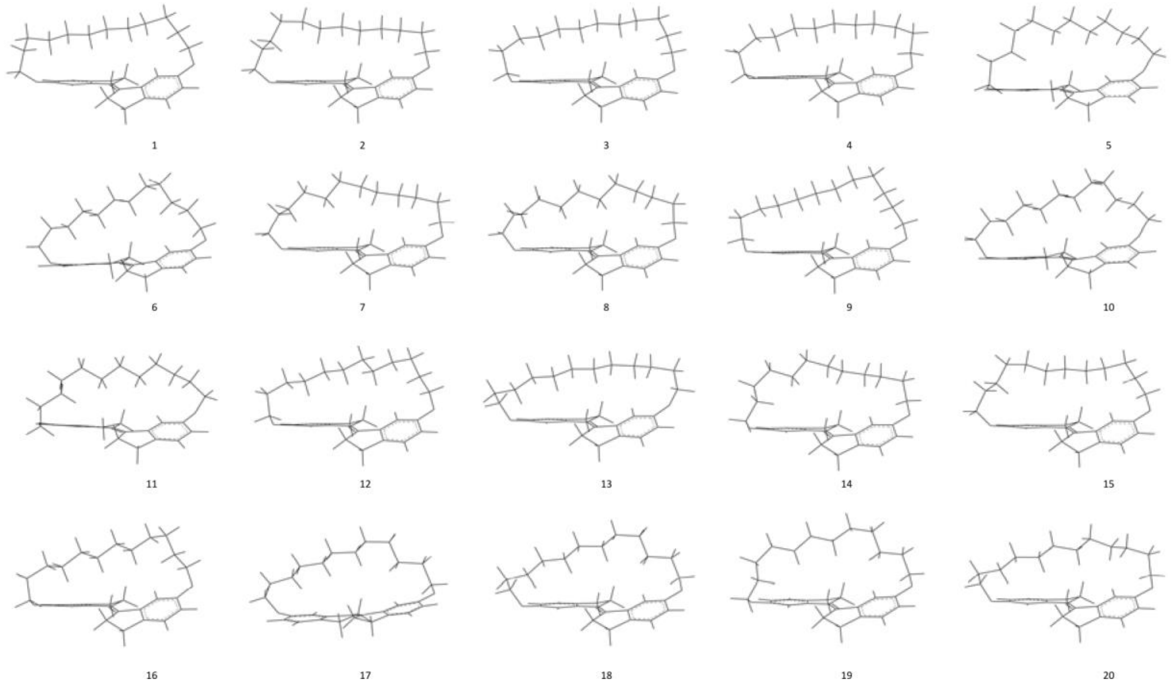

*Figure S46. Conformational analysis of (E)-**1d**.*

4. NOE buildup

*Table S1. NOE buildup for (Z)-**1a** SS-hexanedioldiether, 14 mg.*

**No**	δ **f2**	δ **f1**	f1	f2	**σ**	**R^2**	**DISTANCE, r_{AB}**
2	7.73	4.05	**A**	**D**	4.26E-05	0.9997	**2.31**
3	7.73	1.58	**A**	**H**	1.02E-05	0.9988	**2.94**
4	7.73	2.92	**A**	**E**	1.03E-06	0.7310	**4.30**
5	7.73	1.78	**A**	**G**	8.58E-06	0.9961	**3.02**
6	7.73	2.8	**A**	**F**	1.82E-06	0.8385	**3.91**
8	7.17	2.81	**B**	**F**	1.68E-06	0.8989	**3.96**
9	7.17	2.93	**B**	**E**	1.77E-05	0.9985	**2.68**
10	7.17	6.79	**B**	**C**	2.61E-05	0.7868	**2.51 ref**
12	6.78	4.05	**C**	**D**	2.63E-05	0.9998	**2.51**
16	4.05	1.79	**D**	**G**	5.25E-05	0.9999	**2.23**
17	4.05	1.58	**D**	**H**	3.78E-05	0.9993	**2.36**

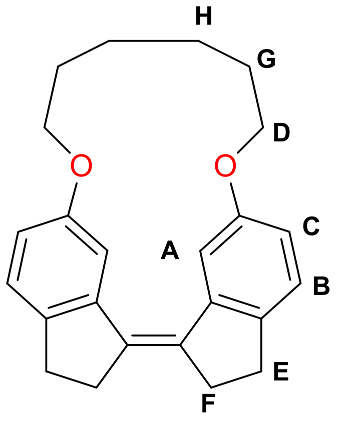

(*Z*)-**1a**, C_6

*Table S2. NOE buildup for (Z)-**1b** SS-octanedioldiether, 4 mg.*

No	δ f2	δ f1	ass. f1	ass. f2	σ	R^2	DISTANCE, r_{AB}
2	7.69	1.56	**A**	**H**	2.03E-06	0.8521	**4.10**
3	7.69	1.8	**A**	**G**	3.63E-06	0.9869	**3.72**
4	7.69	2.92	**A**	**E**	1.16E-06	0.9131	**4.50**
5	7.69	1.44	**A**	**I**	2.51E-06	0.9564	**3.96**
6	7.68	2.8	**A**	**F**	2.23E-06	0.9946	**4.04**
7	7.68	3.95	**A**	**D**	4.96E-05	0.9998	**2.41**
9	7.18	6.74	**B**	**C**	3.86E-05	0.9846	**2.51 (ref)**
10	7.17	3.96	**B**	**D**	1.5E-06	0.9730	**4.31**
11	7.17	2.81	**B**	**F**	3.28E-06	0.3621	**3.79**
12	7.17	2.92	**B**	**E**	2.03E-05	0.8102	**2.79**
14	6.74	1.82	**C**	**G**	1.4E-06	0.8510	**4.36**
15	6.74	1.56	**C**	**H**	7.52E-07	0.7091	**4.84**
17	6.73	3.95	**C**	**D**	3.87E-05	0.9993	**2.51**
20	3.96	1.81	**D**	**G**	5.36E-05	1.0000	**2.38**
22	3.95	1.44	**D**	**I**	2.73E-05	0.9999	**2.66**
25	3.95	1.56	**D**	**H**	1.99E-05	0.9996	**2.80**
32	1.82	1.54	**G**	**H**	2.95E-05	0.9961	**2.62**
34	1.81	1.43	**G**	**I**	3.61E-05	0.9994	**2.54**

Data with greyed R^2 values not taken into account.

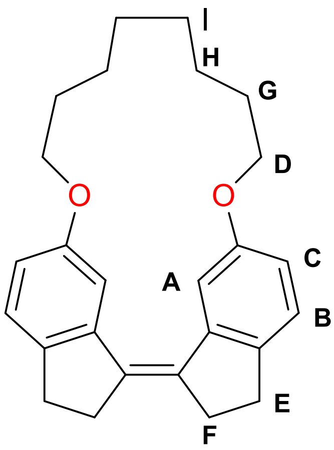

(*Z*)-**1b**, C_8

*Table S3. NOE buildup for (Z)-**1c** SS-decanedioldiether, 12 mg.*

No	δ f2	δ f1	ass. f1	ass. f2	σ	R^2	DISTANCE, r_{AB}
1	7.65	2.91	**A**	**F**	1.07E-06	0.8234	**4.71**
2	7.65	1.77	**A**	**H**	1.45E-06	0.9413	**4.48**
3	7.64	3.91	**A**	**E**	5.8E-05	0.7263	**2.42**
4	7.64	2.8	**A**	**G**	1.22E-06	0.0217	**4.61**
7	7.64	1.54	**A**	**I**	1.22E-06	0.8885	**4.61**
9	7.18	2.93	**C**	**F**	2.59E-05	0.9921	**2.77**
10	7.18	2.81	**C**	**G**	3.43E-06	0.9740	**3.88**
12	7.18	6.75	**C**	**D**	4.67E-05	0.9827	**2.51 (ref)**
13	7.17	3.95	**C**	**E**	9.02E-07	0.0703	**4.85**
14	6.76	3.89	**D**	**E**	5.22E-05	0.9995	**2.46**
17	6.75	1.54	**D**	**I**	4.35E-07	0.2727	**5.47**
19	6.74	1.77	**D**	**H**	2.17E-06	0.9475	**4.19**
22	3.91	1.53	**E**	**I**	2.41E-05	0.9994	**2.80**
25	3.91	1.76	**E**	**H**	6.65E-05	1.0000	**2.37**
26	3.9	2.81	**E**	**G**	1.49E-07	0.0879	**6.55**

Data with greyed R^2 values not taken into account.

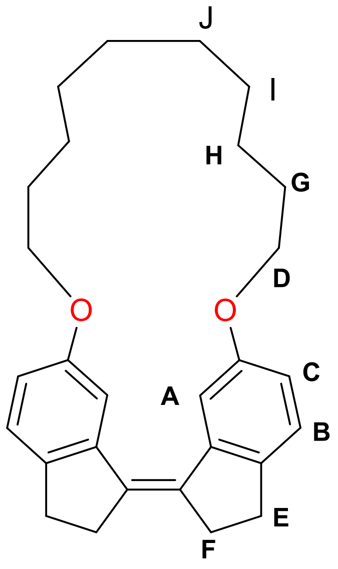

(*Z*)-**1c**, C_{10}

*Table S4. NOE buildup for (Z)-**1d** SS-dodecanedioldiether, 10 mg.*

No	δ f2	δ f1	ass. f1	ass. f2	σ	R^2	DISTANCE, r_{AB}
1	7.63	3.89	A	D	6.3653E-05	0.9999	2.40
2	7.63	1.75	A	G	1.3619E-06	0.5091	4.55
3	7.63	2.81	A	F	2.7143E-06	0.9530	4.05
4	7.63	2.92	A	E	2.1838E-06	0.9817	4.20
6	7.18	6.75	B	C	4.8100E-05	0.9604	2.51 (ref)
7	7.17	2.81	B	F	3.9361E-06	0.8914	3.81
9	7.17	2.92	B	E	2.6125E-05	0.9971	2.78
10	6.75	7.18	C	B	4.8100E-05	0.9604	2.51
12	6.75	3.89	C	D	4.5482E-05	0.9978	2.53
13	3.9	7.63	D	A	6.3653E-05	0.9999	2.40
14	3.89	1.4	D	I	1.1242E-05	0.9786	3.20
15	3.89	1.74	D	G	6.3306E-05	0.9987	2.40
17	3.89	1.49	D	H	3.0916E-05	0.9935	2.70
26	1.75	1.34	G	J	1.2303E-05	0.6422	3.15
27	1.74	1.39	G	I	2.6610E-05	0.9793	2.77

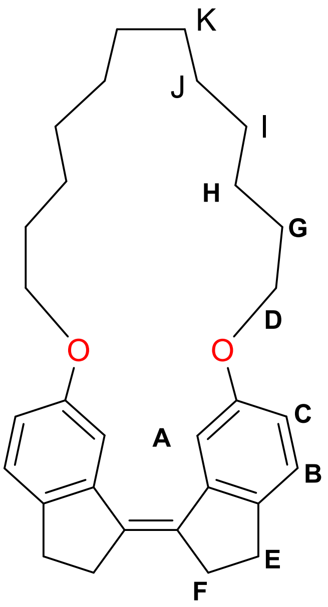

(*Z*)-**1d**, C_{12}

5. DFT calculations

5.1 Energies

The calculated energies for compound 1a-d are shown in Table S5.

Table S5. The calculated energies for compound *(Z)*-**1a–d** and *(E)*-**1a–d** and the open reference.

B3LYP:

Stiff-stilbene	G_{cis} (au)	G_{trans} (au)	G_{cis} (kcal/mol)	G_{cis} (kJ/mol)	G_{trans} (kcal/mol)	G_{trans} (kJ/mol)	ΔG (au)	Δ*G* (kcal/mol)	Δ*G* (kJ/mol)
***n* = 3**	-1,08E+03	-1,08E+03	-6,78E+05	-2,84E+06	-6,78E+05	-2,84E+06	4,08E-02	25,58	107,08
***n* = 4**	-1,16E+03	-1,16E+03	-7,27E+05	-3,05E+06	-7,27E+05	-3,05E+06	1,62E-02	10,14	42,46
***n* = 5**	-1,24E+03	-1,24E+03	-7,77E+05	-3,25E+06	-7,77E+05	-3,25E+06	2,03E-03	1,28	5,34
***n* = 6**	-1,32E+03	-1,32E+03	-8,26E+05	-3,46E+06	-8,26E+05	-3,46E+06	-8,98E-04	-0,56	-2,36
**Reference**	-1,15E+03	-1,15E+03	-7,22E+05	-3,02E+06	-7,22E+05	-3,02E+06	-5,71E-03	-3,58	-15,00

5.2 Ring strain

The ring strain for compounds **1a-d** were calculated using the isodesmic reaction shown in Figure S47.

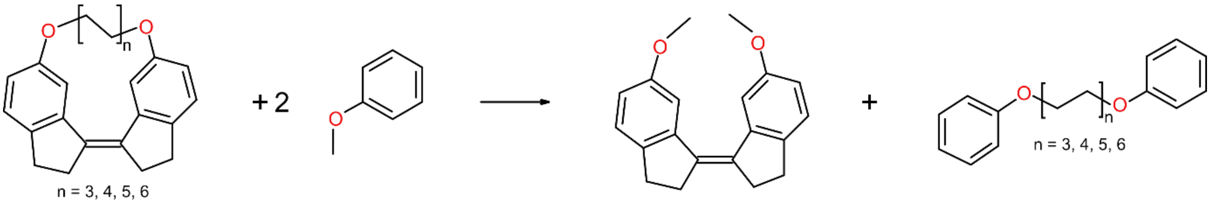

Figure S47. Isodesmic reaction for the calculation of ring strain in the compound *(Z)*-**1a–d** and *(E)*-**1a–d**.

The calculated energies for the compounds in the isodesmic reaction are shown in Table S6.

Table S6. The calculated energies for the species in the isodesmic reaction shown in *Figure S47.*

Stiff-stilbene	Cyclic cis	Cyclic trans	Phenylether	->	Open cis	Open trans	Linker
***n* = 3**	-1080,69	-1080,65	-346,78		-924,63	-924,64	-849,63
***n* = 4**	-1159,32	-1159,30	-346,78		-924,63	-924,64	-928,27
***n* = 5**	-1237,95	-1237,95	-346,78		-924,63	-924,64	-1006,90
***n* = 6**	-1316,58	-1316,59	-346,78		-924,63	-924,64	-1085,54

The calculated ring strains for the compounds *(Z)*-**1a-d** and *(E)*-**1a-d** are shown in Table S7.

Table S7. The calculated ring strain for compound *(Z)*-**1a–d** and *(E)*-**1a–d**.

**Stiff-stilbene**	**Ring strain cis au**	**Ring strain trans au**	**Ring strain cis kcal/mol**	**Ring strain trans kcal/mol**	**Ring strain cis kJ/mol**	**Ring strain trans kJ/mol**	**Cis-Trans kJ/mol**
***n* = 3**	-0,018	-0,061	-11,20	-38,00	-46,88	-159,11	112,23
***n* = 4**	-0,020	-0,038	-12,44	-23,81	-52,07	-99,68	47,61
***n* = 5**	-0,025	-0,029	-15,58	-18,08	-65,21	-75,70	10,49
***n* = 6**	-0,023	-0,024	-14,44	-15,10	-60,45	-63,23	2,79

5.3 Optimized geometries

Coordinates compound *(Z)*-**1a**:

```
C     2.76129845    0.40718128   -0.17315222
C     2.22047485   -0.96879609   -0.09738357
C     3.24951808   -1.89549972   -0.37237364
C     4.53308478   -1.18912002   -0.73668620
C     4.28226719    0.26509188   -0.27109918
C     2.16386893    1.62429220   -0.09837610
C     0.74190928    2.01766752   -0.09403134
C     0.61031056    3.31097189    0.45778253
C     1.96607769    3.87342650    0.82074837
C     2.95119054    2.92061219    0.09344949
C    -0.38775907    1.35495617   -0.58173297
C    -1.65199764    1.93576620   -0.43043873
C    -0.64905554    3.88451727    0.60284499
C     0.95967365   -1.43754719    0.30939069
C     0.73021880   -2.81452894    0.40008249
C     3.01681942   -3.26543143   -0.26708926
H     5.41319904   -1.64097055   -0.26772343
H     4.69869467   -1.22364495   -1.82218113
H     4.73721144    0.42317831    0.71691177
H     4.72516250    1.00373896   -0.94519093
H     2.08540573    4.91959944    0.52064398
H     2.12783333    3.83220387    1.90660009
H     3.22540093    3.34727627   -0.88199348
H     3.87929448    2.77207146    0.65158027
H    -0.32100776    0.39891637   -1.08481146
H    -0.75735152    4.87391010    1.04086446
H     0.17361041   -0.75400817    0.59766623
H     3.81279171   -3.97613596   -0.47585783
C     1.75494004   -3.72828177    0.10970525
C    -1.79040189    3.19273237    0.17757814
H    -2.77096411    3.64169988    0.29359792
H     1.54682691   -4.79060702    0.19295200
C    -3.94300611    1.23496013   -0.20759567
H    -4.43362980    2.21181518   -0.30910213
H    -3.73976646    1.07012870    0.85898781
C    -4.84335295    0.14842689   -0.78183846
H    -5.09038197    0.41742868   -1.81673668
H    -5.78535421    0.17656804   -0.21813118
C    -4.26543814   -1.27783441   -0.76375645
```

H	-4.98098526	-1.92109225	-1.28907728
H	-3.35311956	-1.28982868	-1.36898625
C	-3.98858591	-1.87258530	0.64201527
H	-4.40124029	-2.88880439	0.68706020
H	-4.53941371	-1.29779509	1.39756490
C	-2.51023541	-1.95103656	1.07215382
H	-2.05562680	-0.95359736	1.07807753
H	-2.46280755	-2.32887278	2.10114826
C	-1.68804944	-2.89995542	0.19576555
H	-2.26155818	-3.81850680	0.01810098
H	-1.45336209	-2.45915887	-0.77895620
O	-0.46573816	-3.33667728	0.82918807
O	-2.70444723	1.21599901	-0.93403621

Coordinates compound *(E)*-**1a**:

C	0.54716192	-1.85357162	-0.39615919
C	0.62239489	-2.00131722	-1.90288656
C	2.09796864	-2.38495553	-2.15538794
C	2.78872096	-1.62606063	-1.05524279
C	1.89393107	-1.45738222	0.01380473
C	-0.55780330	-1.89217328	0.38050085
C	-0.62445237	-2.01463194	1.89018599
C	-2.09942996	-2.38811274	2.15680106
C	2.17793885	-0.55477726	1.03755849
C	3.29524432	0.25789940	0.91524395
C	4.24886875	0.01882795	-0.07900828
C	3.99729121	-0.94008211	-1.06374513
H	-2.95778399	3.36555934	-1.72136716
H	-0.10389654	-2.71792649	-2.30314219
H	2.44880055	-2.10913644	-3.15559184
H	2.23158489	-3.47111706	-2.04560151
H	-0.43515602	-1.03592198	2.36156638
H	0.10140854	-2.72672942	2.29997867
H	-2.44111568	-2.10441853	3.15799399
H	-2.24143451	-3.47390881	2.05379266
H	1.45484482	-0.31811100	1.81424119
H	5.13291550	0.65132206	-0.13473964
H	4.70178013	-1.06916770	-1.88442121
O	3.33234151	1.38175679	1.70084857
C	3.11320141	2.59127281	0.95432595
H	2.96084871	3.36530249	1.71753551
H	4.03419098	2.84763437	0.40688943
C	1.94973787	2.57827893	-0.03196671
H	2.09799292	1.77504096	-0.77121423
H	2.03598529	3.51704087	-0.60092478
C	0.53940661	2.48784821	0.54251363
H	0.42836204	1.57414634	1.14898730
H	0.37125632	3.33208694	1.23119675
C	-0.53690674	2.49016413	-0.54569720
H	-0.43007115	1.57685374	-1.15361269
H	-0.36666637	3.33466623	-1.23353413
C	-1.94631993	2.58404670	0.03026581
H	-2.09449024	1.78261565	0.77148524
H	-2.03110072	3.52427775	0.59696608
C	-3.11006780	2.59423177	-0.95540442
H	0.43921087	-1.03092346	-2.39399701
H	-4.03110821	2.85270961	-0.40908700
C	-2.18478270	-0.56266401	-1.03823066
H	-1.46462272	-0.33162362	-1.81944779
C	-4.24304317	0.03119422	0.09208390
H	-5.12067003	0.67193110	0.15400853
C	-3.99214911	-0.92849554	1.07598078
H	-4.69055663	-1.04926833	1.90314737
C	-2.79144326	-1.62798584	1.05801588
C	-1.90249480	-1.46899303	-0.01746242
C	-3.29416037	0.26010991	-0.90901274
O	-3.32776485	1.38212309	-1.69754479

Coordinates compound *(Z)*-**1b**:

C	-3.18229949	-0.20513389	0.15612866
C	-2.32086586	-1.39135906	-0.03551873
C	-2.94406428	-2.52396590	0.53194095
C	-4.27885873	-2.15198891	1.13571633
C	-4.55837282	-0.74747246	0.54250853
C	-2.92173859	1.12441161	0.06674908
C	-1.65198680	1.86500605	-0.07595852
C	-1.91847013	3.22455293	-0.35752518
C	-3.40685514	3.46752214	-0.45846100
C	-4.02165363	2.18417454	0.15720975
C	-0.33374536	1.44768131	0.11252956
C	0.72075756	2.35609026	-0.05008408
C	-0.86974206	4.12773861	-0.48964156
C	-1.10754705	-1.54629259	-0.72201810
C	-0.49167198	-2.79964897	-0.76365418
C	-2.32905534	-3.77333362	0.48003461
H	-5.06519115	-2.87779925	0.90469614
H	-4.20620453	-2.09757506	2.23053082
H	-5.19221869	-0.84276332	-0.35069729
H	-5.08631044	-0.09384027	1.24200948
H	-3.72479992	4.37929763	0.05779460
H	-3.71304640	3.57374039	-1.50812032
H	-4.28287570	2.36537251	1.20948472
H	-4.94056307	1.87574113	-0.34929236
H	-0.08747374	0.43382152	0.39561810
H	-1.06979909	5.17559386	-0.70075965
H	-0.63642787	-0.72580590	-1.24731214
H	-2.80984768	-4.64239099	0.92262740
C	-1.09154973	-3.91220401	-0.15640732
C	0.45939866	3.70204148	-0.34675599
H	1.26679199	4.41690125	-0.45511078
H	-0.59837356	-4.87813370	-0.21250696
C	3.12964073	2.63022939	-0.06219160
H	3.18461967	3.36081991	0.75812080
H	3.06269172	3.18572906	-1.00691156
C	4.32879424	-1.60850131	0.97400441
H	5.38108046	-1.74532979	1.26109352
H	3.78088292	-2.41860005	1.47419069
C	4.22551276	-1.80918691	-0.55086444
H	4.68764618	-2.77572715	-0.79697451
H	4.83340481	-1.05405497	-1.06285631
C	2.80815850	-1.77959978	-1.15074213
H	2.31754603	-0.82443643	-0.93138158
H	2.88640370	-1.84782032	-2.24317644
C	1.91166289	-2.92452404	-0.68278739
H	2.41347885	-3.88928134	-0.83656784
H	1.66644526	-2.83994064	0.38096544
O	0.69019007	-2.93684080	-1.46084040
O	1.96534692	1.81263704	0.10054899
C	4.67994508	0.98190749	1.22591623
H	5.73981628	0.69245196	1.20955008
H	4.58451570	1.70051308	2.05173449
C	3.83719602	-0.27060311	1.56422464
H	3.85166306	-0.38973745	2.65574563
H	2.79173446	-0.09368153	1.29703948
C	4.36560306	1.73655346	-0.08231621
H	5.20927646	2.40069942	-0.31188815

H	4.28168316	1.04529829	-0.92753211

Coordinates compound *(E)*-**1b**:

C	0.22362244	-1.34905435	-0.61237470
C	0.00642479	-1.19135223	-2.11827428
C	1.25817820	-1.86500049	-2.75746232
C	2.30912950	-1.68544773	-1.68212231
C	1.67349998	-1.50608839	-0.44015921
C	-0.73138884	-1.49742678	0.33390201
C	-0.56539733	-2.07968763	1.73700493
C	-1.93390779	-2.77289443	2.02462257
C	-2.89427876	-1.97246818	1.16801180
C	-2.17777335	-1.32655625	0.15026171
C	2.40334407	-1.08275852	0.67627622
C	3.74829958	-0.75296695	0.52221229
C	4.41351275	-1.03564686	-0.68141032
C	3.68985550	-1.51762448	-1.78320647
C	-4.83387825	-0.64102221	0.60021212
C	-4.24873392	-1.67686622	1.33919703
H	-0.93523587	-1.63116691	-2.45823424
H	1.54098730	-1.41297380	-3.71269767
H	1.05737660	-2.92850766	-2.94618883
H	-0.41219944	-1.27864862	2.47338565
H	0.28464663	-2.76318540	1.81583497
H	-2.19658739	-2.76167502	3.08674211
H	-1.89533565	-3.82498865	1.71029218
H	1.91267565	-0.83506413	1.61050070
H	5.47017306	-0.80440519	-0.77969133
H	4.19933412	-1.66997794	-2.73172187
H	-5.85274027	-0.32430699	0.80289862
H	-4.83446026	-2.17644761	2.10719972
O	4.33814902	-0.05866141	1.55914274
C	4.69081936	1.32241706	1.27442287
H	4.86439367	1.75821641	2.26368264
H	5.64501487	1.34215024	0.73048767
C	3.66242209	2.14274973	0.48115802
H	4.14103800	3.11194698	0.28485934
H	3.53253399	1.67418352	-0.50142252
C	2.27444402	2.37032059	1.11488982
H	1.91529951	1.43407097	1.55456222
H	2.35428459	3.08783146	1.94291883
C	1.23761079	2.85389231	0.07797466
H	1.29475770	2.17977100	-0.78782867
H	1.52181076	3.84712718	-0.29741080
C	-0.22365629	2.87641676	0.57157306
H	-0.41414824	1.95645293	1.14098702
H	-0.37635629	3.70738653	1.27399557
H	-0.00632453	-0.13006194	-2.40244681
C	-2.71851250	2.74522592	-0.12336340
H	-2.73161592	1.99317082	0.67150172
H	-3.10920096	3.66586367	0.32500288
C	-3.67592492	2.27354419	-1.24968568
H	-4.32682538	3.08362790	-1.58668486
H	-3.11334564	1.92779567	-2.12598871
C	-1.25423021	2.94844020	-0.57548114
H	-1.00086601	2.16317431	-1.30037446
H	-1.15279467	3.89918140	-1.11612852
C	-2.77238115	-0.32956306	-0.63348659
H	-2.16761947	0.22791344	-1.33510085

C	-4.07019519	0.08453050	-0.33324005
O	-4.60741126	1.24252022	-0.83434866

Coordinates compound *(Z)*-**1c**:

C	-3.55438936	-0.35831139	0.08417511
C	-2.61811654	-1.49582298	-0.03158565
C	-3.26331508	-2.68474349	0.37330852
C	-4.67963070	-2.39853559	0.81572917
C	-4.94258328	-0.97660185	0.25913072
C	-3.35688959	0.98375795	0.04384524
C	-2.12435913	1.80012122	0.05541014
C	-2.43305634	3.12528767	-0.32770471
C	-3.90470901	3.26159323	-0.64303991
C	-4.51568099	1.97981789	-0.02172921
C	-0.82734406	1.48610604	0.47061888
C	0.17228656	2.47041175	0.45294816
C	-1.43582119	4.09304836	-0.35568224
C	-1.31972392	-1.56367179	-0.55657744
C	-0.66858545	-2.79734829	-0.63025109
C	-2.60194583	-3.90955401	0.30999787
H	-5.39620424	-3.14035155	0.44883810
H	-4.75085407	-2.40014089	1.91203211
H	-5.44812278	-1.04862774	-0.71439421
H	-5.58777265	-0.38279652	0.91217340
H	-4.34292843	4.18057795	-0.24038561
H	-4.07287411	3.27631722	-1.72871703
H	-4.88192603	2.19910893	0.99140761
H	-5.36551863	1.59931529	-0.59474786
H	-0.56334417	0.50289549	0.83931761
H	-1.66517274	5.11148143	-0.66026159
H	-0.80895043	-0.69125420	-0.94423364
H	-3.10276056	-4.82188035	0.62487328
C	-1.29562467	-3.96949019	-0.18581104
C	-0.12474071	3.77252567	0.02159013
H	0.64079433	4.53890521	-0.00642917
H	-0.77273289	-4.91818649	-0.26200130
C	4.26927034	-2.00630389	1.19635356
H	5.25908876	-2.20430011	1.63098518
H	3.55566501	-2.54443626	1.83480601
C	4.23964385	-2.63094557	-0.21194780
H	4.45421464	-3.70365871	-0.10813866
H	5.05876160	-2.22140187	-0.81529836
C	2.93303960	-2.45995137	-1.00387208
H	2.75728035	-1.40218445	-1.22784454
H	3.03160491	-2.97035884	-1.97024571
C	1.69321375	-3.00316116	-0.30507262
H	1.81292964	-4.06696859	-0.05174614
H	1.48794668	-2.45750755	0.62381190
O	0.58252731	-2.83357879	-1.20940865
O	1.39129116	2.05395077	0.91402944
C	5.01033763	0.46942617	0.71473380
H	6.01294070	0.03285501	0.82820608
H	5.02902907	1.38667921	1.31572351
C	3.97384186	-0.49864810	1.32971380
H	3.92015901	-0.28947427	2.40619815
H	2.97291125	-0.27218461	0.94436693
C	4.82621152	0.86442396	-0.76482320
H	5.65235085	1.53740620	-1.03592742
H	4.95040336	-0.02454327	-1.39558044

C	3.50126309	1.53894963	-1.17441449
H	2.65678409	0.86774563	-0.98170516
H	3.53455856	1.66159578	-2.26434308
C	3.18098890	2.91906270	-0.56452220
H	2.50252614	3.44738126	-1.24478602
H	4.09056158	3.53279462	-0.50368524
C	2.54450238	2.90570302	0.82925528
H	3.22838267	2.49529928	1.57434553
H	2.29121896	3.92404829	1.14775931

Coordinates compound *(E)*-**1c**:

C	0.45291401	-1.54709303	-0.50368637
C	0.15916832	-1.71087545	-1.99403534
C	1.44704109	-2.35760545	-2.57834363
C	2.51575427	-1.93258520	-1.59564477
C	1.91885637	-1.54154832	-0.38056276
C	-0.45296965	-1.54704503	0.50382287
C	-0.15915164	-1.71012296	1.99423835
C	-1.44698337	-2.35664535	2.57889439
C	-2.51575562	-1.93212751	1.59602769
C	-1.91890948	-1.54159514	0.38075553
C	2.69583995	-0.96884525	0.63308028
C	4.05637819	-0.74710596	0.41032183
C	4.66800353	-1.20574346	-0.76607106
C	3.89228727	-1.80609198	-1.76792096
C	-4.66803471	-1.20555478	0.76622744
C	-3.89227624	-1.80546758	1.76831372
H	-0.73749460	-2.30662997	-2.18453534
H	1.65603141	-2.03459527	-3.60278836
H	1.34549382	-3.45138299	-2.59941310
H	-0.00357113	-0.72951923	2.46484623
H	0.73754334	-2.30578528	2.18492970
H	-1.65598139	-2.03310987	3.60316949
H	-1.34535156	-3.45040315	2.60055710
H	2.25271748	-0.58349162	1.54337811
H	5.73134379	-1.04679337	-0.91850781
H	4.36500456	-2.11867122	-2.69593771
H	-5.73136638	-1.04651634	0.91864116
H	-4.36499368	-2.11774559	2.69643182
O	4.73450291	-0.02784486	1.37213057
C	5.33624709	1.21822217	0.94273260
H	5.65503759	1.69065829	1.87733393
H	6.23733566	1.00973067	0.35059381
C	4.40255447	2.13652120	0.14690466
H	4.98453107	3.02780740	-0.12190497
H	4.16043505	1.64071972	-0.80055562
C	3.09323569	2.53683906	0.85646247
H	2.75347744	1.69868163	1.47441832
H	3.27950503	3.36914764	1.54854390
C	1.96813615	2.89782830	-0.13170267
H	1.91848044	2.10312257	-0.88891273
H	2.22422682	3.81796124	-0.67591358
C	0.57643409	3.03088373	0.51053603
H	0.42836611	2.17993447	1.19060334
H	0.52934014	3.93287837	1.13685288
H	0.00362189	-0.73048102	-2.46511025
C	-1.96816988	2.89775896	0.13143102
H	-1.91840125	2.10286159	0.88844256
H	-2.22447428	3.81769973	0.67588462
C	-0.57644916	3.03109294	-0.51065399
H	-0.42840942	2.18037597	-1.19102963
H	-0.52929711	3.93329559	-1.13666980
C	-2.69587838	-0.96902856	-0.63297982
H	-2.25274857	-0.58398880	-1.54340188
C	-4.05644179	-0.74737748	-0.41036384
O	-4.73458974	-0.02833178	-1.37229533
C	-4.40251916	2.13645197	-0.14736605
H	-4.16041476	1.64068153	0.80011339

H	-4.98451067	3.02774474	0.12139829
C	-3.09314683	2.53675524	-0.85684930
H	-3.27930739	3.36897592	-1.54906708
H	-2.75331650	1.69850203	-1.47463737
C	-5.33616813	1.21802554	-0.94315950
H	-6.23734693	1.00980419	-0.35104961
H	-5.65478983	1.69042001	-1.87783865

Coordinates compound *(Z)*-**1d**:

C	3.86985342	0.50896757	0.34620417
C	2.85707089	1.58443456	0.38362202
C	3.36722620	2.68842984	1.10478329
C	4.74707188	2.38124741	1.63903819
C	5.18159129	1.16415143	0.78410148
C	3.80184395	-0.78247862	-0.06060100
C	2.65922278	-1.68146337	-0.34786508
C	3.12537005	-2.78952487	-1.09341322
C	4.60900254	-2.67099989	-1.35427912
C	5.05243851	-1.62963458	-0.29621941
C	1.34217077	-1.66712845	0.12760716
C	0.48924834	-2.74931176	-0.15647944
C	2.25533990	-3.81512961	-1.43611332
C	1.63426838	1.69644083	-0.28404375
C	0.92053815	2.90323333	-0.22264948
C	2.63071438	3.86322451	1.20017337
H	5.43469136	3.22893437	1.55581844
H	4.70543159	2.10457855	2.70166261
H	5.73960117	1.51385408	-0.09605884
H	5.83508412	0.47783192	1.32945758
H	5.13484229	-3.62664475	-1.26284235
H	4.80513995	-2.28931299	-2.36592706
H	5.34104635	-2.14856495	0.62907791
H	5.91180487	-1.03709663	-0.62042770
H	0.97218408	-0.86954307	0.76289825
H	2.59965705	-4.65574742	-2.03389603
H	1.23274057	0.88968825	-0.88668914
H	3.01589837	4.71164787	1.76085046
C	1.39966627	3.97824418	0.54142453
C	0.93045180	-3.79635560	-0.98262990
H	0.26942895	-4.61121197	-1.25048287
H	0.84403990	4.90779140	0.59392187
C	-5.25996407	1.48817016	0.74305558
H	-6.25255120	1.53831938	1.21182826
H	-4.66037763	2.26098716	1.24187120
C	-5.40488984	1.85822874	-0.75112060
H	-5.63249723	2.93087428	-0.81331543
H	-6.27993290	1.34779985	-1.17417346
C	-4.19514606	1.52103368	-1.64506036
H	-4.13839860	0.43005273	-1.74577987
H	-4.38903818	1.89893777	-2.65866728
O	-0.21431083	2.95391184	-0.97975738
O	-0.74035653	-2.71801458	0.44455381
C	-5.34859713	-1.13041867	0.57709163
H	-5.85550501	-0.93481939	-0.37633097
H	-6.14023449	-1.38628473	1.29521768
C	-4.60495848	0.13068504	1.05297213
H	-4.45128163	0.06834662	2.13745799
H	-3.59997867	0.11992636	0.61771389
C	-4.40502315	-2.33486015	0.38304042
H	-4.99682815	-3.22468166	0.12618617
H	-3.76341674	-2.13151105	-0.48522185
C	-2.82026792	2.01754829	-1.16787481
H	-2.70073791	1.80859038	-0.09886868
H	-2.04607869	1.42695390	-1.66734367
C	-2.50298847	3.50008831	-1.40537725
H	-2.39461556	3.69285394	-2.48050543

H	-3.31465854	4.14664183	-1.04791550
C	-1.22966473	3.91971328	-0.67248128
H	-1.39985254	3.93126495	0.41288319
H	-0.90724113	4.92420890	-0.97818112
C	-3.51927233	-2.64184484	1.59910704
H	-2.88598032	-1.77644276	1.82058815
H	-4.15627914	-2.79013280	2.48214157
C	-2.61842542	-3.87717888	1.45354481
H	-2.07336810	-4.03151632	2.39324318
H	-3.23515118	-4.77370606	1.30118093
C	-1.60219846	-3.86119668	0.31500371
H	-1.01023869	-4.78166484	0.37130250
H	-2.09016992	-3.83627236	-0.66841493

Coordinates compound *(E)*-**1d**:

C	-0.42072052	-1.52605438	0.59895116
C	0.10917158	-1.43722478	2.02908485
C	-1.07691726	-1.87770781	2.92944794
C	-2.28569161	-1.65471476	2.04961680
C	-1.89049067	-1.51072721	0.70294796
C	0.31881870	-1.68340576	-0.52823927
C	-0.23458696	-2.00588371	-1.91590676
C	0.93914710	-2.67382977	-2.67704699
C	2.15547919	-2.20708865	-1.91419573
C	1.78827553	-1.69113780	-0.65668744
C	-2.84846677	-1.21324430	-0.27487204
C	-4.18575130	-1.04105613	0.09581377
C	-4.58549059	-1.23274864	1.42637959
C	-3.62813249	-1.54311599	2.40165321
C	4.45447910	-1.62456296	-1.48068624
C	3.49148223	-2.19490978	-2.31147036
H	1.00069166	-2.05089291	2.18390824
H	-1.12498925	-1.31738806	3.86860486
H	-0.98066201	-2.93913640	3.19585129
H	-0.52786112	-1.08266510	-2.43398427
H	-1.12130707	-2.64369083	-1.87337225
H	0.96867884	-2.39623106	-3.73565431
H	0.84844896	-3.76814470	-2.63742584
H	-2.58014609	-1.03267148	-1.30776174
H	-5.62814168	-1.11118469	1.70320690
H	-3.93880932	-1.66372648	3.43673848
H	5.49552806	-1.56892321	-1.78383964
H	3.78554607	-2.59850409	-3.27734023
O	-5.05617932	-0.67635007	-0.90751689
C	-5.89087580	0.48361527	-0.68905387
H	-6.38029184	0.63621995	-1.65612974
H	-6.67295395	0.25378683	0.04662651
C	-5.11640926	1.73158673	-0.25621108
H	-5.84002388	2.55259211	-0.17154863
H	-4.73303020	1.56216157	0.75690861
C	-3.94336878	2.11815422	-1.17981240
H	-3.51479773	1.20175055	-1.59933497
H	-4.30934652	2.70216812	-2.03463250
C	-2.82672853	2.87598056	-0.43932051
H	-2.66627552	2.37611396	0.52598160
H	-3.15159110	3.89824984	-0.19934663
C	-1.47729496	2.90284560	-1.17666979
H	-1.26280788	1.88529294	-1.53250298
H	-1.53739757	3.53404350	-2.07420111
H	0.38506105	-0.40120299	2.26957105
C	1.08382539	3.02553373	-0.82905460
H	1.08489271	1.98140660	-1.17235366
H	1.30124092	3.63899242	-1.71477585
C	-0.31352568	3.35342844	-0.27704660
H	-0.41978120	2.84488124	0.69205404
H	-0.39325612	4.42868379	-0.06343477
C	2.75907733	-1.13370949	0.19584056
H	2.47272429	-0.68998320	1.13798850
C	4.08805473	-1.06999398	-0.24013374
O	5.10287859	-0.45308042	0.43406459
C	3.56503059	2.63702420	-0.20806031
H	3.42524425	1.66648222	-0.69973962

H	4.00507011	3.30159680	-0.96350985
C	2.19420364	3.18892077	0.22154400
H	2.29727858	4.24723787	0.50107748
H	1.86683826	2.67071025	1.13286404
C	4.55749131	2.47243637	0.96228698
H	5.56233960	2.28735756	0.56318766
H	4.61641621	3.42483636	1.50475376
C	4.18195246	1.34675557	1.95498609
H	4.40422988	1.66074273	2.98292139
H	3.10104318	1.17974382	1.92911066
C	4.93217896	0.01713615	1.78026548
H	5.96151345	0.13735675	2.13182876
H	4.46178976	-0.76642879	2.38921910
